# Molecular and Conventional Tests in Onychomycosis: A Systematic Review of Paired Diagnostic Discordance and Clinical Interpretation

**DOI:** 10.7759/cureus.117135

**Published:** 2026-09-29

**Authors:** Stefan Bigge, Bianca Bigge

**Affiliations:** 1 Dermatology, Praxis Bigge Ungerechts, Cologne, DEU; 2 Health Sciences, Carl Remigius Medical School, Düsseldorf, DEU

**Keywords:** dermatophytes, diagnostic discordance, fungal culture, histopathology, molecular diagnostics, non-dermatophyte molds, onychomycosis, polymerase chain reaction

## Abstract

Discordant laboratory results complicate the diagnosis of onychomycosis because molecular assays, culture, microscopy, and histopathology detect different features of fungal infection. We systematically reviewed human nail studies comparing molecular testing of primary nail material with conventional methods. PubMed was searched without language or publication-date restrictions on August 29, 2026, and updated on September 20, 2026. Six supplementary Google Scholar searches and backward and forward citation searches were also performed. Of 102 reports sought, 101 were assessed and 97 were included; one could not be retrieved. Seventy-six reports provided paired 2 × 2 data, and 21 provided marginal or narrative evidence. The 174 extracted comparisons were summarized descriptively. In the initial exploratory set of 40 culture comparisons from 37 reports, molecular-only positives outnumbered culture-only positives in 38 comparisons. The median net detection difference was 16.1 percentage points (IQR, 11.5-22.9). A broader post hoc analysis selecting one comparison per eligible report included 50 reports and yielded a median of 16.6 points (IQR, 10.4-28.1); excluding potentially overlapping cohorts yielded 16.2 points. Microscopy or histopathology yielded more additional positives in four of six exploratory structural comparisons. Selection bias, previous treatment, differences in assay coverage, and incomplete verification limited interpretation. The single-reviewer process, PubMed-centered search, and post hoc analytic decisions further limit the synthesis. Molecular assays frequently detected fungal targets in culture-negative nails, but these additional findings cannot be equated with viable infection or superior diagnostic accuracy. Discordant results require assessment of the organism, assay coverage, specimen quality, treatment history, and corroborating evidence. These data support the complementary use of diagnostic methods; they do not establish a universal detection gain or a validated treatment algorithm.

## Introduction and background

Clinical examination alone cannot reliably distinguish onychomycosis from traumatic or inflammatory nail dystrophy. Laboratory confirmation matters when the diagnosis will determine prolonged topical or systemic antifungal treatment [1-4]. Available methods include culture, direct microscopy, periodic acid-Schiff (PAS) histopathology, and molecular assays. PAS highlights fungal structures in nail keratin; PCR amplifies selected fungal DNA targets. Non-dermatophyte molds (NDMs), which are filamentous fungi other than dermatophytes, require particular care in interpretation because their presence may reflect contamination or colonization.

A positive result has a different meaning for each method. Culture recovers an organism capable of growth under the conditions used. Microscopy and PAS show fungal structures but generally do not identify species or establish viability. Detection of fungal DNA provides taxonomic information without proving tissue invasion or causality. A molecular-positive/culture-negative result, or a PAS-positive/PCR-negative result, therefore cannot be classified as an error solely because another test disagrees [1,3,4].

Previous reviews describe the available technologies, and a 2024 network meta-analysis compared diagnostic modalities [3-5]. Here, we examined the direction and magnitude of disagreement within paired nail specimens. We compared molecular tests applied directly to human nail material with culture, microscopy, PAS histopathology, or eligible conventional composite comparators. The principal outcome was the difference between molecular-only and comparator-only positive results. We also examined how sampling, previous treatment, assay coverage, and pathogen attribution affect the clinical meaning of discordance.

## Review

Methods

Review Design

Protocol v0.1 was finalized before full-text extraction but was not prospectively registered. Reporting follows Preferred Reporting Items for Systematic Reviews and Meta-Analyses (PRISMA) 2020, with a completed checklist supplied [6]. We added a formalized report-level appraisal and sensitivity analyses during peer-review revision. These additions were post hoc; the original eligibility framework was retained.

Information Sources and Search Strategy

Table 1 gives the search strategies, dates, restrictions, and yields. PubMed was the primary bibliographic database and source of indexed metadata. The search returned 308 records on August 29, 2026, with no additional records in an identical September 1 update. An identical September 20 update returned 309 records. The additional record was excluded at title/abstract screening because it described a single case without an eligible direct-nail comparison. Google Scholar and citation searches retained their earlier dates. PubMed and Scholar searches GS1-GS5 had no publication-date or language restrictions; GS6 covered recent literature from 2024-2026. PubMed did not require comparator terms, which may be reported only in full-text methods. Exact executable queries and retrieval logs accompany the dataset.

**Table 1:** Search strategies, dates, restrictions, and recorded yields. NGS: Next-Generation Sequencing; PMID: PubMed Identifier; NCBI: National Center for Biotechnology Information.

Source / run and date	Search expression or procedure	Restrictions and recorded yield
PubMed P1, August 29, 2026	("Onychomycosis"{Mesh} OR onychomycosis{tiab} OR "tinea unguium"{tiab} OR "fungal nail"{tiab} OR "fungal nail infection*"{tiab}) AND ("Molecular Diagnostic Techniques"{Mesh} OR "Polymerase Chain Reaction"{Mesh} OR PCR{tiab} OR qPCR{tiab} OR "real-time PCR"{tiab} OR "multiplex PCR"{tiab} OR "molecular diagnos*"{tiab} OR "molecular detect*"{tiab} OR "DNA sequencing"{tiab} OR sequencing{tiab} OR "next generation sequencing"{tiab} OR NGS{tiab} OR nanopore{tiab})	Database inception to search date; no language restriction. 308 records.
PubMed update, September 1, 2026	P1 rerun unchanged. Additional entry-date check: (("Onychomycosis"{Mesh} OR onychomycosis{tiab} OR "tinea unguium"{tiab} OR "fungal nail"{tiab} OR "fungal nail infection*"{tiab}) AND ("Molecular Diagnostic Techniques"{Mesh} OR "Polymerase Chain Reaction"{Mesh} OR PCR{tiab} OR qPCR{tiab} OR "real-time PCR"{tiab} OR "multiplex PCR"{tiab} OR "molecular diagnos*"{tiab} OR "molecular detect*"{tiab} OR "DNA sequencing"{tiab} OR sequencing{tiab} OR "next generation sequencing"{tiab} OR NGS{tiab} OR nanopore{tiab})) AND ("2026/08/30"{EDAT} : "2026/09/01"{EDAT})	308 records in the complete rerun; 0 new records in the entry-date check.
Google Scholar GS1, August 29, 2026	onychomycosis PCR culture	No date or language restriction; 18 returned results screened.
Google Scholar GS2, August 29, 2026	onychomycosis molecular versus conventional diagnosis	No date or language restriction; 8 returned results screened.
Google Scholar GS3, August 29, 2026	tinea unguium PCR culture	No date or language restriction; 19 returned results screened.
Google Scholar GS4, August 29, 2026	onychomycosis sequencing culture	No date or language restriction; 28 returned results screened.
Google Scholar GS5, August 29, 2026	nail dermatophyte PCR microscopy culture	No date or language restriction; 20 returned results screened.
Google Scholar GS6, August 29, 2026	onychomycosis PCR	Additional search focused on 2024-2026, ordered by recency; 19 returned results screened.
Backward citation chasing, September 1, 2026	Reference lists of included primary reports and central reviews/meta-analyses; OpenAlex-indexed references of resolvable seed reports were evaluated.	1,046 indexed reference links before relevance filtering and deduplication.
Forward citation chasing, September 1, 2026	OpenAlex-indexed citing works of the same seed set after Google Scholar cited-by access was temporarily blocked.	1,115 indexed citing links; combined citation-searching yield of 2,161 links, 198 after relevance filtering and deduplication, and 76 unique new candidates.
PubMed P3, September 20, 2026	Complete P1 query rerun unchanged via NCBI ESearch; all PMIDs compared with the original 308-record master set.	No language or publication-date restriction. 309 records; one new record, excluded at title/abstract screening.

P1 denotes the complete predefined PubMed query. PubMed field tags are displayed in braces (e.g., {tiab}) to distinguish search syntax from reference citations; replace braces with square brackets to execute the queries. MeSH, Medical Subject Headings; tiab, title/abstract; EDAT, PubMed entry date. Search terms, Boolean operators, and filters are unchanged. All 112 results returned by the Scholar interface were screened. Raw results and indexed citation links are not counts of unique studies. Citation-searching counts are reported jointly after cross-direction and master-set deduplication.

We compared titles, authors, publication years, DOI/PMID identifiers, and cohort descriptions to identify duplicate or overlapping reports. The Reinhardt thesis was linked to the subsequent Pospischil publication and was not counted as a separate study. Backward and forward citation searching supplemented database retrieval. OpenAlex replaced forward Google Scholar searching after access to the latter was blocked; this procedural change is documented in the search log. Embase, Scopus, and Web of Science were not systematically searched.

Eligibility Criteria

Table 2 lists the inclusion and exclusion criteria. Eligibility for the review was assessed separately from suitability for paired-data synthesis. An otherwise eligible report could contribute narrative evidence when complete paired cells were unavailable. Treated, test-enriched, reflex-tested, and verification-selected populations were retained with explicit restrictions on their use. Quantitative analysis of mixed dermatophytosis cohorts required separately extractable nail data. Non-English reports were assessed with language assistance when necessary.

**Table 2:** Inclusion and exclusion criteria and rules for quantitative eligibility. PAS: Periodic acid-Schiff. These criteria distinguish exclusion from the review from restricted use in narrative or paired-data synthesis.

Classification	Criterion	Operational rule
Inclusion	Population	Human clinical nail material from patients with suspected or confirmed onychomycosis.
Inclusion	Molecular index test	Molecular testing directly on clinical nail material, including PCR/qPCR, multiplex or nested PCR, arrays, sequencing, or isothermal amplification.
Inclusion	Conventional comparator	Culture, direct or fluorescence microscopy, PAS/histopathology, or a prespecified conventional composite on the same patient/sample or in an explicitly linked diagnostic algorithm.
Inclusion	Relevant diagnostic information	Data on diagnostic yield, paired disagreement, pathogen attribution, or clinical diagnostic utility; lack of a complete 2 × 2 table alone did not preclude narrative inclusion.
Inclusion	Publication date	No lower publication-date restriction; eligible literature was identified through the documented searches and update in Table 1.
Inclusion	Language	No language restriction; language assistance was used when required.
Exclusion	Secondary literature	Reviews, overviews, editorials, or symposium summaries without an extractable eligible primary dataset; retained only as background or citation-searching sources.
Exclusion	Case-only reports	Single case reports or small rare-pathogen case-only reports without an eligible diagnostic comparison.
Exclusion	Isolate-only or analytical validation	Pure-isolate characterization or laboratory validation without direct molecular testing in an eligible clinical nail cohort.
Exclusion	Nonhuman material	Animal or environmental studies without an eligible human clinical nail comparison.
Exclusion	No nail population	Skin/scalp-only studies without relevant nail data.
Exclusion	No eligible diagnostic comparison	Reports confined to resistance, virulence, treatment efficacy, or antifungal susceptibility without the required molecular-versus-conventional comparison.
Exclusion	Extraction optimization alone	DNA extraction-method optimization without an eligible clinical diagnostic comparison.
Analysis rule	Mixed skin-and-nail cohorts	Nail-specific paired data were required for quantitative analysis. Mixed-site reports with relevant nail information but no nail cross-tabulation could contribute narrative/supportive evidence.
Analysis rule	Paired-data eligibility	The same patient/sample had to have reconstructable molecular+/comparator+, molecular+/comparator-, molecular-/comparator+, and molecular-/comparator- cells.
Analysis rule	Treatment and selection strata	Treated, enriched, reflex-tested, verification-selected, and algorithmic cohorts were retained separately from untreated primary diagnostic cohorts.
Analysis rule	Duplicate or overlapping reports	Reports from the same cohort were linked; companion reports were not counted as independent datasets. Unresolved overlap was flagged.

Study Selection and Data Collection

One reviewer performed title/abstract screening, full-text assessment, data extraction, and the initial selection of comparisons. Screening and extraction were not independently duplicated. We recorded reasons for exclusion and extracted population characteristics, treatment history, sampling methods, molecular targets, comparators, adjudication procedures, funding, and possible cohort overlap. Stable report identifiers link the characteristics, paired data, and appraisal tables in Appendices 1-4.

For each comparison, we extracted molecular-positive/comparator-positive (a), molecular-positive/comparator-negative (b), molecular-negative/comparator-positive (c), and double-negative (d) observations. We checked that counts were nonnegative and summed to the paired denominator, N = a + b + c + d. Binary detection agreement was distinguished from agreement on species identity. Reconstructions, invalid results, and comparisons sharing specimens were recorded explicitly. When invalid results could not be separated, the reported table was retained with a qualification. A valid-result alternative was derived only where the source identified the required allocation (Appendices 2-4).

Methodological Appraisal

We assessed five domains: population selection, index interpretation, comparator interpretation, flow and pairing, and clinical applicability. Appendix 3 reports judgments and supporting reasons for all 97 included reports. The assessment used 96 existing full-text extraction records, 21 targeted source rechecks, and the newly retrieved Felek full text. It was not a new independent reassessment of every article. Where the available record did not establish recruitment, thresholds, blinding, or handling of invalid results, the relevant judgment was unclear. Comparator judgments addressed paired detection and, where relevant, incorporation or selective adjudication; culture was not assumed to be an error-free reference standard. This tailored post hoc assessment was not a validated QUADAS-2 application, and no overall score was calculated.

Descriptive Synthesis and Sensitivity Analyses

The net detection difference was 100 × (b - c)/N percentage points. Molecular-only and comparator-only detection were reported separately as 100b/N and 100c/N. Zero cells were retained without continuity correction. The discordance ratio b/c was reported as infinite when b > 0 and c = 0, and undefined when both were zero. Appendix 4 documents corrections to the original workbook labeling of gross molecular-only detection and its handling of zero ratios.

We summarized direction, median, IQR, and range without pooling correlated comparisons as independent observations. Quantiles used linear interpolation. The initial exploratory culture set contained 40 comparisons from 37 reports. Review of that selection showed inconsistent application of its labels to small samples, zero cells, and reconstructed tables. We retained the initial result for transparency and assessed it against an explicit broader selection rule. These analyses describe paired detection; they do not estimate sensitivity or specificity against a definitive disease standard. Direction counts were not treated as tests of statistical significance.

Post hoc analyses first restricted the initial culture set to one comparison per report. Selection favored the largest analyzable nail denominator, then the broadest eligible fungal target class, then the earliest stable row identifier. We applied the same hierarchy to a broader set of same-specimen clinical nail-culture comparisons, requiring corresponding target classes. Small samples, zero cells, and exact reconstruction did not themselves justify exclusion. We excluded test-positive enrichment, combined clinical and healthy-control denominators, reflex testing, treatment-monitoring cohorts, incompatible targets, anatomically mismatched specimens, identification-success-only endpoints, and aggregates of correlated targets. Separable clinical arms and standard assay thresholds were eligible. An available untreated arm was preferred to an enriched treated-nonresponder cohort; mixed or unreported prior treatment was retained and examined separately. Appendix 4 records each decision.

For analyses with one comparison per report, we also calculated a descriptive sample-size-weighted mean: 100 × Σ(b - c)/ΣN. Further analyses examined target class, directly tabulated versus reconstructed cells, treatment exposure, reported washout or untreated status, and identified consistency or flow concerns. To assess possible cohort overlap, we retained the larger eligible Dhib report and the largest eligible report from the possible Bako laboratory family; another analysis substituted the other Dhib report. These exclusions addressed uncertainty about overlap without assuming that patient identities were shared.

Individual net differences were accompanied by conservative 95% CIs obtained by subtracting opposite limits of separate equal-tailed 97.5% Clopper-Pearson intervals for b/N and c/N [7]. This construction provides at least 95% coverage under independent paired sampling and exact counts; it does not require independence between b and c. The intervals do not account for patient clustering, reconstruction error, bias, or multiple comparisons. We did not assign intervals to rows combining six correlated target determinations per nail or calculate a pooled interval or P value. Appendix 2 gives the formulas. Analyses used Python 3.12.14, NumPy 2.3.5, and SciPy 1.17.0.

Results

Study Selection

Of 102 reports sought, 101 were retrieved and assessed, four were excluded after full-text review, and 97 were included. PubMed contributed 77 included reports, Google Scholar nine, and citation searching 11. One citation-search report, CC060, could not be retrieved, so its eligibility remained unresolved. Figure 1 and Table 3 provide the source-specific counts. Appendix 5 records the full-text exclusions and the reassessment of two earlier scope decisions.

**Figure 1:**
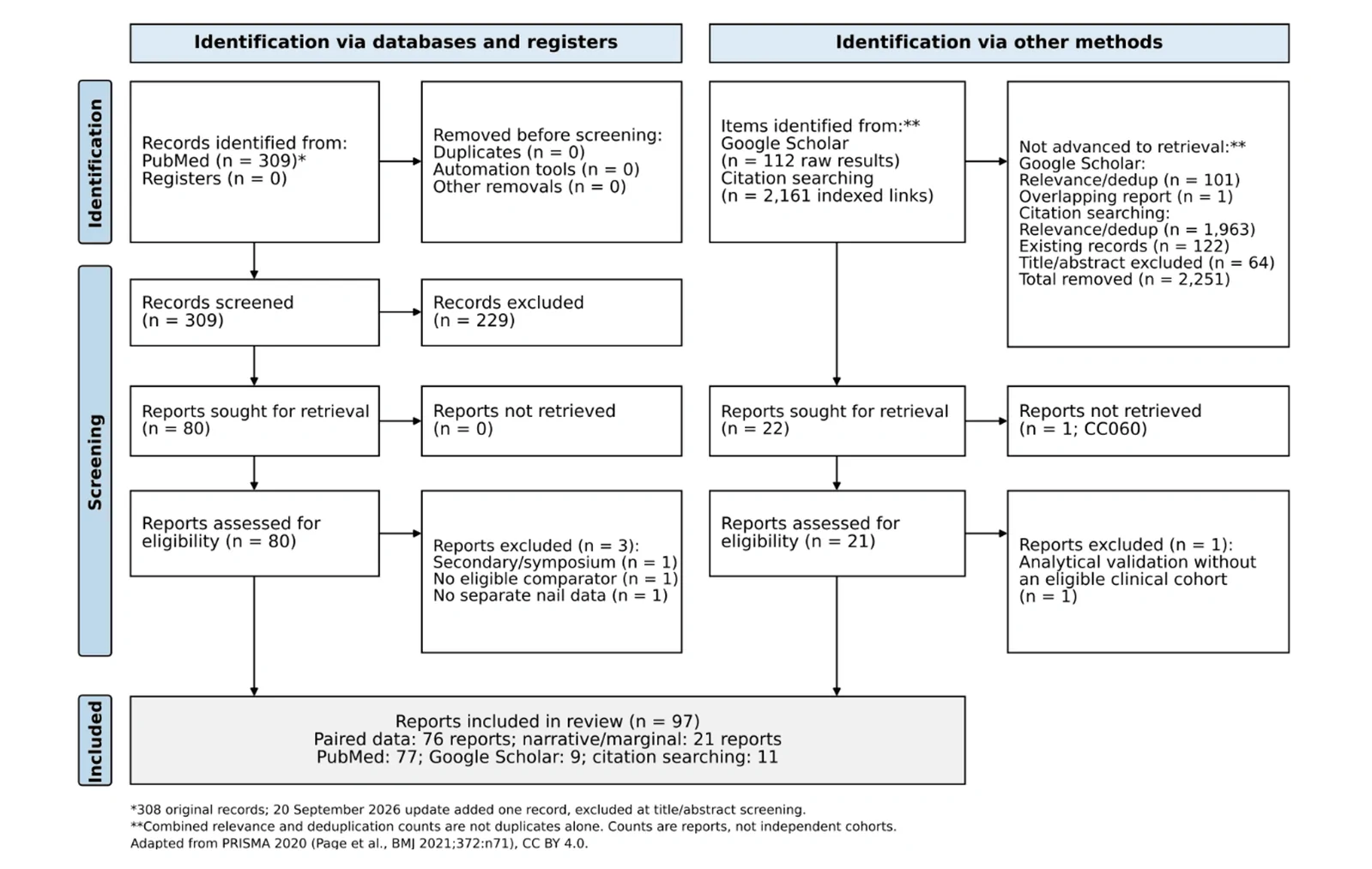
PRISMA 2020 flow diagram of study identification and selection. Adapted from the official template for new reviews using databases and other sources [6] (CC BY 4.0). Source-specific relevance filtering and deduplication are shown as combined removals rather than as duplicate counts alone. The September 20, 2026, update added one record, which was excluded at title/abstract screening. Counts represent reports and do not establish the independence of cohorts. PRISMA: Preferred Reporting Items for Systematic Reviews and Meta-Analyses.

**Table 3:** Final source-specific selection accounting.

Source	Recorded yield	Full texts assessed	Included	Excluded after full-text assessment
PubMed	309 records	80	77	3
Google Scholar	112 raw results	10	9	1
Citation chasing	2,161 indexed links	11	11	0
Overall	102 sought; 1 not retrieved	101	97	4

The four full-text exclusions comprised one secondary/symposium report, one report without an eligible comparator, one analytical validation study without an eligible clinical cohort, and one report without an eligible or separately extractable nail population. Appendix 5 provides the report identifiers and reasons.

One citation-search report, CC060, was sought but not retrieved. It is not included in the full-text-assessed or full-text-exclusion counts.

Study Characteristics and Appraisal

The 97 included reports were published between 1998 and 2026. Seventy-six supplied paired 2×2 data; 21 contributed marginal or narrative evidence. We extracted 174 comparisons: 97 culture-type, 43 microscopy-type, seven PAS/histopathology, 25 conventional or adjudicated composite, and two orthogonal molecular/adjudicative comparisons. Several reports contributed more than one assay, threshold, sampling, or treatment comparison. These totals therefore do not represent independent cohorts.

Populations included clinically suspected cases, routine laboratory submissions, previously positive samples, and treated or selectively verified patients. Molecular methods ranged from conventional, nested, real-time, and multiplex PCR to restriction fragment length polymorphism, microarrays, sequencing, and recombinase polymerase amplification. Assay coverage varied from a single species to broad fungal identification. Appendix 1 provides the characteristics and synthesis role of every included report.

Felek R et al. evaluated 46 suspected cases and tabulated 37 patients selected for positive culture and/or EUROArray Dermatomycosis (EUROIMMUN Medizinische Labordiagnostika AG, Lübeck, Germany) results; 21 had previous antifungal exposure. Their patient-level table yielded 15 double-positive and 22 molecular-positive/culture-negative specimens. Four endpoint-specific comparisons were reconstructed for this selected cohort; the other nine patients were not imputed. These comparisons were retained separately and excluded from the broader culture analysis because inclusion in the reported cohort depended on test positivity [8].

High concern was recorded for population selection in 25 reports, flow or pairing in 19, and comparator interpretation in eight. The comparator concerns generally applied to composite or adjudicated analyses rather than every raw comparison in those reports. Clinical applicability was restricted in 36 reports. Index interpretation was unclear for all 97: the assessment records did not establish both prospective thresholds and blinded interpretation. This finding reflects the limits of the available assessment, not proof that every study omitted those safeguards. Appendix 3 provides the individual judgments and their source basis. Table 4 summarizes the methodological domains and their interpretive boundaries.

**Table 4:** Methodological domains used to appraise bias and applicability.

Domain	What the judgment addresses	Interpretive boundary
Population selection	Recruitment, enrichment, prior test selection, and exclusions	High indicates a documented selection concern; unclear indicates insufficient documentation.
Index interpretation	Prospective threshold and interpretation without knowledge of comparator results	Unclear does not mean that blinding or prospective thresholds were absent.
Comparator interpretation	Independent detection endpoint, index inclusion in composites, and selective resolution	Low concern for raw detection does not make culture an error-free disease standard.
Flow and pairing	Valid same-specimen pairs, exclusions, timing, and denominator consistency	Unknown handling of inhibited or missing results is retained as a limitation.
Clinical applicability	Nail population, targets, treatment, setting, and clinical unit	Applicability concern is distinguished from bias in the reported counts.

This is a tailored post hoc appraisal of documented concerns, not a validated quality score. Appendix 3 contains all individual judgments, reasons, and evidence provenance. High, low, and unclear refer to each specified domain; no global rating was assigned.

Paired Detection Against Culture and Structural Tests

In the initial exploratory culture set, molecular-only positives outnumbered culture-only positives in 38 of 40 comparisons from 37 reports. The reverse occurred in two. The median net difference was 16.1 percentage points (IQR, 11.5-22.9; range, -7.6 to 57.9). These comparison-level results preceded the post hoc selection analyses below.

Among six exploratory microscopy or histopathology comparisons, structural methods yielded more additional positives in four and molecular testing in two. One histology comparison had unresolved allocation of inhibited PCR results. This small selected subset cannot establish how often either method will detect more cases in clinical practice. Table 5 presents representative paired findings; Appendices 1-2 provide the complete dataset. Within-study results also differed by sampling and comparator definition [9,10].

**Table 5:** Representative paired discordance patterns in nail studies. M+/C-: Molecular-positive/comparator-negative; M-/C+: Molecular-negative/comparator-positive; PAS: Periodic acid-Schiff; RFLP: Restriction fragment length polymorphism. Rows are representative and were not statistically pooled.

Study	Paired N	Molecular test/comparator	M+/C-	M-/C+	Interpretive value
Walberg M, et al. (2006) [11]	346	PCR/sequencing vs culture	67	15	Molecular-only detection exceeded culture-only detection.
Brillowska-Dabrowska A, et al. (2010) [12]	109	Multiplex PCR vs culture	18	3	Microscopy supported many molecular-only results; inhibition and splitting were documented.
Kondori N, et al. (2010) [13]	177	Commercial duplex PCR vs culture	21	3	Routine target-matched comparison; culture retained off-panel recovery.
Dhib I, et al. (2014) [14]	201	Multiplex PCR vs culture	63	0	No culture-only dermatophyte positives in this comparison; possible cohort overlap was assessed separately.
Debuysschere C, et al. (2023) [15]	131	Novaplex (Seegene Inc., Seoul, South Korea) real-time PCR vs culture	29	0	A second PCR confirmed 28/29 molecular-only specimens.
Guegan H, et al. (2026) [16]	316	DNA microarray vs culture	51	6	Prospective multicenter nail subgroup with selective adjudication.
Caldwell B, et al. (2020) [17]	203	Multiplex PCR vs PAS	9	41	PAS-positive/PCR-negative specimens outnumbered the reverse pattern.
Gustafson E, et al. (2019) [18]	2452	PCR vs histopathology	79	290	Structural fungal detection exceeded PCR.
Hayette MP, et al. (2019) [19]	138	DermaGenius (PathoNostics B.V., Maastricht, the Netherlands) PCR vs PAS	24	14	Source-reported PAS comparison; allocation of eight inhibited PCRs remains unresolved.
Gupta AK, et al. (2023) [20]	512	Any-fungal PCR vs histopathology	0	26	Histology added fungal-positive cases to a broad molecular endpoint.
Menotti J et al. (2004) [9]	48	PCR-RFLP vs microscopy/culture composite	1	2	Near balance with optimal sampling; sampling quality was tested separately.
Gupta AK et al. (2007) [10]	62	Combined Trichophyton rubrum PCR vs culture	32	3	Prior-culture-positive cohort with some previous treatment; restricted applicability.

Additional molecular detection in culture-negative nails occurred across several platforms [11-14,16,21-33]. Some studies investigated these discrepancies further. A second molecular assay was positive in 28 of 29 molecular-positive/culture-negative nails in one study [15], and histopathology supported additional molecular findings in retrospective series [18,34]. Clinical improvement was reported in another series, although follow-up was selective and anatomical sites were combined [35].

The dataset also included reverse discordance against PAS and target-specific NDM comparisons [17,19,20]. Studies with healthy controls, narrow assay panels, marked enrichment, or unresolved denominators were retained with the corresponding restrictions [36-40]. Treatment-monitoring and reflex-testing comparisons were recorded separately from initial diagnostic comparisons [41-45].

Sensitivity Analyses

Restricting the initial culture set to one comparison per report gave a median net difference of 16.0 percentage points (IQR, 11.9-22.1), with 35 positive and two negative differences among 37 reports. The broader post hoc analysis included 50 reports: 47 differences were positive, two negative, and one zero. Its median was 16.6 points (IQR, 10.4-28.1), and its sample-size-weighted mean was 16.1 points across 10,189 paired observations. Unique patient numbers could not be established across reports. Excluding potentially overlapping cohorts left 47 reports and a median of 16.2 points (IQR, 10.6-28.2). Medians were 13.8 points for directly tabulated cells and 19.5 points for reconstructed cells. These strata differed in their constituent studies, so the contrast cannot be attributed to reconstruction itself. Table 6 presents the sensitivity results.

**Table 6:** Original exploratory analysis and post hoc culture sensitivity analyses. All analyses except the original exploratory set were introduced post hoc. Subsets derive from the broader 50-report set unless otherwise specified. Weighting is descriptive and is not adjusted for bias or unknown cohort overlap; summed denominators do not represent unique patient counts. A smaller eligible denominator represents an alternative selection rule, not an analysis chosen for its effect. Appendix 4 and the analysis workbook list all member IDs. pp: Percentage points.

Analysis	Reports / rows	Positive / negative / zero	Median, pp (IQR)	N-weighted mean, pp
Original exploratory set	37 / 40	38 / 2 / 0	16.1 (11.5-22.9)	Not used
One per report from original set	37 / 37	35 / 2 / 0	16.0 (11.9-22.1)	15.3
Broader rule; one per report	50 / 50	47 / 2 / 1	16.6 (10.4-28.1)	16.1
Possible-overlap exclusion	47 / 47	45 / 1 / 1	16.2 (10.6-28.2)	16.8
Alternative Dhib report	47 / 47	45 / 1 / 1	16.2 (10.6-25.2)	16.6
Directly tabulated cells	25 / 25	22 / 2 / 1	13.8 (7.4-22.1)	14.4
Reconstructed cells	25 / 25	25 / 0 / 0	19.5 (15.4-30.0)	17.3
Exclude consistency concerns	46 / 46	43 / 2 / 1	16.2 (10.4-28.1)	15.9
Exclude four flagged treatment-exposure comparisons	46 / 46	43 / 2 / 1	16.2 (9.7-26.6)	15.5
Reported washout/untreated	7 / 7	6 / 0 / 1	14.2 (5.2-17.1)	11.3
Exclude preanalytic loss report	49 / 49	46 / 2 / 1	16.2 (10.2-28.1)	16
Exclude unresolved inhibition	49 / 49	46 / 2 / 1	16.2 (10.2-28.0)	15.3
Alternative smallest eligible N	50 / 50	47 / 2 / 1	18.5 (11.3-28.1)	17.7

Source checks identified several issues relevant to these analyses. Hayette MP et al. included first consultations without previous treatment, but eight of 138 PCR results were inhibited. Excluding the inhibited specimens gave 60 double-positive, 27 molecular-only, six culture-only, and 37 double-negative results (N = 130). This reconstruction, D089V, replaced the original row only in the broader analysis; allocation of inhibited results against histology and composite definitions remained unresolved [19]. Debuysschere C et al. analyzed 131 samples after excluding 189 for insufficient quantity or unsuitable transport [15]. Separate sensitivity analyses addressed that loss, possible Dhib cohort overlap, and unresolved inhibition in Kardjeva V et al. [14,21].

Discussion

Principal Findings

Molecular-only positivity exceeded culture-only positivity in most of the selected paired comparisons. The direction persisted when we limited the analysis to one comparison per report and excluded potentially overlapping cohorts. This supports caution in dismissing a molecular finding solely because culture is negative. It does not show how many additional findings represent infections requiring treatment. The contrasting observations against PAS and microscopy also show why a detection difference depends on the comparator. The observed medians describe the included comparisons and should not be presented as an expected improvement for an individual clinic or assay.

Biological Signals and Pathogen Attribution

Several mechanisms could explain the observed discordance (Table 7). Culture requires viable organisms and favorable growth conditions; previous treatment, low fungal burden, transport, or overgrowth may prevent recovery. PAS and microscopy can remain positive when fungal structures are present, while molecular assays may detect DNA after the viable burden has fallen. Culture may also recover organisms outside a molecular panel. The paired counts alone cannot determine which mechanism explains a particular discrepant specimen [1,3,4,12,41-43].

**Table 7:** Biological signals and principal interpretive implications of major diagnostic modalities. Author synthesis of the cited literature; each source is included in the reference list. The interpretive implications describe what a test result can support and do not establish a universal reference standard. PAS: Periodic acid-Schiff.

Method	Primary biological signal	Principal strength	Main discordance implication	Sources
Direct/fluorescence microscopy	Visible fungal elements	Rapid structural evidence; low cost	Positive microscopy with negative culture can reflect nonviable or nonculturable fungi; species is usually unknown.	[1,3,4]
PAS histopathology	Fungal elements in nail keratin	Sensitive structural evidence supporting fungal involvement	Structural detection does not establish species or viability; findings can differ from organism-targeted tests.	[1,4]
Fungal culture	Organism capable of growth	Broad/off-panel recovery; further work-up may be possible	A negative culture can reflect therapy, low burden, heterogeneity, or overgrowth; a positive mold may be a contaminant.	[1,3,4]
Targeted PCR/qPCR	DNA from prespecified targets	Fast, sensitive, and potentially species-level	DNA does not prove viability or invasion; a negative result may reflect an off-panel target or inhibition.	[3,4]
Array/sequencing	Broader molecular taxonomic signal	Wider species coverage and mixed-detection capability	Greater breadth increases the need for contamination control and causal-attribution thresholds.	[21,22,36]

Attribution is especially difficult for NDMs, yeasts, and mixed detections. Fungal DNA has been detected in healthy control nails, demonstrating that presence alone is insufficient to establish disease [37,46]. Histologic findings, repeat sampling, and confirmation by another method may support an etiologic role [20,36]. Their value depends on what they add: a second molecular assay may confirm identification while sharing contamination or sampling errors with the first. Corroboration should therefore be interpreted in relation to the organism and clinical findings.

A negative molecular result requires equal attention to assay coverage and specimen quality. Inhibition, inadequate material, extraction failure, or uneven fungal distribution can account for a positive structural or culture result despite negative PCR [9,12,19]. The site sampled and the way a specimen is divided can affect the comparison. A narrow panel also cannot exclude infection by an organism it was not designed to detect [36,40].

Treatment Exposure and Emerging Platforms

Previous antifungal treatment changes the meaning of a discordant result. Culture may become negative while DNA remains detectable from viable organisms, nonviable material, or both [10,41-43]. The monitoring studies in this review did not establish a PCR threshold that independently defines treatment failure. Persistent positivity should therefore be considered alongside the treatment timeline, clinical course, structural findings, and the properties of the assay.

Shorter turnaround and more detailed identification may be useful even when a higher detection rate cannot be interpreted as greater clinical accuracy. Arrays, multiplex PCR, isothermal amplification, and sequencing offer different combinations of speed and target coverage [16,22,29-31,33,44,46,47]. Broader coverage may identify an off-panel pathogen, but it also introduces additional findings whose clinical relevance must be assessed [36,39,46]. Molecular-first workflows with selective culture have been evaluated, although their performance and consequences for treatment require assessment in the intended clinical population [45].

Clinical Interpretation of Discordant Results

Table 8 organizes the main discordant patterns and proposes questions for clinical assessment. It has not been prospectively validated. For a clinically compatible dermatophyte PCR result with negative culture, structural evidence, repeat detection, or an additional method may strengthen attribution. An isolated yeast or NDM finding requires closer consideration of colonization and contamination. When structural testing is positive but PCR is negative, useful first checks include panel coverage, internal controls, and sampling. Repeat sampling, selective culture, or broader identification may then address the specific uncertainty [3,15,19,20,45].

**Table 8:** Clinical interpretation of common discordant patterns. Author synthesis of the cited evidence, not a prospectively validated decision algorithm. PAS: Periodic acid-Schiff; NDM: Non-dermatophyte mold.

Pattern	Plausible explanations	Proposed next step	Sources
PCR-positive/culture-negative	Low burden, therapy, growth failure, overgrowth, heterogeneity, residual DNA, colonization, or contamination	Review pathogen plausibility and structural test results. For yeasts/NDMs, consider PAS, repeat sampling, or orthogonal confirmation.	[4,15,18,20]
PCR-negative/culture-positive	Off-panel organism, inhibition, aliquot heterogeneity, target variation, or culture contamination	Check the internal control, panel coverage, and specimen quality. Consider repeat or broader testing; seek corroboration before attributing infection to an unusual mold.	[4,12,20,36]
PCR-positive/PAS- or microscopy-negative	Low burden, early or noninvasive colonization, residual DNA, or structural sampling miss	Do not equate DNA detection with invasion; integrate pathogen type, clinical pattern, repeat detection, and treatment state.	[4,20,37]
PCR-negative/PAS- or microscopy-positive	Off-panel fungus, inhibition, or a molecular aliquot lacking fungal material	Consider culture or broader identification. Review sampling and extraction before repeating targeted testing.	[19,20,44,45]
Persistent PCR-positive during therapy	Residual viable fungi below the culture threshold or persistent nonviable DNA	Do not define treatment failure by DNA detection alone; integrate the clinical trajectory and structural or viability evidence.	[41-43]

The way discordance is adjudicated can itself distort conclusions. A composite definition that includes the index test introduces incorporation bias, while testing only selected discrepancies can produce verification bias. Latent-class methods offer one approach to imperfect reference tests but depend on assumptions about test dependence and model structure; we did not apply them [47]. Additional molecular positivity, clinical improvement, and agreement with another assay should therefore be reported as distinct observations.

Limitations

The review has several limitations. Selection and extraction were performed by one reviewer, and the protocol was not prospectively registered. Our search centered on PubMed. Google Scholar results depend on the search interface and ranking, so screening all 112 returned results did not ensure exhaustive coverage. Citation searching also depended on the seed studies and available indexing, with OpenAlex substituted for blocked forward Scholar searches. The PubMed search was updated on September 20, 2026, but the supplementary searches retained their earlier dates. Relevant reports may have been missed, and one identified report, CC060, remained unavailable. Its eligibility and possible contribution could not be determined.

The evidence also varied substantially in population, sampling, treatment exposure, assay coverage, and comparator definition. Some cells required reconstruction, and several reports left denominators or invalid-result handling unresolved. Our post hoc appraisal relied largely on existing extraction records with targeted source checks; it was not an independently duplicated QUADAS-2 assessment. The inconsistent initial comparison labels prompted post hoc selection and sensitivity analyses. Their broadly similar results do not remove selection bias, uncertainty about cohort overlap, or the influence of large laboratory series on weighted summaries. Selecting one comparison per report reduces repeated counting within reports but cannot establish independence between reports. We did not formally grade certainty or assess reporting bias quantitatively, and the proposed clinical framework remains unvalidated.

Implications for Research

Prospective studies should report complete paired cross-tabulations from a defined clinical population, with treatment exposure, sampling, assay coverage, and invalid results documented. Adjudication should follow a prespecified procedure applied consistently to discordant specimens. For NDMs, yeasts, and monitoring during treatment, confirmation of organism identity should be separated from evidence of viable or invasive infection. Studies linking diagnostic strategies to treatment decisions and patient outcomes are needed to establish whether additional detection improves care.

## Conclusions

Molecular assays frequently detected fungal targets in nails with negative cultures, while microscopy and PAS sometimes identified fungal structures missed by molecular testing. The clinical significance of disagreement depends on assay coverage, specimen quality, treatment history, and the plausibility of the organism as a pathogen. The available paired evidence supports interpreting these methods together. It does not establish a universal increase in diagnostic accuracy or justify treatment decisions based on molecular positivity alone.

## Appendices

Appendix 1

Complete Report Characteristics

Table 9 identifies every included report and its contribution. Reports without nail-specific cross-tabulations are retained only for the narrative roles permitted by Table 2. Numbers denote the reported units, which may be specimens rather than individual patients. Report IDs preserve the historical register and do not necessarily reproduce the final bibliographic year.

**Table 9:** Characteristics and synthesis roles of all 97 included reports. OM: Onychomycosis; ITS: Internal Transcribed Spacer; ITS1: Internal Transcribed Spacer 1; ITS2: Internal Transcribed Spacer 2; KOH: Potassium Hydroxide; PAS: Periodic Acid-Schiff; GMS: Grocott Methenamine Silver; RFLP: Restriction Fragment Length Polymorphism; PCR-RFLP: Polymerase Chain Reaction-Restriction Fragment Length Polymorphism; NDM: Non-Dermatophyte Mold; RPA: Recombinase Polymerase Amplification; LFD: Lateral Flow Dipstick; RPA-LFD: Recombinase Polymerase Amplification-Lateral Flow Dipstick; LAMP: Loop-Mediated Isothermal Amplification; CHS1: Chitin Synthase 1; TEF1-alpha/TEF1-α: Translation Elongation Factor 1-alpha; CFW: Calcofluor White; OD: Optical Density; n: Number of specimens/participants/observations.

Report and source	Population and design	Nail material and treatment	Molecular method and comparator	Synthesis role and row IDs
OMD-2006-Walberg Walberg M et al. (2006) [11]	Clinically suspected onychomycosis; 10 healthy controls reported separately. Prospective diagnostic comparison Norway; tertiary dermatology/mycology	Nail material: 346. Unique patient count is not inferred from specimen totals. Not reported	18S rDNA PCR followed by sequencing; Targets: Broad fungal amplification; primary binary analysis restricted to dermatophyte detection; Comparator: Culture	Paired 2 × 2 D001 Candidate-complete paired culture comparison
OMD-2025-Ivagnes Ivagnes V et al. (2025) [48]	120 mixed dermatomycosis specimens: 78 nails, 35 skin scrapings, 7 other Prospective/observational pilot using residual clinical specimens Italy; tertiary hospital laboratory	Nail material: 78. Unique patient count is not inferred from specimen totals. Not reported	EUROArray Dermatomycosis microarray; Targets: Commercial multiplex panel; 56 fungal targets; Comparator: Microscopy and culture; panfungal PCR resolver	Narrative only Narrative only-non-nail-specific paired data
OMD-2024-Gupta-US Gupta AK et al. (2024) [49]	Abnormal-appearing toenail specimens submitted in 2022–2023. Retrospective laboratory database study United States; national commercial laboratory	Nail material: 96,293. Unique patient count is not inferred from specimen totals. Not reported	OIAD multiplex PCR; Targets: Dermatophytes, yeasts and NDM panel; Comparator: Histopathology	Paired 2 × 2 D002 Candidate-histopathology comparator; overlap check required
OMD-2023-Marin-Maldonado Marin-Maldonado F et al. (2023) [50]	242 PAS-submitted toenails; 237 valid qPCR; 151 high/moderate-quality Sanger sequences. Retrospective comparison on archived nail remnants Puerto Rico; pathology laboratory	Nail material: 242. Unique patient count is not inferred from specimen totals. Not reported	Onycho+ multiplex qPCR; Targets: *T. rubrum* plus pan-dermatophyte; Comparator: PAS histology; ITS2 Sanger sequencing	Paired 2 × 2 D003, D004 Separate/sensitivity analysis-verification-selected subset
OMD-2026-Dey Dey A et al. (2026) [32]	139 clinically suspected onychomycosis outpatients, July 2023–June 2024. Cross-sectional descriptive diagnostic study Bangladesh; Sir Salimullah Medical College, Dhaka	Nail material: 139. Unique patient count is not inferred from specimen totals. Not reported	Universal ITS1/ITS4 conventional PCR; Targets: Broad fungal ITS amplicon; species inferred from amplicon size; Comparator: Culture	Paired 2 × 2 D005 Candidate with high risk-of-bias caveat
OMD-2026-Villanueva-Reyes Villanueva-Reyes J et al. (2026) [46]	51 composite-confirmed onychomycosis cases and 53 healthy nails; molecular results in 103. Prospective case/control diagnostic comparison Colombia; Universidad del Valle	Nail material: 104. Unique patient count is not inferred from specimen totals. Not reported	ITS amplicon nanopore sequencing; Targets: Broad fungal sequencing; Comparator: Composite; Culture	Paired 2 × 2 D006, D007 Separate-case/control spectrum; attribution sensitivity analysis
OMD-2006-Yoshimura Yoshimura R et al. (2006) [51]	100 KOH-confirmed tinea unguium nail specimens. Comparative diagnostic study Japan; clinical mycology	Nail material: 100. Unique patient count is not inferred from specimen totals. Not reported	PCR-RFLP; Targets: Dermatophyte identification; Comparator: Fungal culture	Marginal yields only Marginal yields only-do not infer 2 × 2
OMD-2018-Bao Bao F et al. (2018) [52]	204 specimens from 187 patients with suspected onychomycosis. Prospective diagnostic comparison China; clinical dermatology/mycology	Nail material: 204. Unique patient count is not inferred from specimen totals. Excluded topical/systemic antifungal use within prior 2 weeks	ITS rDNA PCR followed by sequencing; Targets: Broad fungal species identification; Comparator: Culture	Paired 2 × 2 D008 Candidate-complete paired culture comparison
OMD-2019-Gustafson Gustafson E et al. (2019) [18]	425-sample PCR–culture cohort; separate sequential 2,452-sample PCR–culture–histology cohort Retrospective laboratory validation/database analysis United States; Bako Diagnostics	Nail material: 2452. Unique patient count is not inferred from specimen totals. Not reported	Real-time PCR screen plus reflex species panels; discordant 18S sequencing; Targets: Up to 15 fungi across dermatophyte, saprophyte/NDM and yeast classes; Comparator: Culture; Histopathology	Paired 2 × 2 D009, D010, D011 Candidate; choose one nonoverlapping cohort per comparator
OMD-2007-Garg Garg J et al. (2007) [53]	152 clinically suspected onychomycosis patients; 83 empirically treated nonresponders and 69 untreated. Prospective diagnostic comparison India; tertiary hospital	Nail material: 152. Unique patient count is not inferred from specimen totals. 83 received 2–3 months empirical antifungal therapy; analyze separately	Pan-dermatophyte nested PCR; Targets: Dermatophyte-specific; Comparator: Culture; KOH microscopy	Paired 2 × 2 D012, D013, D014, D015 Separate treated and untreated strata; no combined treatment-naive pool
OMD-2007-Brillowska-Dabrowska Brillowska-Dabrowska A et al. (2007) [23]	118 clinically diseased nails submitted for routine microscopy and culture. Routine-sample method evaluation Denmark; Statens Serum Institut	Nail material: 118. Unique patient count is not inferred from specimen totals. Not reported	Five-hour multiplex pan-dermatophyte/*T. rubrum* PCR; Targets: Dermatophytes plus specific *T. rubrum* call; Comparator: Culture	Paired 2 × 2 D016 Candidate-complete paired dermatophyte/culture
OMD-2006-Kardjeva Kardjeva V et al. (2006) [21]	195 direct nail specimens; separate 66 previously collected etiologic strains. Prospective/clinical method development with separate isolate arm Bulgaria/Greece with German molecular laboratory	Nail material: 195. Unique patient count is not inferred from specimen totals. Not reported	*T. rubrum* T1-PCR plus ITS PCR-RFLP/sequencing and SCOP-PCR; Targets: Broad workflow for dermatophytes and selected molds; Comparator: Culture; Microscopy	Paired 2 × 2 D017, D018 Candidate-direct nail cohort only
OMD-2026-Guegan Guegan H et al. (2026) [16]	487 valid samples from 407 patients; nail subgroup n=316. Prospective multicenter diagnostic evaluation France; prospective multicenter laboratories	Nail material: 316. Unique patient count is not inferred from specimen totals. Washout specified; treatment history reviewed only for discordant cases	DendrisKIT DP PCR plus DendrisCHIP microarray; Targets: Dermatophyte panel plus *C. albicans*; automated chip interpretation; Comparator: Culture; Composite	Paired 2 × 2 D019, D020 Candidate-nail subgroup; separate culture and composite comparators
OMD-2022-Pospischil Pospischil I et al. (2022) [44]	16,094 consecutive nail samples; PCR/sequencing applied to 1,148 inconclusive/inconsistent cases. Retrospective conditional/reflex cohort Switzerland/Austria; specialized dermatomycology laboratory	Nail material: 16,094. Unique patient count is not inferred from specimen totals. Not reported	Direct-nail 28S rDNA PCR plus sequencing; Targets: Broad fungal identification; Comparator: Culture	Paired 2 × 2 D021 Separate reflex/conditional analysis only
OMD-2012-Verrier Verrier J et al. (2012) [54]	679 abnormal nails; primary comparison in 624 microscopy-positive specimens; 20 healthy controls. Diagnostic method-development cohort Switzerland; CHUV Lausanne	Nail material: 679. Unique patient count is not inferred from specimen totals. Not reported; prior therapy discussed as culture-negative mechanism	28S PCR-terminal RFLP; sequencing for off-ladder peaks; Targets: Dermatophytes plus 12 NDF markers, expandable reference ladder; Comparator: Culture	Paired 2 × 2 D022 Separate microscopy-positive method-development cohort
OMD-2022-Bieber Bieber K et al. (2022) [29]	67 suspected OM cases plus 24 healthy nail controls; separate swab substudy. Prospective diagnostic comparison with healthy controls Germany; single-center prospective study	Nail material: 91. Unique patient count is not inferred from specimen totals. Not systematically reported	EUROArray Dermatomycosis DNA microarray; Targets: 56 fungal pathogens; Comparator: Culture; Microscopy; Composite	Paired 2 × 2 D023, D024, D025 Sensitivity analysis-reconstructed cells; composite circularity flagged
OMD-2024-Eijkenboom Eijkenboom QL et al. (2024) [55]	86 clinically suspected cases and 14 controls; analyzed composite-positive/negative groups. Prospective imaging-primary diagnostic study Germany; LMU Munich/Augsburg	Nail material: 100. Unique patient count is not inferred from specimen totals. Excluded topical/systemic antifungal therapy within 3 months	EUROArray Dermatomycosis PCR/microarray; Targets: Commercial fungal panel; Comparator: KOH, culture, limited histopathology; imaging tests	Narrative only Narrative only
OMD-2024-Katsiaunis Katsiaunis A et al. (2025) [34]	306 patients with both PCR and fungal culture. Retrospective patient-level comparison United States; academic center in New York City with external laboratory testing	Nail material: 306. Unique patient count is not inferred from specimen totals. Prior treatment variably present/not central; not used for primary stratification	Targeted PCR panel; Targets: Separate dermatophyte and NDM categories; Comparator: Culture	Paired 2 × 2 D026, D027 Candidate-separate dermatophyte and NDM analyses
OMD-2024-Harel Harel F et al. (2024) [30]	312 dermatophytosis specimens from 236 patients; nail subgroup 182 (161 toenails, 21 fingernails). Monocentric retrospective diagnostic evaluation Israel; tertiary dermatology/mycology laboratory	Nail material: 182. Unique patient count is not inferred from specimen totals. Not reported	Novaplex dermatophyte multiplex qPCR; Targets: *T. rubrum*, T*. mentagrophytes*/*interdigitale*/*benhamiae* complex, *T. tonsurans* and Microsporum; no NDM/yeast coverage; Comparator: Culture; Final diagnosis composite	Paired 2 × 2 D028, D029 Candidate-nail culture comparison; composite separate
OMD-2007-Savin Savin C et al. (2007) [56]	546 tested specimens: 236 regional proximal samples, 75 linked distal samples, 127 routine regional samples and 108 healthy controls. Prospective multicenter commercial-kit evaluation France; three clinical mycology laboratories	Nail material: 546. Unique patient count is not inferred from specimen totals. Recent antifungal exposure excluded by protocol	Onychodiag PCR–ELISA; Targets: Dermatophyte-specific PCR with ELISA signal; positive, gray-zone and negative thresholds; Comparator: Culture; Direct examination; Proximal culture; Proximal direct examination	Paired 2 × 2 D030, D031, D032, D033, D034, D035, D036, D037, D038 Separate strata and threshold sensitivity analyses
OMD-2024-Navarro-Perez Navarro-Pérez D et al. (2024) [31]	125 patients with clinically suspected toenail onychomycosis. Prospective comparative diagnostic-accuracy study Spain; dermatology clinic	Nail material: 125. Unique patient count is not inferred from specimen totals. Not reported	Direct-nail PCR; Targets: Fungal/dermatophyte molecular diagnosis as reported; Comparator: Culture	Paired 2 × 2 D039 Candidate-culture comparator
OMD-2024-Evrard Evrard S et al. (2024) [45]	106 nail samples from 102 patients with suspected onychomycosis. Retrospective/prospective laboratory strategy evaluation Belgium; university hospital	Nail material: 106. Unique patient count is not inferred from specimen totals. Not reported	DermaGenius multiplex PCR; Targets: Dermatophytes and selected fungi in a commercial multiplex panel; Comparator: Culture; Systematic composite	Paired 2 × 2 D040, D041, D042 Culture comparison candidate; composite/algorithm separate
OMD-2017-Iwanaga Iwanaga T et al. (2017) [43]	14 KOH-confirmed big-toenail tinea unguium patients treated with oral terbinafine. Prospective serial treatment-monitoring study Japan; dermatology/mycology	Nail material: 14. Unique patient count is not inferred from specimen totals. Terbinafine 125 mg daily; baseline, week 8 and week 16 analyzed separately	Quantitative PCR targeting dermatophyte actin DNA; Targets: Dermatophyte DNA and viability-related quantitative signal; Comparator: Culture; KOH microscopy	Paired 2 × 2 D043, D044, D045, D046, D047, D048 Separate treatment-monitoring analysis only
OMD-2018-Lubis Lubis NZ et al. (2018) [57]	35 patients with clinically suspected onychomycosis. Cross-sectional diagnostic comparison Indonesia; hospital dermatology clinic	Nail material: 35. Unique patient count is not inferred from specimen totals. Not reported	PCR-RFLP; Targets: Dermatophyte/fungal identification; Comparator: Culture	Paired 2 × 2 D049 Sensitivity/small-study candidate
OMD-2014-Han Han HW et al. (2014) [58]	32 nail specimens from 29 patients with suspected onychomycosis; 10 healthy nails separately. Prospective small method-evaluation study Taiwan; medical center	Nail material: 32. Unique patient count is not inferred from specimen totals. Not reported	PCR oligonucleotide array; Targets: Dermatophytes and *Candida albican*s; Comparator: Culture; Adjudicated composite	Paired 2 × 2 D050, D051 Raw culture comparison candidate; composite sensitivity analysis
OMD-2026-Wu Wu R et al. (2026) [47]	81 clinical nail specimens with suspected onychomycosis. Prospective three-test diagnostic study with Bayesian latent-class model China; hospital dermatology/mycology	Nail material: 81. Unique patient count is not inferred from specimen totals. Not reported	Multi-tube recombinase polymerase amplification; Targets: Panfungal plus *T. rubrum*, *T. interdigitale*, pan-Candida/*C. albicans*, pan-Trichosporon and pan-Aspergillus; Comparator: Culture; Microscopy	Paired 2 × 2 D052, D053 Candidate; RPA class sensitivity analysis
OMD-2021-Kabtani Kabtani J et al. (2021) [59]	105 dermatophytosis specimens; nail subgroup n=19. Prospective mixed-site diagnostic comparison Senegal; Dakar dermatology/mycology	Nail material: 19. Unique patient count is not inferred from specimen totals. Not reported	In-house real-time PCR and Eurobio commercial kit; Targets: Dermatophyte detection; Comparator: Conventional diagnosis	Paired 2 × 2 D054, D055 Separate nail subgroup/sensitivity
OMD-2010-Sato Sato T et al. (2010) [22]	106 microscopy-confirmed onychomycosis nail specimens; patient count internally reported as 83 versus 86 Diagnostic method-development study Japan; Keio University and Canon	Nail material: 106. Unique patient count is not inferred from specimen totals. Not reported	PCR-based DNA microarray; Targets: Broad human-pathogenic fungal panel plus universal fungal signal; Comparator: Culture	Paired 2 × 2 D056, D057 Separate microscopy-positive method-development analysis
OMD-2023-Gupta-NDM Gupta AK et al. (2023) [20]	512 toenail specimens/patients evaluated for onychomycosis and NDM. Retrospective single-point nail diagnostic comparison United States; national podiatric pathology laboratory	Nail material: 512. Unique patient count is not inferred from specimen totals. Not reported	OIAD multiplex PCR; Targets: Dermatophytes, yeasts and seven NDM genera; separate bacterial Pseudomonas output; Comparator: Histopathology; Culture	Paired 2 × 2 D058, D059 Candidate-comparator/endpoint strata separate
OMD-2025-Santosh Santosh AC et al. (2025) [60]	Nine bags of communal ground toenail dust representing approximately 400–500 podiatric patients. Pooled-sample molecular method study Australia; podiatry laboratory	Nail material: not reported. Unique patient count is not inferred from specimen totals. Unknown	Species-specific and nested PCR; Targets: *T. rubrum* and *T. interdigitale*; Comparator: Historical culture of pooled dust	Narrative only Narrative only
OMD-2020-Pakshir Pakshir K et al. (2020) [61]	137 suspected onychomycosis nail specimens plus 10 healthy controls. Cross-sectional diagnostic comparison Iran; Shiraz medical centers	Nail material: 137. Unique patient count is not inferred from specimen totals. Not reported	TEF1-alpha PCR; Targets: Candida species except reduced coverage for *C. glabrata*; Comparator: Culture	Paired 2 × 2 D060 Candidate-Candida-specific culture comparison
OMD-2022-Trovato Trovato L et al. (2022) [41]	109 toenail specimens: 52 untreated and 57 after empirical antifungal therapy. Prospective diagnostic comparison stratified by empirical treatment Italy; hospital infectious diseases/dermatology	Nail material: 109. Unique patient count is not inferred from specimen totals. 57 previously treated empirically; 52 untreated	Real-time multiplex PCR; Targets: Dermatophyte detection; Comparator: Microscopy; Culture; Conventional composite	Paired 2 × 2 D061, D062, D063, D064, D065 Treatment-stratified separate analysis
OMD-2017-Vrioni Vrioni G et al. (2017) [62]	252 separate patients with suspected onychomycosis. Prospective diagnostic comparison Greece; two Athens clinical microbiology laboratories	Nail material: 252. Unique patient count is not inferred from specimen totals. Prior antifungal treatment was not excluded; 12 PCR-positive specimens were from previously treated patients	Dermatophyte PCR kit multiplex PCR; Targets: Pan-dermatophyte CHS1 plus *T. rubrum* ITS2; Comparator: Culture; Conventional composite	Paired 2 × 2 D066, D067 Candidate-culture main, composite separate
OMD-2026-Gellrich Gellrich S et al. (2026) [35]	99 outpatients; 101 analyzed skin/nail samples. Retrospective consecutive routine comparison Germany; Berlin routine outpatient laboratory	Nail material: 52. Unique patient count is not inferred from specimen totals. Treatment status not reported at baseline; follow-up after antifungal therapy available for 19 array+/culture- patients across sites	EUROArray Dermatomycosis PCR microarray; Targets: 23 dermatophytes, three yeasts and three molds; Comparator: Culture	Paired 2 × 2 D068 Sensitivity-nail culture comparison
OMD-2025-Steixner Steixner S et al. (2025) [63]	4,483 skin, hair and nail specimens submitted 2018–2024. Retrospective PCR-first diagnostic workflow Austria; Medical University of Innsbruck routine laboratory	Nail material: 2,728. Unique patient count is not inferred from specimen totals. Not reported	In-house pan-dermatophyte qPCR followed by sequencing; Targets: CHS1 pan-dermatophyte detection with multilocus sequencing; Comparator: Conditional CFW microscopy and culture	Narrative only Narrative only-algorithm/workload
OMD-2026-Lotsch Lötsch F et al. (2026) [36]	124 clinical samples with suspected dermatophytosis Mixed-site clinical assay comparison plus analytical reference strains Austria; Medical University of Vienna clinical microbiology	Nail material: 59. Unique patient count is not inferred from specimen totals. Not reported	DermaGenius 2.0/3.0 multiplex real-time PCR and EUROArray Dermatomycosis; Targets: Dermatophyte-focused and broader fungal panels; Comparator: Fungal culture	Narrative only Narrative only-mixed-site technology comparison
OMD-2025-Muranen Muranen P et al. (2025) [64]	817 microscopy-positive nails with culture negative for dermatophytes or inconclusive. Retrospective conference-poster analysis Finland/Germany/Switzerland/Austria; Lausanne clinical samples with commercial collaborators	Nail material: 817. Unique patient count is not inferred from specimen totals. Not reported	D2 LSU PCR/sequencing and EUROArray Dermatomycosis; Targets: Broad fungal sequencing and commercial microarray; Comparator: Fungal culture categories	Narrative only Narrative only-gray-literature sensitivity evidence
OMD-2023-Guschin Guschin AE et al. (2023) [65]	258 patients: 154 skin, 62 hair and 42 nail specimens. Prospective/routine simultaneous diagnostic comparison Russia; Moscow dermatovenereology center	Nail material: 42. Unique patient count is not inferred from specimen totals. Not reported	AmpliPrime Dermatophytes multiplex RT-PCR; Targets: *T. rubrum*, *T. mentagrophytes* complex, *T. tonsurans*, *M. canis* and *E. floccosum*; Comparator: Culture; KOH microscopy	Paired 2 × 2 D069, D070 Candidate-small nail subgroup, comparator-separated
OMD-2022-Makled Makled AF et al. (2022) [66]	100 clinically diagnosed onychomycosis patients. Cross-sectional complete diagnostic comparison Egypt; Menoufia University dermatology/microbiology	Nail material: 100. Unique patient count is not inferred from specimen totals. Not clearly reported	Universal ITS PCR; Sanger sequencing of selected products; Targets: Broad fungal ITS detection; species identification on 30 products; Comparator: Culture	Paired 2 × 2 D071 Candidate-culture comparison
OMD-2019-Camacaro Camacaro L et al. (2019) [67]	49 patients with clinical tinea unguium plus 10 healthy controls. Small prospective clinical comparison Venezuela; Caracas mycology clinic	Nail material: 49. Unique patient count is not inferred from specimen totals. 51% stopped topical/systemic antifungals one month before evaluation; 42.9% reported no use; 6.1% unclear	In-house multiplex PCR; Targets: Pan-dermatophyte CHS1 plus *T. rubrum* and *T. mentagrophytes* ITS targets; Comparator: Culture	Paired 2 × 2 D072 Sensitivity-reconstructed culture comparison
OMD-2013-Chandran Chandran NS et al. (2013) [27]	99 patients with clinically suspected OM; 107 microscopy-positive nail specimens. Prospective longitudinal diagnostic comparison Singapore; National Skin Centre tertiary dermatology service	Nail material: 107. Unique patient count is not inferred from specimen totals. Not reported	Dermatophyte PCR Kit conventional multiplex PCR; Targets: *T. rubrum* plus pan-dermatophyte; Comparator: Culture; Direct microscopy	Paired 2 × 2 D073, D074, D075 Candidate-target culture main; other endpoints separate
OMD-2022-Gupta-Therapy Gupta AK et al. (2022) [42]	28 culture-confirmed participants completing topical efinaconazole follow-up to month 24. Prospective serial treatment-monitoring subset Canada; Mediprobe treatment study	Nail material: 28. Unique patient count is not inferred from specimen totals. Daily topical efinaconazole; serial sampling through month 24	Targeted fungal PCR panel; Targets: *T. rubrum*, *T. mentagrophytes* complex and selected NDMs; Comparator: Culture; KOH/culture composite	Paired 2 × 2 D080, D081, D082 Separate-treatment monitoring only
OMD-2014-Dhib Dhib I et al. (2014) [14]	201 suspected onychomycosis toenail specimens. Clinical method-development comparison plus analytical isolates Tunisia; clinical mycology laboratory	Nail material: 201. Unique patient count is not inferred from specimen totals. Not reported	In-house multiplex PCR; Targets: Pan-dermatophyte plus *T. rubrum* and *T. mentagrophytes*/*interdigitale* targets; Comparator: Culture; Direct microscopy	Paired 2 × 2 D083, D084 Sensitivity-culture main; microscopy separate
OMD-2020-Caldwell Caldwell B et al. (2020) [17]	209 clinically suspected hallux OM patients; 203 with adequate paired samples. Prospective paired clinical comparison USA; Cleveland podiatry clinic	Nail material: 203. Unique patient count is not inferred from specimen totals. No oral or topical antifungal use within the preceding year	PathoGenius multiplex PCR; Targets: Dermatophytes, selected molds and *C. albicans*; Comparator: PAS histopathology	Paired 2 × 2 D087 Main candidate-histopathology comparator
OMD-2023-Debuysschere Debuysschere C et al. (2023) [15]	131 consecutive nail samples submitted for suspected OM. Prospective complete paired diagnostic study Belgium; Brussels hospital laboratory	Nail material: 131. Unique patient count is not inferred from specimen totals. Not reported	Novaplex multiplex RT-PCR; DermaGenius resolver; Targets: Dermatophyte complexes/species; Comparator: Culture	Paired 2 × 2 D093 Main candidate-culture comparison
OMD-2017-Hafirassou Hafirassou AZ et al. (2017) [37]	70 clinical OM nail samples and 15 healthy volunteer nails. Clinical case/control multi-assay evaluation Algeria/Spain; Constantine Hospital samples with reference-laboratory molecular testing	Nail material: 85. Unique patient count is not inferred from specimen totals. Not reported	Panfungal, pandermatophyte, Candida and Aspergillus RT-PCR assays; sequencing confirmation; Targets: Broad fungal, dermatophyte and selected yeast/mold targets; Comparator: Culture; Direct microscopy	Paired 2 × 2 D076, D077, D078, D079 Comparator- and endpoint-separated sensitivity analyses
OMD-2016-Gong Gong J et al. (2016) [26]	165 nails: 70 with suspected mycosis and 95 without obvious symptoms. Case/control-spectrum method evaluation with latent-class analysis China; Beijing clinical mycology setting	Nail material: 165. Unique patient count is not inferred from specimen totals. Not reported	Novel TaqMan real-time PCR; Targets: Pan-dermatophyte and *T. rubrum*; Comparator: Culture; Direct microscopy	Paired 2 × 2 D085, D086 Sensitivity-case/control spectrum, comparator-separated
OMD-2019-Hayette Hayette MP et al. (2019) [19]	138 archived finger/toenail clippings. Retrospective archived-specimen clinical evaluation Belgium; Liège university hospital laboratory	Nail material: 138. Unique patient count is not inferred from specimen totals. No previous treatment; first consultation (source rechecked)	DermaGenius Nail real-time multiplex PCR; Targets: Dermatophytes and *C. albicans*; Comparator: PAS histology; Culture; Conventional composite	Paired 2 × 2 D088, D089, D090 Main candidate-comparators separate
OMD-2011-Berk Berk E et al. (2011) [68]	90 patients with suspected OM and 20 healthy volunteers. Clinical case/control target-specific evaluation Turkey; hospital dermatology clinic	Nail material: 110. Unique patient count is not inferred from specimen totals. Not reported	TaqMan real-time PCR; Targets: *T. rubrum*-specific; Comparator: Direct microscopy; Culture	Paired 2 × 2 D091, D092 Sensitivity-target-specific culture and microscopy separate
OMD-2026-Khoo Khoo E et al. (2026) [69]	61 analyzable superficial fungal infection specimens: 50 nails, 10 skin, 1 hair. Prospective mixed-site microscopy-positive comparison Singapore; National Skin Centre	Nail material: 50. Unique patient count is not inferred from specimen totals. Not reported	EUROArray Dermatomycosis PCR microarray; Targets: Dermatophytes, selected yeasts and molds; Comparator: Fungal culture; microscopy by entry	Marginal yields only Narrative/marginal yield only
OMD-2026-Caza Caza M et al. (2026) [33]	149 residual nail samples submitted for KOH and/or fungal culture: 65 toenails, 4 fingernails and 80 unspecified nails. Retrospective laboratory-based evaluation of residual clinical nail samples plus separate analytical nail model Canada; Interior Health, LifeLabs and collaborating reference laboratories	Nail material: 149. Unique patient count is not inferred from specimen totals. Not reported for clinical samples; topical-product interference assessed only in contrived nails	Seegene Novaplex Dermatophyte multiplex real-time PCR after 30-minute lysis and automated STARlet extraction/setup; Targets: *T. rubrum* complex, *T. mentagrophytes* complex, *T. tonsurans*, *Microsporum* spp., *E. floccosum* and *C. albicans*; Comparator: Culture; Conventional composite; Molecular consensus	Paired 2 × 2 D102, D103, D104, D105, D106 Main candidate-specimen-level culture; composite separate; target-level rows supportive and not pooled
OMD-2010-Brillowska-Routine Brillowska-Dabrowska A et al. (2010) [12]	109 nail specimens submitted for suspected tinea unguium. Routine-laboratory complete paired evaluation Denmark; routine reference PCR laboratory	Nail material: 109. Unique patient count is not inferred from specimen totals. Not reported	Optimized 5-hour multiplex PCR with internal process control; Targets: Pan-dermatophyte CHS1 plus *T. rubrum* ITS2; Comparator: Culture; Direct microscopy; Conventional composite	Paired 2 × 2 D094, D095, D096 Main candidate-culture; microscopy/composite separate
OMD-2010-Kondori Kondori N et al. (2010) [13]	177 nail specimens with suspected onychomycosis. Prospective/routine commercial-kit comparison Sweden; Gothenburg clinical laboratory	Nail material: 177. Unique patient count is not inferred from specimen totals. Not reported	Dermatophyte-PCR kit duplex PCR; Targets: Pan-dermatophyte plus *T. rubrum*; Comparator: Culture; Fluorescence microscopy	Paired 2 × 2 D097, D098 Main candidate-culture; microscopy separate
OMD-2014-Dubljanin Dubljanin E et al. (2014) [24]	183 patients with suspected toenail onychomycosis. Prospective complete paired clinical cohort Serbia; University of Belgrade clinical cohort with SSI molecular collaboration	Nail material: 183. Unique patient count is not inferred from specimen totals. Topical/systemic antifungals excluded within prior 6 months	In-house pandermatophyte and *T. rubrum* PCR with *T. interdigitale* reflex PCR; Targets: Dermatophytes with *T. rubrum*/*T. interdigitale* differentiation; Comparator: Culture; Microscopy; Conventional composite	Paired 2 × 2 D099, D100, D101 Main candidate-culture; microscopy/composite separate
OMD-2025-Tirard-Collet Tirard-Collet P et al. (2025) [39]	85 clinical samples: 50 skin scrapings, stated 29 nails and 6 hair samples; Table 1 accounts for 52 skin, 27 nail and 6 hair samples. Retrospective mixed-site clinical comparison with selective sequencing France; Hospices Civils de Lyon routine mycology laboratory	Nail material: 29 stated; 27 tabulated. Unique patient count is not inferred from specimen totals. Not reported	DendrisKIT DP pan-fungal PCR, DNA chip and machine-learning identification algorithm; Targets: *C. albicans *and 12 dermatophyte targets; Comparator: Culture	Paired 2 × 2 D107 Support only-do not pool because of denominator inconsistency
OMD-2025-Kharibam Kharibam SD et al. (2025) [40]	350 clinically suspected patients screened; 100 KOH-positive, of whom 70 were classified as dermatophyte-positive and entered PCR comparison. Prospective diagnostic comparison India; tertiary care hospital in Central India	Nail material: 350. Unique patient count is not inferred from specimen totals. Patients reporting antifungal medication were excluded	In-house multiplex conventional PCR; Targets: *T. rubrum* and *T. mentagrophytes* only; Comparator: Culture	Paired 2 × 2 D108 Separate sensitivity stratum-do not pool with unselected cohorts
OMD-2020-Gill Gill PK et al. (2020) [38]	48 patients with dystrophic fungal nail infection. Small clinical diagnostic comparison India; tertiary care hospital	Nail material: 48. Unique patient count is not inferred from specimen totals. Not reported	Arbitrarily primed PCR using four random primers; Targets: Band-pattern identification of *T. rubrum* and *T. mentagrophytes*; non-dermatophytes off-target; Comparator: Culture	Paired 2 × 2 D109 Sensitivity only-zero-cell enriched cohort
OMD-2008-Gupta-Treated Gupta AK et al. (2008) [70]	83 patients with onychomycosis receiving antifungal therapy Comparative clinical study Canada; dermatology	Nail material: 83. Unique patient count is not inferred from specimen totals. All receiving antifungal therapy	Double-round conventional PCR; Targets: *T. rubrum* actin gene; Comparator: Calcofluor white microscopy; Culture	Paired 2 × 2 D110, D111 Separate treated stratum
OMD-2014-Iglesias-Sanchez Sánchez MJ et al. (2014) [71]	232 suspected nail fungal infections; 225 had sufficient material for PCR. Clinical paired diagnostic comparison Spain; three clinical centers in Cáceres province	Nail material: 232. Unique patient count is not inferred from specimen totals. Not reported	Nested conventional PCR; Targets: Pan-dermatophyte CHS1 plus *T. rubrum*-specific target; Comparator: Culture	Paired 2 × 2 D112 Candidate-complete paired culture comparison
OMD-2015-Spiliopoulou Spiliopoulou A et al. (2015) [72]	418 nail scrapings from dermatology outpatients Routine clinical paired comparison Greece; dermatology outpatient laboratory	Nail material: 418. Unique patient count is not inferred from specimen totals. Not reported	Commercial multiplex PCR; Targets: Pan-dermatophyte with *T. rubrum* identification; Comparator: Culture; Direct microscopy	Paired 2 × 2 D113, D114 Candidate with comparator-specific strata
OMD-2023-Navarro-Perez-Culture Navarro-Pérez D et al. (2023) [73]	121 patients with suspected onychomycosis and both culture and PCR Retrospective descriptive paired comparison Spain; specialized onychomycosis clinic	Nail material: 121. Unique patient count is not inferred from specimen totals. Prior treatment collected but not used as a paired stratum	Dermatophyte PCR; Targets: Dermatophyte positive/negative report; culture broader; Comparator: Culture	Paired 2 × 2 D115 Candidate with target-mismatch flag
OMD-2020-Mohtady Mohtady Ali H et al. (2020) [74]	61 patients with suspected onychomycosis. Clinical paired diagnostic comparison Egypt; university clinical cohort	Nail material: 61. Unique patient count is not inferred from specimen totals. Not reported	Conventional PCR; Targets: Dermatophyte detection; Comparator: Culture	Paired 2 × 2 D116 Separate sensitivity stratum; zero reverse-discordant cell
OMD-2017-Motamedi Motamedi M et al. (2017) [75]	853 suspected dermatophyte lesions; 538 nail specimens; culture available for a subset of the overall cohort. Clinical mixed-site comparison Iran; multicenter/university diagnostic cohort	Nail material: 538. Unique patient count is not inferred from specimen totals. Not reported	TaqMan real-time PCR; Targets: Pan-dermatophyte beta-tubulin; Comparator: Direct microscopy and culture	Narrative only Narrative/supportive only
OMD-2016-Mirhendi Mirhendi H et al. (2016) [76]	234 clinical specimens selected as positive by microscopy and/or culture across skin, nail and hair, including 80 nail specimens; 32 healthy nail controls. Analytical development plus verification-enriched clinical validation Iran/Japan; assay development and clinical validation	Nail material: 80 clinical + 32 healthy controls. Unique patient count is not inferred from specimen totals. Not reported	TaqMan real-time PCR; Targets: Pan-dermatophyte beta-tubulin; Comparator: Direct microscopy and culture	Narrative only Narrative/supportive only
OMD-2016-Emam Emam SM et al. (2016) [77]	80 patients with clinically suspected dermatophyte onychomycosis. Prospective diagnostic comparison Egypt; dermatology/mycology clinical cohort	Nail material: 80. Unique patient count is not inferred from specimen totals. Not reported	Nested PCR and real-time PCR; Targets: Dermatophyte detection/species targets; Comparator: Culture	Paired 2 × 2 D117, D118 Separate reconstructed strata
OMD-2012-Moallemzadeh Moallemzadeh SA et al. (2012) [78]	71 nail samples from patients with suspected tinea unguium. Clinical diagnostic comparison Iran; clinical nail cohort	Nail material: 71. Unique patient count is not inferred from specimen totals. Not reported	Conventional universal fungal PCR and *T. rubrum*-specific PCR; Targets: Universal fungal and *T. rubrum*-specific endpoints; Comparator: Direct examination and fungal culture	Narrative only Narrative/supportive only
OMD-2011-Li Li XF et al. (2011) [25]	Nail subgroup information: n=104. Clinical nail comparison with circular composite reference China	Nail material: 104. Unique patient count is not inferred from specimen totals. Not clearly reported or mixed	Triplex conventional PCR; Targets: Dermatophytes, yeasts and molds; Comparator: Culture; Direct microscopy	Paired 2 × 2 D119, D120 Comparator- and stratum-specific discordance analysis
OMD-2004-Arca Arca E et al. (2004) [79]	Nail subgroup information: n=52. Clinical nail comparison Turkey	Nail material: 52. Unique patient count is not inferred from specimen totals. Not clearly reported or mixed	Conventional fungal PCR; Targets: Fungal detection; Comparator: Culture; Direct microscopy	Paired 2 × 2 D121, D122 Comparator- and stratum-specific discordance analysis
OMD-2011-Beifuss Beifuss B et al. (2011) [80]	Nail strata reported, but no nail-specific paired cross-table. Multicenter mixed-site assay evaluation Germany; three centers	Nail material: 184. Unique patient count is not inferred from specimen totals. Not clearly reported or mixed	PCR-ELISA; Targets: Five dermatophyte species; Comparator: Direct microscopy and/or fungal culture.	Narrative only Narrative/supportive only
OMD-2009-Uchida Uchida T et al. (2009) [81]	Nail subgroup information: 80 nails; paired marginals only. Mixed-site clinical comparison Japan	Nail material: 80. Unique patient count is not inferred from specimen totals. Not clearly reported or mixed	Direct PCR; Targets: Dermatophytes with species differentiation; Comparator: Direct microscopy and/or fungal culture.	Narrative only Narrative/supportive only
OMD-2022-Trave Trave I et al. (2022) [82]	Nail subgroup information: n=100. Prospective real-life clinical nail comparison Italy; Genoa	Nail material: 100. Unique patient count is not inferred from specimen totals. Not clearly reported or mixed	EUROArray Dermatomycosis microarray; Targets: Dermatophytes and selected fungi; Comparator: Culture; KOH microscopy	Paired 2 × 2 D123, D124 Comparator- and stratum-specific discordance analysis
OMD-2017-Gupta-Nakrieko Gupta AK et al. (2017) [83]	Nail subgroup information: 167 single samples plus 43 serial-sample patient sets. Retrospective single and serial nail-sample agreement study Canada; reference laboratory	Nail material: 167. Unique patient count is not inferred from specimen totals. Not clearly reported or mixed	PCR with RFLP/species reporting; Targets: 16 dermatophytes and five non-dermatophyte molds; Comparator: Direct microscopy and/or fungal culture.	Narrative only Narrative/supportive only
OMD-2019-Watanabe-LAMP Watanabe S et al. (2019) [84]	Nail subgroup information: n=90. Microscopy-positive verification-selected nail cohort Japan	Nail material: 90. Unique patient count is not inferred from specimen totals. Not clearly reported or mixed	LAMP; Targets: *T. rubrum* and *T. interdigitale*; Comparator: Culture; Direct microscopy	Paired 2 × 2 D125, D126 Comparator- and stratum-specific discordance analysis
OMD-2016-Petinataud Petinataud D et al. (2016) [85]	Nail subgroup information: 90 patients; 180 nail samples. Within-patient sampling-quality comparison France; Nancy	Nail material: 180. Unique patient count is not inferred from specimen totals. Not clearly reported or mixed	Bio Evolution real-time PCR kit; Targets: Dermatophytes; Comparator: Culture; Direct examination	Paired 2 × 2 D127, D128, D129, D130 Comparator- and stratum-specific discordance analysis
OMD-2013-Paugam Paugam A et al. (2013) [86]	Nail subgroup information: 54 nails, two PCR uninterpretable; metrics only. Mixed-site clinical comparison France; Paris/Marseille	Nail material: 54. Unique patient count is not inferred from specimen totals. Not clearly reported or mixed	Real-time PCR; Targets: Dermatophytes; Comparator: Direct microscopy and/or fungal culture.	Narrative only Narrative/supportive only
OMD-2013-Winter Winter I et al. (2013) [87]	Nail subgroup information: 218 nail specimens; marginals only Prospective paired skin-and-nail cohort Germany	Nail material: 218. Unique patient count is not inferred from specimen totals. Not clearly reported or mixed	Topoisomerase-II PCR-ELISA; Targets: *T. rubrum*, *T. interdigitale*, *E. floccosum*; Comparator: Direct microscopy and/or fungal culture.	Narrative only Narrative/supportive only
OMD-2006-Monod Monod M et al. (2006) [88]	Nail subgroup information: 111 selected microscopy-positive nails; 6,848-sample descriptive denominator Routine laboratory series with selected molecular validation Switzerland; Lausanne	Nail material: 111. Unique patient count is not inferred from specimen totals. Not clearly reported or mixed	28S PCR/sequencing/RFLP; Targets: Broad fungal 28S identification; Comparator: Direct microscopy and/or fungal culture.	Narrative only Narrative/supportive only
OMD-2013-Rothmund Rothmund G et al. (2013) [89]	Nail subgroup information: n=60. Prospective six-test comparison Germany; Augsburg	Nail material: 60. Unique patient count is not inferred from specimen totals. Not clearly reported or mixed	Dermatophyte PCR; Targets: Dermatophytes; Comparator: Direct microscopy and/or fungal culture.	Narrative only Narrative/supportive only
OMD-2013-Miyajima Miyajima Y et al. (2013) [90]	Nail subgroup information: 32 clinical nails plus 39 healthy nail controls. Microscopy-positive clinical nail validation Japan	Nail material: 32. Unique patient count is not inferred from specimen totals. Not clearly reported or mixed	Real-time PCR with fluorescent probes; Targets: Pan-fungal plus *T. rubrum*/*T. mentagrophytes*; Comparator: Culture; Direct microscopy	Paired 2 × 2 D131, D132 Comparator- and stratum-specific discordance analysis
OMD-2012-Luk Luk NM et al. (2012) [91]	Nail subgroup information: n=120. Clinical nail comparison Hong Kong	Nail material: 120. Unique patient count is not inferred from specimen totals. Not clearly reported or mixed	Pan-dermatophyte PCR; Targets: Dermatophytes; Comparator: Culture; KOH microscopy	Paired 2 × 2 D133, D134 Comparator- and stratum-specific discordance analysis
OMD-2009-Ebihara Ebihara M et al. (2009) [92]	Nail subgroup information: n=50. Microscopy-positive spectrum molecular comparison Japan	Nail material: 50. Unique patient count is not inferred from specimen totals. Not clearly reported or mixed	28S nested PCR; Targets: Dermatophytes and selected non-dermatophytes; Comparator: Direct microscopy	Paired 2 × 2 D135, D136 Comparator- and stratum-specific discordance analysis
OMD-2009-Bontems Bontems O et al. (2009) [93]	Nail subgroup information: n=410. Microscopy-positive routine laboratory comparison Switzerland; Lausanne	Nail material: 410. Unique patient count is not inferred from specimen totals. Not clearly reported or mixed	28S PCR-RFLP; Targets: Broad fungal identification; Comparator: Direct microscopy and/or fungal culture.	Narrative only Narrative/supportive only
OMD-2010-Litz Litz CE et al. (2010) [94]	Nail subgroup information: n=550. Large clinical nail comparison United States; Dallas	Nail material: 550. Unique patient count is not inferred from specimen totals. Not clearly reported or mixed	CHS1 PCR; Targets: Dermatophytes; Comparator: Culture; PAS histology; KOH microscopy	Paired 2 × 2 D137, D138, D139 Comparator- and stratum-specific discordance analysis
OMD-2018-Adel-Taha Adel LA et al. (2018) [95]	Nail subgroup information: n=50. Clinical nail comparison Egypt	Nail material: 50. Unique patient count is not inferred from specimen totals. Recent antifungal treatment excluded	Multiplex dermatophyte PCR; Targets: Pan-dermatophyte plus *T. rubrum*/*T. mentagrophytes*; Comparator: Culture; KOH microscopy	Paired 2 × 2 D140, D141 Comparator- and stratum-specific discordance analysis
OMD-2011-Brasch Brasch J et al. (2011) [96]	Nail subgroup information: n=230 nails. Routine mixed-site study with exact nail subgroup Germany	Nail material: 230. Unique patient count is not inferred from specimen totals. Not clearly reported or mixed	T. rubrum-specific PCR; Targets: T. rubrum; Comparator: Culture	Paired 2 × 2 D142 Comparator- and stratum-specific discordance analysis
OMD-1998-Baek Baek SC et al. (1998) [97]	Culture-proven fungus-infected nails and normal nails; clinical denominator not reported. Spectrum-selected clinical/analytical feasibility study South Korea	Nail material: not reported. Unique patient count is not inferred from specimen totals. Not reported	18S rRNA PCR followed by HaeIII restriction analysis; Targets: Broad fungal detection with class/species-pattern differentiation; Comparator: KOH microscopy and fungal culture	Narrative only Narrative/supportive only
OMD-2018-Alvarez-Mosquera Álvarez-Mosquera I et al. (2018) [98]	626 unselected clinical specimens, including 440 toenails and 83 fingernails. Routine mixed-site clinical comparison Spain; Bilbao	Nail material: 523. Unique patient count is not inferred from specimen totals. Not reported	18S rRNA broad fungal PCR with sequencing; Targets: Broad fungal detection and sequencing; Comparator: KOH microscopy and fungal culture	Narrative only Narrative/supportive only
OMD-2025-Wang-RPA Wang Q et al. (2024) [99]	190 continuously collected nail samples from patients with suspected onychomycosis. Prospective clinical nail diagnostic study China; Beijing	Nail material: 190. Unique patient count is not inferred from specimen totals. Prior treatment not clearly reported	Pan-dermatophyte RPA-LFD followed by T. rubrum-specific RPA-LFD; Targets: Dermatophytes; reflex T. rubrum identification; Comparator: Culture; KOH microscopy	Paired 2 × 2 D143, D144 Comparator- and stratum-specific discordance analysis
OMD-2012-Dhib Dhib I et al. (2012) [100]	201 nails from suspected onychomycosis patients plus 80 healthy nail controls. Clinical nail comparison with healthy controls Tunisia; Sousse	Nail material: 281. Unique patient count is not inferred from specimen totals. Not reported	Conventional CHS1 pan-dermatophyte PCR; Targets: Dermatophytes; Comparator: Conventional composite; KOH microscopy; Culture	Paired 2 × 2 D145, D146, D147 Comparator- and stratum-specific discordance analysis
OMD-2019-Koo Koo SH et al. (2019) [101]	201 patients with suspected onychomycosis contributing 204 nail specimens. Prospective single-center clinical nail study Singapore	Nail material: 204. Unique patient count is not inferred from specimen totals. Previous oral antifungal treatment excluded	Two-tube multiplex hydrolysis-probe qPCR; Targets: Pan-dermatophytes, *Fusarium* spp., *T. rubrum* and *Microsporum* spp.; Comparator: Culture; Expanded standard	Paired 2 × 2 D148, D149, D150, D151 Comparator- and target-specific discordance analysis
OMD-2020-Ross Ross IL et al. (2020) [102]	156 prospectively tested nail specimens in the dermatophyte comparison. Prospective mixed-site study with exact nail subgroup Australia; South Australia	Nail material: 156. Unique patient count is not inferred from specimen totals. Not reported	AusDiagnostics Dermatophytes and Other Fungi multiplex tandem PCR; Targets: Dermatophytes plus selected yeasts and molds; discordance rows use dermatophyte endpoint; Comparator: Culture; Microscopy; Conventional composite	Paired 2 × 2 D152, D153, D154 Comparator- and stratum-specific discordance analysis
OMD-2021-Lin Lin BB et al. (2021) [103]	Part 3: 128 prospective nail samples; Part 2: 47 culture-positive nail samples. Three-part assay validation with prospective nail subgroup Australia; Victoria	Nail material: 128. Unique patient count is not inferred from specimen totals. Not reported	AusDiagnostics Dermatophyte & Fungi multiplex RT-PCR version 2; Targets: Dermatophytes; Comparator: Culture; Microscopy; Conventional composite	Paired 2 × 2 D155, D156, D157, D158 Comparator- and stratum-specific discordance analysis
OMD-2021-Cuchi-Burgos Cuchí-Burgos E et al. (2021) [104]	152 clinical nail samples selected at a 3:1 KOH-positive:negative ratio. Prospective KOH-enriched clinical workflow study Spain	Nail material: 152. Unique patient count is not inferred from specimen totals. Not reported	DermaGenius 2.0 multiplex real-time PCR; Targets: Dermatophytes and *Candida albicans*; Comparator: KOH microscopy; Culture	Paired 2 × 2 D159, D160 Comparator-specific discordance analysis; invalids excluded
OMD-2026-Sharma Sharma A et al. (2026) [28]	65 clinically suspected onychomycosis patients. Prospective cross-sectional clinical nail study India; New Delhi	Nail material: 65. Unique patient count is not inferred from specimen totals. Prior antifungal use discussed but not used as an exclusion criterion	FTD Dermatophytes commercial two-tube multiplex real-time PCR; Targets: Common dermatophytes with species identification; Comparator: Culture; KOH microscopy	Paired 2 × 2 D161, D162 Comparator-specific discordance analysis
OMD-2004-Menotti Menotti J et al. (2004) [9]	Series 1: 48 patients with optimally collected nail samples; series 2: 88 patients contributing a non-optimal clipping for PCR and an optimally collected specimen from the same nail for conventional mycology. Prospective two-series clinical nail comparison France; Paris	Nail material: 224. Unique patient count is not inferred from specimen totals. Not reported; authors proposed possible use after treatment	Broad fungal PCR followed by restriction-fragment-length polymorphism; Targets: Dermatophytes versus Scytalidium; does not detect Candida or other molds and does not identify individual dermatophyte species; Comparator: Conventional composite	Paired 2 × 2 D163, D164 Comparator- and sampling-stratum-specific discordance analysis
OMD-2007-Gupta-DoublePCR Gupta AK et al. (2007) [10]	62 patients with toenail onychomycosis and a prior T. rubrum-positive culture. Culture-history-enriched clinical nail comparison Canada; Toronto/London, Ontario	Nail material: 62. Unique patient count is not inferred from specimen totals. Mixed; at least 15 culture-negative/actin-PCR-positive samples were obtained during antifungal treatment	Double-round/seminested PCR targeting the actin gene and ITS1; combined endpoint positive if either PCR was positive; Targets: T. rubrum DNA; both assays cross-reacted with *T. violaceum* in analytical specificity testing; Comparator: Culture; KOH microscopy	Paired 2 × 2 D165, D166, D167, D168, D169, D170 Comparator- and assay-variant-specific discordance analysis
OMD-2024-Felek Felek R et al. (2024) [8]	46 clinically suspected cases evaluated; 37 positive by culture and/or EUROArray included; adults aged 19–78 years. Single-center clinical laboratory comparison; June 2020–July 2021; recruitment sequence not specified Akdeniz University Hospital, Antalya, Turkey	Nail material: 37. Unique patient count is not inferred from specimen totals. 21/37 (56.8%) prior systemic/topical antifungals; timing unavailable; no treatment-stratified pairs	EUROArray Dermatomycosis PCR-based microarray; DNA extracted directly from nails using QIAamp DNA Mini Kit; Targets: Panel for 23 dermatophytes, three yeasts and three molds; lacks Aspergillus and Alternaria targets; Comparator: Culture	Paired 2 × 2 D171, D172, D173, D174 Restricted paired evidence; excluded from historical and broader culture summaries because of positive-test selection

Appendix 2

Complete Paired Comparison Map

Tables 10-11 are joined by the stable comparison identifier. They reproduce 170 historical comparison rows, four newly extracted Felek R comparisons (D171-D174), and one explicitly derived valid-only alternative, D089V. D089V replaces D089 only in the broader revision analyses. Rows sharing a report, specimens, targets, treatment times, or thresholds must not be summed as independent studies. A source-reported row may still have invalid-result or denominator limitations even when its four cells sum to N.

**Table 10:** Population, assay, comparator, and source qualifications for every paired row. OM: Onychomycosis; rDNA: Ribosomal Deoxyribonucleic Acid; 18S rDNA: 18S Ribosomal DNA; 28S: 28S Ribosomal RNA gene/region; ITS: Internal Transcribed Spacer; ITS1: Internal Transcribed Spacer 1; ITS2: Internal Transcribed Spacer 2; KOH: Potassium Hydroxide; PAS: Periodic Acid-Schiff; GMS: Grocott Methenamine Silver; NDM: Non-Dermatophyte Mold; RFLP: Restriction Fragment Length Polymorphism; PCR-RFLP: Polymerase Chain Reaction-Restriction Fragment Length Polymorphism; TRFLP/T-RFLP: Terminal Restriction Fragment Length Polymorphism; PCR-TRFLP: Polymerase Chain Reaction-Terminal Restriction Fragment Length Polymorphism; OD: Optical Density; RPA: Recombinase Polymerase Amplification; RPA-LFD: Recombinase Polymerase Amplification-Lateral Flow Dipstick; LAMP: Loop-Mediated Isothermal Amplification; LFD: Lateral Flow Dipstick; CHS1: Chitin Synthase 1; TEF1-alpha/TEF1-α: Translation Elongation Factor 1-alpha; OIAD: Onychodystrophy Infectious Agent Detection; DE: Direct Examination; n/N: Number of specimens, patients, or observations; M+/M-: Molecular-test positive/molecular-test negative; C+/C-: Culture positive/culture negative.

Row / report	Population and unit	Molecular endpoint / comparator	Extraction and source qualification
D001 OMD-2006-Walberg [11]	All suspected OM; Unit: Nail specimen	18S rDNA PCR + sequencing; Target: Dermatophyte detection; Comparator: Culture; Dermatophyte culture positivity	Reconstruction status: No-binary cells from Table I; 57 includes 8 species/genus-discordant binary positives; Source: Table I; Extraction note: 49 exact *T. rubrum* concordant; do not use 49 as binary +/+.
D002 OMD-2024-Gupta-US [49]	Paired PCR–histology subset; Unit: Toenail specimen	OIAD multiplex PCR; Target: Any fungal target on panel; Comparator: Histopathology; PAS/GMS fungal elements	Reconstruction status: No-read directly from Figure 4; Source: Figure 4; Extraction note: Use n=41,223; text elsewhere reports inconsistent n=41,284.
D003 OMD-2023-Marin-Maldonado [50]	High/moderate-quality Sanger subset; Unit: Toenail specimen	Onycho+ qPCR; Target: Dermatophyte; Comparator: PAS histology; Fungal elements	Reconstruction status: No-Table 3 cross-tab; Source: Table 3; Extraction note: Not representative of all 237 valid qPCR samples.
D004 OMD-2023-Marin-Maldonado [50]	High/moderate-quality Sanger subset; Unit: Toenail specimen	Onycho+ qPCR; Target: Dermatophyte; Comparator: ITS2 Sanger sequencing; Dermatophyte sequence	Reconstruction status: No-Table 3 cross-tab; Source: Tables 3 and 5; Extraction note: Zero cells retained without continuity correction; current ratio and interval handling is described in Methods.
D005 OMD-2026-Dey [32]	All suspected OM; Unit: Nail specimen	Universal ITS PCR; Target: Any fungal pathogen as defined by authors; Comparator: Culture; Dermatophyte or *C. albicans* culture	Reconstruction status: No-Table 5; Source: Table 5; Extraction note: NDM excluded from comparator-positive definition.
D006 OMD-2026-Villanueva-Reyes [46]	51 OM cases + 52 evaluable healthy controls; Unit: Nail specimen	Nanopore ITS sequencing; Target: Any fungal sequence classified positive; Comparator: Composite; KOH and/or culture	Reconstruction status: No-performance table; Source: Diagnostic-performance table; Extraction note: Eight healthy nails sequencing-positive.
D007 OMD-2026-Villanueva-Reyes [46]	Composite-confirmed OM cases; Unit: Nail specimen	Nanopore ITS sequencing; Target: Any fungal sequence classified positive; Comparator: Culture; Any fungal culture	Reconstruction status: No-Results counts; Source: Results; Extraction note: Ten sequencing-positive OM cases interpreted as transient microbiota after conventional review.
D008 OMD-2018-Bao [52]	All suspected OM specimens; Unit: Nail specimen	ITS PCR + sequencing; Target: Any fungal detection; Comparator: Culture; Any fungal growth judged positive	Reconstruction status: No-reported cross-tab; Source: Diagnostic comparison table; Extraction note: PCR-positive total 102; culture-positive total 87.
D009 OMD-2019-Gustafson [18]	Initial validation cohort; Unit: Nail specimen	Real-time PCR panel; Target: Any fungal class; Comparator: Culture; Any fungal culture	Reconstruction status: Yes-summed class table; exact arithmetic; Source: Table 2; Extraction note: The reported 206 discordances are class/species discordances, not binary positivity discordances.
D010 OMD-2019-Gustafson [18]	Sequential three-test cohort; Unit: Nail specimen	Real-time PCR panel; Target: Any fungal class; Comparator: Histopathology; PAS/GMS fungal elements	Reconstruction status: No-Table 6; Source: Table 6; Extraction note: PCR positivity 1,238; histology positivity 1,449.
D011 OMD-2019-Gustafson [18]	Sequential three-test cohort; Unit: Nail specimen	Real-time PCR panel; Target: Any fungal class; Comparator: Culture; Any fungal culture	Reconstruction status: Yes-reconstructed from Table 5A eight patterns; Source: Table 5A; Extraction note: Exact reconstruction; avoid double-counting with n=425 cohort if cohorts overlap.
D012 OMD-2007-Garg [53]	All suspected OM; Unit: Nail specimen	Pan-dermatophyte nested PCR; Target: Dermatophyte; Comparator: Culture; Dermatophyte culture	Reconstruction status: No-Table 1; Source: Table 1; Extraction note: All 83 treated nonresponders were PCR+/culture−.
D013 OMD-2007-Garg [53]	All suspected OM; Unit: Nail specimen	Pan-dermatophyte nested PCR; Target: Dermatophyte; Comparator: KOH microscopy; Fungal elements	Reconstruction status: No-Table 1; Source: Table 1; Extraction note: Target mismatch: PCR is dermatophyte-specific; KOH detects fungal elements nonspecifically.
D014 OMD-2007-Garg [53]	Previously treated nonresponders; Unit: Nail specimen	Pan-dermatophyte nested PCR; Target: Dermatophyte; Comparator: Culture; Dermatophyte culture	Reconstruction status: No-Table 1; Source: Table 1; Extraction note: Do not pool with untreated cases.
D015 OMD-2007-Garg [53]	Untreated; Unit: Nail specimen	Pan-dermatophyte nested PCR; Target: Dermatophyte; Comparator: Culture; Dermatophyte culture	Reconstruction status: No-Table 1; Source: Table 1; Extraction note: Matched OR undefined because both discordant cells are zero.
D016 OMD-2007-Brillowska-Dabrowska [23]	Routine clinically diseased nails; Unit: Nail specimen	Five-hour multiplex PCR; Target: Dermatophyte; Comparator: Culture; Dermatophyte culture	Reconstruction status: Yes-reconstructed from detailed results; Source: Results; Extraction note: Four culture-positive/PCR-negative cases and 27 PCR-positive/culture-negative cases.
D017 OMD-2006-Kardjeva [21]	Direct nail cohort only; Unit: Nail specimen	T1/ITS/SCOP molecular workflow; Target: Any fungal agent; Comparator: Culture; Any fungal culture	Reconstruction status: No-Table 2; Source: Table 2; Extraction note: Exclude separate n=66 strain sample from synthesis.
D018 OMD-2006-Kardjeva [21]	Direct nail cohort only; Unit: Nail specimen	T1/ITS/SCOP molecular workflow; Target: Any fungal agent; Comparator: Microscopy; Fungal elements	Reconstruction status: No-Table 2; Source: Table 2; Extraction note: Seven PCR-negative samples had inhibitors.
D019 OMD-2026-Guegan [16]	Nail subgroup; Unit: Nail specimen	DendrisKIT DP + DendrisCHIP; Target: Dermatophyte; Comparator: Culture; Dermatophyte culture	Reconstruction status: No-Table 3; Source: Table 3; Extraction note: PCR+/culture− is not treated as automatically false positive.
D020 OMD-2026-Guegan [16]	Nail subgroup; Unit: Nail specimen	DendrisKIT DP + DendrisCHIP; Target: Dermatophyte; Comparator: Composite; Direct examination and/or dermatophyte culture	Reconstruction status: No-Table 3; Source: Table 3; Extraction note: Composite broadens comparator beyond dermatophytes because DE is nonspecific.
D021 OMD-2022-Pospischil [44]	Conditional PCR/sequencing group; Unit: Nail specimen	Direct-nail 28S PCR + sequencing; Target: Any fungal agent; Comparator: Culture; Positive culture versus negative/contaminated	Reconstruction status: No-summed Table 2 categories; Source: Table 2; Extraction note: Do not mix with complete-paired cohorts; nearly all were microscopy-positive.
D022 OMD-2012-Verrier [54]	Microscopy-positive abnormal nails; Unit: Nail specimen	28S PCR-TRFLP; Target: Any fungal signal; Comparator: Culture; Any non-sterile fungal culture	Reconstruction status: Yes-binary detection derived from Table 2 totals; Source: Table 2; Extraction note: 171 includes 162 identified agents plus 9 off-ladder fungal peaks among sterile cultures.
D023 OMD-2022-Bieber [29]	Suspected OM cases; Unit: Nail specimen	EUROArray Dermatomycosis; Target: Any target fungus; Comparator: Culture; Any positive fungal culture	Reconstruction status: Yes-algebra from single and combined yields; Source: Figure 1 and Table 1; Extraction note: Cells are exact under reported set unions but not directly tabulated.
D024 OMD-2022-Bieber [29]	Suspected OM cases; Unit: Nail specimen	EUROArray Dermatomycosis; Target: Any target fungus; Comparator: Microscopy; Direct fluorescence fungal elements	Reconstruction status: Yes-algebra from single and combined yields; Source: Figure 1 and Table 1; Extraction note: Cells reconstructed from DNA 30, microscopy 26 and union 35.
D025 OMD-2022-Bieber [29]	Suspected OM cases; Unit: Nail specimen	EUROArray Dermatomycosis; Target: Any target fungus; Comparator: Composite; Microscopy and/or culture	Reconstruction status: Yes-algebra from union yields; Source: Figure 1 and Table 1; Extraction note: Healthy controls are not included in this 2 × 2.
D026 OMD-2024-Katsiaunis [34]	All paired patients; Unit: Patient/nail	Targeted PCR panel; Target: Dermatophyte; Comparator: Culture; Dermatophyte culture	Reconstruction status: No-Table I; Source: Table I; Extraction note: 84/87 PCR+/culture− cases were histology-positive.
D027 OMD-2024-Katsiaunis [34]	All paired patients; Unit: Patient/nail	Targeted PCR panel; Target: NDM; Comparator: Culture; NDM culture	Reconstruction status: No-Table I; Source: Table I; Extraction note: 17/18 PCR+/culture− cases were histology-positive.
D028 OMD-2024-Harel [30]	Nail subgroup; Unit: Nail specimen	Novaplex dermatophyte multiplex qPCR; Target: Dermatophyte; Comparator: Culture; Dermatophyte culture	Reconstruction status: No-Table 5; Source: Table 5; Extraction note: PCR added 17 net positives over culture in nails.
D029 OMD-2024-Harel [30]	Nail subgroup; Unit: Nail specimen	Novaplex dermatophyte multiplex qPCR; Target: Dermatophyte; Comparator: Final diagnosis composite; Author final diagnostic classification	Reconstruction status: No-Table 5; Source: Table 5; Extraction note: Do not treat the composite as independent of the index test.
D030 OMD-2007-Savin [56]	Regional proximal samples; positive-only threshold; Unit: Nail specimen	Onychodiag PCR-ELISA; Target: Dermatophyte; Comparator: Culture; Dermatophyte culture	Reconstruction status: No-reported cross-tab; Source: Tables 2–3; Extraction note: Primary positive threshold OD ≥0.5.
D031 OMD-2007-Savin [56]	Regional proximal samples; gray-zone-positive sensitivity; Unit: Nail specimen	Onychodiag PCR-ELISA; Target: Dermatophyte; Comparator: Culture; Dermatophyte culture	Reconstruction status: No-reported cross-tab; Source: Tables 2–3; Extraction note: Do not combine with positive-only threshold row.
D032 OMD-2007-Savin [56]	Regional proximal samples; positive-only threshold; Unit: Nail specimen	Onychodiag PCR-ELISA; Target: Dermatophyte; Comparator: Direct examination; Fungal elements	Reconstruction status: No-reported cross-tab; Source: Tables 2–3; Extraction note: Dermatophyte-specific PCR versus nonspecific direct examination.
D033 OMD-2007-Savin [56]	Distal samples linked to proximal comparator; positive-only threshold; Unit: Linked nail specimen	Onychodiag PCR-ELISA; Target: Dermatophyte; Comparator: Proximal culture; Dermatophyte culture from linked proximal regional sample	Reconstruction status: No-reported cross-tab; Source: Table 4; Extraction note: Keep outside identical-specimen main pool.
D034 OMD-2007-Savin [56]	Distal samples linked to proximal comparator; gray-zone-positive sensitivity; Unit: Linked nail specimen	Onychodiag PCR-ELISA; Target: Dermatophyte; Comparator: Proximal culture; Dermatophyte culture from linked proximal regional sample	Reconstruction status: No-reported cross-tab; Source: Table 4; Extraction note: Zero M−/culture+ cell after gray-zone reclassification.
D035 OMD-2007-Savin [56]	Distal samples linked to proximal comparator; positive-only threshold; Unit: Linked nail specimen	Onychodiag PCR-ELISA; Target: Dermatophyte; Comparator: Proximal direct examination; Fungal elements from linked proximal regional sample	Reconstruction status: No-reported cross-tab; Source: Table 4; Extraction note: Zero M+/DE− cell at the positive-only threshold.
D036 OMD-2007-Savin [56]	Routine regional samples; positive-only threshold; Unit: Nail specimen	Onychodiag PCR-ELISA; Target: Dermatophyte; Comparator: Culture; Dermatophyte culture	Reconstruction status: No-reported cross-tab; Source: Table 5; Extraction note: All culture-positive routine regional samples were PCR-positive.
D037 OMD-2007-Savin [56]	Routine regional samples; gray-zone-positive sensitivity; Unit: Nail specimen	Onychodiag PCR-ELISA; Target: Dermatophyte; Comparator: Culture; Dermatophyte culture	Reconstruction status: No-reported cross-tab; Source: Table 5; Extraction note: Do not combine with positive-only threshold row.
D038 OMD-2007-Savin [56]	Routine regional samples; positive-only threshold; Unit: Nail specimen	Onychodiag PCR-ELISA; Target: Dermatophyte; Comparator: Direct examination; Fungal elements	Reconstruction status: No-reported cross-tab; Source: Table 5; Extraction note: The reported combined conventional table is incomplete; only the direct-examination row is used.
D039 OMD-2024-Navarro-Perez [31]	All suspected toenail OM; Unit: Toenail specimen	Direct-nail PCR; Target: Laboratory fungal diagnosis; Comparator: Culture; Positive fungal culture	Reconstruction status: Yes-exact inclusion-exclusion from PCR 60, culture 54, union 87; Source: Results; Extraction note: Intersection = 60 + 54 − 87 = 27.
D040 OMD-2024-Evrard [45]	Systematic testing cohort; Unit: Nail specimen	DermaGenius multiplex PCR; Target: Panel fungal pathogen; Comparator: Culture; Pathogenic fungal culture	Reconstruction status: Yes-exact from reported margins and union; Source: Tables 2–3; Extraction note: PCR 43, culture 41, systematic union 55.
D041 OMD-2024-Evrard [45]	Systematic testing cohort; Unit: Nail specimen	DermaGenius multiplex PCR; Target: Panel fungal pathogen; Comparator: Systematic composite; PCR and/or pathogen culture	Reconstruction status: Yes-set definition; Source: Tables 2–3; Extraction note: Index-positive/composite-negative is structurally zero.
D042 OMD-2024-Evrard [45]	First-line strategy; Unit: Nail specimen	DermaGenius PCR + calcofluor microscopy + selective culture; Target: Algorithm-positive fungal diagnosis; Comparator: Systematic composite; Systematic PCR and/or pathogen culture	Reconstruction status: No-reported performance; Source: Table 4; Extraction note: Selective culture required in only 13% of samples.
D043 OMD-2017-Iwanaga [43]	Baseline before terbinafine; Unit: Nail specimen	Dermatophyte actin qPCR; Target: Dermatophyte DNA; Comparator: Culture; Dermatophyte culture	Reconstruction status: No-reported counts; Source: Table 1; Extraction note: One baseline patient lacked a common evaluable culture/qPCR pair.
D044 OMD-2017-Iwanaga [43]	Week 8 during terbinafine; Unit: Nail specimen	Dermatophyte actin qPCR; Target: Dermatophyte DNA; Comparator: Culture; Dermatophyte culture	Reconstruction status: No-reported counts; Source: Table 1; Extraction note: Do not pool with baseline or week 16.
D045 OMD-2017-Iwanaga [43]	Week 16 during terbinafine; Unit: Nail specimen	Dermatophyte actin qPCR; Target: Dermatophyte DNA; Comparator: Culture; Dermatophyte culture	Reconstruction status: No-reported counts; Source: Table 1; Extraction note: Do not pool with baseline or week 8.
D046 OMD-2017-Iwanaga [43]	Baseline before terbinafine; Unit: Nail specimen	Dermatophyte actin qPCR; Target: Dermatophyte DNA; Comparator: KOH microscopy; Fungal elements	Reconstruction status: No-reported counts; Source: Table 2; Extraction note: Matched discordance OR undefined.
D047 OMD-2017-Iwanaga [43]	Week 8 during terbinafine; Unit: Nail specimen	Dermatophyte actin qPCR; Target: Dermatophyte DNA; Comparator: KOH microscopy; Fungal elements	Reconstruction status: No-reported counts; Source: Table 2; Extraction note: PCR target and KOH target differ.
D048 OMD-2017-Iwanaga [43]	Week 16 during terbinafine; Unit: Nail specimen	Dermatophyte actin qPCR; Target: Dermatophyte DNA; Comparator: KOH microscopy; Fungal elements	Reconstruction status: No-reported counts; Source: Table 2; Extraction note: PCR target and KOH target differ.
D049 OMD-2018-Lubis [57]	All suspected OM; Unit: Nail specimen	PCR-RFLP; Target: Fungal/dermatophyte detection; Comparator: Culture; Positive fungal culture	Reconstruction status: No-reported cross-tab; Source: Table 2; Extraction note: Do not equate culture-negative molecular detection with a false positive.
D050 OMD-2014-Han [58]	All patient nail specimens; Unit: Nail specimen	PCR oligonucleotide array; Target: Target dermatophyte or *C. albicans*; Comparator: Culture; Target organism recovered by culture	Reconstruction status: No-reported counts; Source: Results; Extraction note: Ten healthy nails were array-negative and remain outside this row.
D051 OMD-2014-Han [58]	All patient nail specimens; Unit: Nail specimen	PCR oligonucleotide array; Target: Target dermatophyte or *C. albicans*; Comparator: Adjudicated composite; Culture plus selected molecular/KOH/clinical resolver	Reconstruction status: No-reported counts; Source: Results and Table 3; Extraction note: Composite is not independent of the index test.
D052 OMD-2026-Wu [47]	All clinical nail specimens; Unit: Nail specimen	Multi-tube RPA; Target: Any target fungal agent; Comparator: Culture; Any positive fungal culture	Reconstruction status: Yes-summed exact eight three-test patterns; Source: Table 3; Extraction note: Bayesian latent-class estimates remain outside this direct comparator row.
D053 OMD-2026-Wu [47]	All clinical nail specimens; Unit: Nail specimen	Multi-tube RPA; Target: Any target fungal agent; Comparator: Microscopy; Fungal elements	Reconstruction status: Yes-summed exact eight three-test patterns; Source: Table 3; Extraction note: RPA positivity 58; microscopy positivity 61.
D054 OMD-2021-Kabtani [59]	Nail subgroup; Unit: Nail specimen	In-house real-time PCR; Target: Dermatophyte; Comparator: Conventional diagnosis; Dermatophyte-positive conventional classification	Reconstruction status: No-Table 6; Source: Table 6; Extraction note: Microscopy any-fungal positivity is not used as the dermatophyte comparator.
D055 OMD-2021-Kabtani [59]	Nail subgroup; Unit: Nail specimen	Eurobio commercial real-time PCR kit; Target: Dermatophyte; Comparator: Conventional diagnosis; Dermatophyte-positive conventional classification	Reconstruction status: No-Table 6/text; Source: Table 6 and Results; Extraction note: Table 7 appears to omit one commercial-kit positive; discrepancy flagged, not silently reconciled.
D056 OMD-2010-Sato [22]	Microscopy-confirmed nail specimens; Unit: Nail specimen	PCR-DNA microarray; Target: Universal fungal signal; Comparator: Culture; Successful fungal culture identification	Reconstruction status: No-reported counts; Source: Table 3; Extraction note: Three specimens had universal signal without species identification.
D057 OMD-2010-Sato [22]	Microscopy-confirmed nail specimens; Unit: Nail specimen	PCR-DNA microarray; Target: Successful species identification; Comparator: Culture; Successful fungal culture identification	Reconstruction status: No-reported counts; Source: Table 3; Extraction note: Do not mix universal detection and species-identification endpoints.
D058 OMD-2023-Gupta-NDM [20]	All toenails; binary fungal endpoint; Unit: Toenail specimen	OIAD multiplex PCR; Target: Any fungal target excluding isolated Pseudomonas; Comparator: Histopathology; PAS/GMS fungal elements	Reconstruction status: Yes-summed reported PCR categories against histology; Source: Table 2; Extraction note: Pseudomonas-only PCR category is molecular-fungal-negative for this endpoint.
D059 OMD-2023-Gupta-NDM [20]	All toenails; NDM endpoint; Unit: Toenail specimen	OIAD multiplex PCR; Target: Targeted NDM genus; Comparator: Culture; NDM recovered by culture	Reconstruction status: No-Table 3; Source: Table 3; Extraction note: Off-panel molds can contribute to PCR−/culture+ cells.
D060 OMD-2020-Pakshir [61]	Suspected OM nail specimens; Unit: Nail specimen	TEF1-alpha PCR; Target: Candida; Comparator: Culture; Candida culture	Reconstruction status: No-Table 2; Source: Table 2; Extraction note: Candida DNA may reflect colonization; causality is not established by detection alone.
D061 OMD-2022-Trovato [41]	All patients; Unit: Toenail specimen	Real-time multiplex PCR; Target: Dermatophyte; Comparator: Microscopy; Fungal elements	Reconstruction status: No-Table 2; Source: Table 2; Extraction note: Analyze treatment strata where possible.
D062 OMD-2022-Trovato [41]	All patients; Unit: Toenail specimen	Real-time multiplex PCR; Target: Dermatophyte; Comparator: Culture; Dermatophyte culture	Reconstruction status: No-Table 2; Source: Table 2; Extraction note: Prior empirical treatment likely suppresses culture.
D063 OMD-2022-Trovato [41]	All patients; Unit: Toenail specimen	Real-time multiplex PCR; Target: Dermatophyte; Comparator: Conventional composite; Microscopy and/or dermatophyte culture	Reconstruction status: No-Table 2; Source: Table 2; Extraction note: Do not pool across treatment status.
D064 OMD-2022-Trovato [41]	Untreated; Unit: Toenail specimen	Real-time multiplex PCR; Target: Dermatophyte; Comparator: Conventional composite; Microscopy and/or dermatophyte culture	Reconstruction status: Yes-reported sensitivity/specificity and stratum totals; Source: Results; Extraction note: Sensitivity 68.4%, specificity 100%.
D065 OMD-2022-Trovato [41]	Previously empirically treated; Unit: Toenail specimen	Real-time multiplex PCR; Target: Dermatophyte; Comparator: Conventional composite; Microscopy and/or dermatophyte culture	Reconstruction status: Yes-reported sensitivity/specificity and stratum totals; Source: Results; Extraction note: Sensitivity 92.6%, specificity 100%; do not pool with untreated stratum.
D066 OMD-2017-Vrioni [62]	All suspected OM; Unit: Nail specimen	Dermatophyte multiplex PCR; Target: Dermatophyte; Comparator: Culture; Dermatophyte culture	Reconstruction status: Yes-Figure 2 plus reported margins; Source: Figure 2 and Results; Extraction note: Culture dermatophyte-positive n=56; PCR-positive n=86.
D067 OMD-2017-Vrioni [62]	All suspected OM; Unit: Nail specimen	Dermatophyte multiplex PCR; Target: Dermatophyte; Comparator: Conventional composite; Microscopy and/or dermatophyte culture	Reconstruction status: Yes-reported margins with sensitivity/specificity; Source: Results and reported performance; Extraction note: Do not equate PCR+/composite− with false positivity.
D068 OMD-2026-Gellrich [35]	Nail subgroup; Unit: Nail specimen	EUROArray Dermatomycosis; Target: Dermatophyte; Comparator: Culture; Dermatophyte culture	Reconstruction status: Yes-site totals plus overall overlap; Source: Table 2 and overall Results; Extraction note: Abstract/text/Table 2 use n=52; Table 1 reports n=51.
D069 OMD-2023-Guschin [65]	Nail subgroup; Unit: Nail specimen	AmpliPrime Dermatophytes RT-PCR; Target: Dermatophyte; Comparator: Culture; Dermatophyte culture	Reconstruction status: Yes-Table 2 margins plus explicit overlap; Source: Table 2 and Results; Extraction note: All nail culture-positive dermatophytes were PCR-positive.
D070 OMD-2023-Guschin [65]	Nail subgroup; Unit: Nail specimen	AmpliPrime Dermatophytes RT-PCR; Target: Dermatophyte; Comparator: KOH microscopy; Fungal elements	Reconstruction status: Yes-Table 2 margins plus explicit discordances; Source: Table 2 and Results; Extraction note: Two nail microscopy-only cases were PCR/culture-negative.
D071 OMD-2022-Makled [66]	All clinically diagnosed OM; Unit: Nail specimen	Universal ITS PCR; Target: Any fungal signal; Comparator: Culture; Any positive fungal culture	Reconstruction status: No-Table 3; Source: Table 3; Extraction note: PCR was run on all 100 nails; only 30 PCR products were sequenced.
D072 OMD-2019-Camacaro [67]	All clinical tinea unguium patients; Unit: Patient/nail	Multiplex PCR; Target: Dermatophyte; Comparator: Culture; Dermatophyte culture	Reconstruction status: Yes-margins plus reported specificity; Source: Results and diagnostic performance; Extraction note: Reported 97% sensitivity conflicts with the discrete reconstructed table; cells imply 100%.
D073 OMD-2013-Chandran [27]	Microscopy-positive suspected OM; Unit: Nail specimen	Dermatophyte PCR Kit; Target: Dermatophyte; Comparator: Culture; Dermatophyte culture	Reconstruction status: No-Figure 2; Source: Figure 2 and Results; Extraction note: Dermatophyte culture n=38; PCR n=77; four PCR-positive samples had *Fusarium* culture.
D074 OMD-2013-Chandran [27]	Microscopy-positive suspected OM; Unit: Nail specimen	Dermatophyte PCR Kit; Target: Dermatophyte; Comparator: Culture; Any positive fungal culture	Reconstruction status: No-Figure 2; Source: Figure 2 and Results; Extraction note: Do not combine with target-matched culture row.
D075 OMD-2013-Chandran [27]	Microscopy-positive suspected OM; Unit: Nail specimen	Dermatophyte PCR Kit; Target: Dermatophyte; Comparator: Direct microscopy; Fungal elements	Reconstruction status: Yes-entry criterion plus PCR margin; Source: Methods and Results; Extraction note: All analyzed samples were microscopy-positive.
D076 OMD-2017-Hafirassou [37]	Clinical OM cases; Unit: Nail specimen	Pandermatophyte RT-PCR; Target: Dermatophyte; Comparator: Culture; Dermatophyte culture	Reconstruction status: Yes-reported sensitivity and margins; Source: Table 1 and Results; Extraction note: PCR total 42; dermatophyte culture 20; 18 overlap.
D077 OMD-2017-Hafirassou [37]	Clinical OM cases; Unit: Nail specimen	Pandermatophyte RT-PCR; Target: Dermatophyte; Comparator: Direct microscopy; Fungal elements	Reconstruction status: No-Table 1; Source: Table 1; Extraction note: Healthy controls excluded from this row.
D078 OMD-2017-Hafirassou [37]	Clinical OM cases; raw amplification endpoint; Unit: Nail specimen	Panfungal RT-PCR; Target: Any fungal amplification band; Comparator: Culture; Any positive fungal culture	Reconstruction status: Yes-reported margins/overlap; Source: Results; Extraction note: Do not equate raw amplification with sequence-confirmed identification.
D079 OMD-2017-Hafirassou [37]	Clinical OM cases; sequence-confirmed endpoint; Unit: Nail specimen	Panfungal RT-PCR plus sequencing; Target: Successful fungal identification; Comparator: Culture; Any positive fungal culture	Reconstruction status: Yes-Table 1 pattern counts; Source: Table 1; Extraction note: Six sequence-confirmed identifications occurred in culture-negative nails.
D080 OMD-2022-Gupta-Therapy [42]	Baseline before efinaconazole; Unit: Patient/toenail	Targeted fungal PCR; Target: Dermatophyte; Comparator: Culture; Dermatophyte culture	Reconstruction status: No-Table 1; Source: Table 1; Extraction note: All 28 were culture-positive dermatophyte cases at baseline.
D081 OMD-2022-Gupta-Therapy [42]	Month 24 during/after topical therapy; Unit: Patient/toenail	Targeted fungal PCR; Target: Any targeted fungal agent; Comparator: Culture; Any positive fungal culture	Reconstruction status: No-Table 1; Source: Table 1; Extraction note: The single culture-positive NDM case was also PCR-positive.
D082 OMD-2022-Gupta-Therapy [42]	Month 24 during/after topical therapy; Unit: Patient/toenail	Targeted fungal PCR; Target: Any targeted fungal agent; Comparator: KOH/culture composite; Positive KOH and/or culture	Reconstruction status: Yes-patient-level Table 1; Source: Table 1; Extraction note: Do not pool with baseline or treatment-naive cohorts.
D083 OMD-2014-Dhib [14]	All suspected toenail OM; Unit: Toenail specimen	Multiplex PCR; Target: Dermatophyte; Comparator: Culture; Dermatophyte culture	Reconstruction status: No-Table 3; Source: Table 3; Extraction note: All 132 culture-positive dermatophytes were PCR-positive.
D084 OMD-2014-Dhib [14]	All suspected toenail OM; Unit: Toenail specimen	Multiplex PCR; Target: Dermatophyte; Comparator: Direct microscopy; Fungal elements	Reconstruction status: Yes-Table 3 and text; Source: Table 3; Extraction note: All microscopy-positive samples were PCR-positive according to reported pattern table.
D085 OMD-2016-Gong [26]	All case/control-spectrum nails; Unit: Nail specimen	Novel real-time PCR; Target: Dermatophyte; Comparator: Culture; Dermatophyte culture	Reconstruction status: Yes-summed Table 3 patterns; Source: Table 3; Extraction note: Two non-dermatophyte cultures are target-negative for this row.
D086 OMD-2016-Gong [26]	All case/control-spectrum nails; Unit: Nail specimen	Novel real-time PCR; Target: Dermatophyte; Comparator: Direct microscopy; Fungal elements	Reconstruction status: Yes-summed Table 3 patterns; Source: Table 3; Extraction note: Do not substitute latent-class estimates for paired cells.
D087 OMD-2020-Caldwell [17]	Clinically suspected hallux OM; Unit: Patient/hallux nail	PathoGenius multiplex PCR; Target: Panel fungal pathogen; Comparator: PAS histopathology; Septate fungal elements	Reconstruction status: No-reported contingency table; Source: Table 1; Extraction note: PCR panel excludes unrepresented organisms; PAS positivity was higher.
D088 OMD-2019-Hayette [19]	Archived nail clippings; Unit: Nail specimen	DermaGenius Nail RT-PCR; Target: Dermatophyte or *C. albicans*; Comparator: PAS histology; Fungal elements	Reconstruction status: No-Table 2; Source: Table 2; Extraction note: Index target narrower than generic fungal histology.
D089 OMD-2019-Hayette [19]	Archived nail clippings; Unit: Nail specimen	DermaGenius Nail RT-PCR; Target: Dermatophyte or *C. albicans*; Comparator: Culture; Dermatophyte or *C. albicans* culture	Reconstruction status: No-Table 2; Source: Table 2; Extraction note: Eight inhibited/negative PCRs included in index-negative cells.
D090 OMD-2019-Hayette [19]	Archived nail clippings; Unit: Nail specimen	DermaGenius Nail RT-PCR; Target: Dermatophyte or *C. albicans*; Comparator: Conventional composite; Positive histology and/or target culture	Reconstruction status: No-Table 2; Source: Table 2; Extraction note: Do not combine with single-comparator rows.
D091 OMD-2011-Berk [68]	Clinically suspected OM cohort; Unit: Patient/nail	*T. rubrum* TaqMan RT-PCR; Target: *T. rubrum*; Comparator: Direct microscopy; Fungal elements	Reconstruction status: No-English abstract/results; Source: Abstract and Results; Extraction note: Twenty healthy control nails were all negative and are outside this row.
D092 OMD-2011-Berk [68]	Clinically suspected OM cohort; Unit: Patient/nail	*T. rubrum* TaqMan RT-PCR; Target: *T. rubrum*; Comparator: Culture; *T. rubrum* culture	Reconstruction status: Yes-reported margins and overlap; Source: Abstract and Results; Extraction note: All 20 culture-positive *T. rubrum* samples were PCR-positive.
D093 OMD-2023-Debuysschere [15]	All suspected OM nail samples; Unit: Nail specimen	Novaplex dermatophyte RT-PCR; Target: Dermatophyte; Comparator: Culture; Dermatophyte culture	Reconstruction status: No-reported counts; Source: Results and Figure 1; Extraction note: DermaGenius confirmed 28 of 29 PCR+/culture− results.
D094 OMD-2010-Brillowska-Routine [12]	Routine suspected tinea unguium; Unit: Nail specimen	Optimized 5-hour multiplex PCR; Target: Dermatophyte; Comparator: Culture; Dermatophyte culture	Reconstruction status: No-Table 1; Source: Table 1; Extraction note: Final PCR classification incorporates the prespecified inhibitor repeat.
D095 OMD-2010-Brillowska-Routine [12]	Routine suspected tinea unguium; Unit: Nail specimen	Optimized 5-hour multiplex PCR; Target: Dermatophyte; Comparator: Direct microscopy; Fungal elements	Reconstruction status: Yes-summed Table 1 strata; Source: Table 1; Extraction note: Microscopy positivity 32; PCR positivity 37.
D096 OMD-2010-Brillowska-Routine [12]	Routine suspected tinea unguium; Unit: Nail specimen	Optimized 5-hour multiplex PCR; Target: Dermatophyte; Comparator: Conventional composite; Positive microscopy and/or culture	Reconstruction status: Yes-summed Table 1 strata; Source: Table 1; Extraction note: Five PCR+/conventional- samples were all *T. rubrum* signals.
D097 OMD-2010-Kondori [13]	Suspected onychomycosis nails; Unit: Nail specimen	Dermatophyte-PCR kit; Target: Dermatophyte; Comparator: Culture; Dermatophyte culture	Reconstruction status: No-Table 1; Source: Table 1; Extraction note: Culture-positive dermatophytes n=60; PCR-positive n=78.
D098 OMD-2010-Kondori [13]	Suspected onychomycosis nails; Unit: Nail specimen	Dermatophyte-PCR kit; Target: Dermatophyte; Comparator: Fluorescence microscopy; Fungal elements	Reconstruction status: No-Table 1; Source: Table 1; Extraction note: PCR sensitivity/specificity against microscopy reported as 85%/91%.
D099 OMD-2014-Dubljanin [24]	All suspected toenail OM; Unit: Patient/toenail	In-house dermatophyte PCR panel; Target: Dermatophyte; Comparator: Culture; Any positive fungal culture	Reconstruction status: Yes-exact inclusion-exclusion; Source: Table 1; Extraction note: P∩culture = 98 + 48 − 101 = 45.
D100 OMD-2014-Dubljanin [24]	All suspected toenail OM; Unit: Patient/toenail	In-house dermatophyte PCR panel; Target: Dermatophyte; Comparator: Microscopy; Fungal elements	Reconstruction status: Yes-exact inclusion-exclusion; Source: Table 1; Extraction note: P∩microscopy = 98 + 84 − 106 = 76.
D101 OMD-2014-Dubljanin [24]	All suspected toenail OM; Unit: Patient/toenail	In-house dermatophyte PCR panel; Target: Dermatophyte; Comparator: Conventional composite; Positive microscopy and/or culture	Reconstruction status: Yes-exact set algebra; Source: Table 1; Extraction note: Table totals imply PCR∩conventional union=79; narrative overlap counts are internally inconsistent and were not substituted.
D102 OMD-2026-Caza [33]	All residual clinical nail specimens; Unit: Nail specimen	Seegene Novaplex Dermatophyte multiplex RT-PCR; Target: Any on-panel target; Comparator: Culture; Any cultured on-panel PCR target	Reconstruction status: Yes-culture-positive margin, three false negatives, 37 unique discordant specimens and reported 75.2% agreement; Source: Table 1, Table 3, Results and Discussion; Extraction note: Target-table false positives total 36 because two specimens had multiple discordant PCR targets; the specimen-level PCR+/culture− count is 34.
D103 OMD-2026-Caza [33]	All residual clinical nail specimens; Unit: Nail specimen	Seegene Novaplex Dermatophyte multiplex RT-PCR; Target: Any on-panel target; Comparator: Conventional composite; Positive KOH and/or on-panel culture	Reconstruction status: Yes-reported 96.0% specimen agreement plus published margins; Source: Table 1, Table 4 and Discussion; Extraction note: Five target-level PCR+/composite− determinations correspond to three unique specimens because of multiple PCR targets.
D104 OMD-2026-Caza [33]	Six assay targets across 149 clinical nails; Unit: Target determination	Seegene Novaplex Dermatophyte multiplex RT-PCR; Target: Any on-panel target; Comparator: Culture; Target-matched culture status	Reconstruction status: No-Table 3; Source: Table 3; Extraction note: Aggregate of six targets; do not pool with nail-level rows.
D105 OMD-2026-Caza [33]	Six assay targets across 149 clinical nails; Unit: Target determination	Seegene Novaplex Dermatophyte multiplex RT-PCR; Target: Any on-panel target; Comparator: Conventional composite; Target-level culture and/or KOH classification	Reconstruction status: No-Table 4; Source: Table 4; Extraction note: Aggregate of six targets; do not pool with nail-level rows.
D106 OMD-2026-Caza [33]	Six assay targets across 149 clinical nails; Unit: Target determination	Seegene Novaplex Dermatophyte multiplex RT-PCR; Target: Any on-panel target; Comparator: Molecular consensus; Agreement of at least 2 of culture, PCR and sequencing	Reconstruction status: No-Table 5; Source: Table 5; Extraction note: Consensus includes the index PCR and sequencing was restricted to discordant specimens; zero false-negative target determinations reported.
D107 OMD-2025-Tirard-Collet [39]	Nail subgroup; valid PCR and tabulated culture category; Unit: Nail specimen	DendrisKIT DP PCR/DNA chip; Target: Any on-panel dermatophyte or *C. albicans*; Comparator: Culture; Any fungal culture-positive nail, including off-panel NDM/non-*C. albicans*	Reconstruction status: Yes-Table 1 nail strata; Source: Table 1 and Results; Extraction note: The three PCR−/culture+ nails grew off-panel non-dermatophyte/non-*C. albicans *fungi.
D108 OMD-2025-Kharibam [40]	KOH-positive, author-classified dermatophyte stratum; Unit: Nail specimen	Two-species multiplex PCR; Target: *T. rubrum* or *T. mentagrophytes*; Comparator: Culture; Any cultured dermatophyte, including off-panel species and unidentified isolates	Reconstruction status: No-Table 4; Source: Table 4; Extraction note: Culture-positive/PCR-negative cells comprise 13 other dermatophytes and five unidentified isolates.
D109 OMD-2020-Gill [38]	Dystrophic fungal nail cohort; Unit: Nail specimen	Arbitrarily primed PCR; Target: Dermatophyte band pattern; Comparator: Culture; Any fungal culture	Reconstruction status: No-Table 2; Source: Table 2; Extraction note: Two non-dermatophyte *A. flavus* cultures were reported PCR-negative; table provides complete method-pattern rows.
D110 OMD-2008-Gupta-Treated [70]	Patients receiving antifungal therapy; Unit: Nail specimen	Double-round *T. rubrum* PCR; Target: *T. rubrum* DNA; Comparator: Calcofluor white microscopy; Fungal hyphae in nail material	Reconstruction status: No-combination table; Source: Full text results table; Extraction note: All participants were undergoing antifungal therapy.
D111 OMD-2008-Gupta-Treated [70]	Patients receiving antifungal therapy; Unit: Nail specimen	Double-round *T. rubrum* PCR; Target: *T. rubrum* DNA; Comparator: Culture; Dermatophyte culture	Reconstruction status: No-combination table; Source: Full text results table; Extraction note: Zero reverse-discordant cell retained without continuity correction.
D112 OMD-2014-Iglesias-Sanchez [71]	Suspected onychomycosis; sufficient material for both tests; Unit: Nail specimen	Nested pan-dermatophyte/*T. rubrum* PCR; Target: Dermatophyte DNA; Comparator: Culture; Dermatophyte growth and morphologic identification	Reconstruction status: No-Table 1; Source: Full text Table 1; Extraction note: Seven initial specimens had insufficient material for PCR and were excluded from paired analysis.
D113 OMD-2015-Spiliopoulou [72]	Routine dermatology outpatients; Unit: Nail specimen	Commercial multiplex dermatophyte PCR; Target: Any targeted dermatophyte; Comparator: Culture; Dermatophyte culture	Reconstruction status: Yes-eight-pattern combination table; Source: Full text combination table; Extraction note: Cell totals reproduce PCR positivity 126 and culture positivity 44.
D114 OMD-2015-Spiliopoulou [72]	Routine dermatology outpatients; Unit: Nail specimen	Commercial multiplex dermatophyte PCR; Target: Any targeted dermatophyte; Comparator: Direct microscopy; Any fungal structures	Reconstruction status: Yes-eight-pattern combination table; Source: Full text combination table; Extraction note: Cell totals reproduce PCR positivity 126 and microscopy positivity 63.
D115 OMD-2023-Navarro-Perez-Culture [73]	Suspected onychomycosis with both tests; Unit: Patient/nail sample	Dermatophyte PCR; Target: Dermatophyte positive/negative; Comparator: Culture; Any fungal culture-positive result	Reconstruction status: No-Table 3; Source: Full text Table 3; Extraction note: Cells sum to PCR 63 and culture 40.
D116 OMD-2020-Mohtady [74]	Suspected onychomycosis; Unit: Patient/nail sample	Conventional dermatophyte PCR; Target: Dermatophyte DNA; Comparator: Culture; Dermatophyte culture	Reconstruction status: No-diagnostic-validity cross-table; Source: Full text cross-table; Extraction note: The coherent cross-table is used; conflicting marginal wording elsewhere is flagged.
D117 OMD-2016-Emam [77]	Clinically suspected dermatophyte onychomycosis; Unit: Patient/nail sample	Real-time PCR; Target: Dermatophyte DNA; Comparator: Culture; Dermatophyte culture-positive	Reconstruction status: Yes-exact marginals plus sensitivity/specificity; Source: Full text tables and Results; Extraction note: Reconstruction yields PCR positivity 58, culture positivity 30, sensitivity 93.3% and specificity 40%.
D118 OMD-2016-Emam [77]	Clinically suspected dermatophyte onychomycosis; Unit: Patient/nail sample	Nested PCR; Target: Dermatophyte DNA; Comparator: Culture; Dermatophyte culture-positive	Reconstruction status: Yes-exact marginals plus sensitivity/specificity; Source: Full text tables and Results; Extraction note: Reconstruction yields PCR positivity 52, culture positivity 30, sensitivity 73.3% and specificity 40%.
D119 OMD-2011-Li [25]	All clinical nails; Unit: Nail specimen	Triplex PCR; Target: Broad fungal group detection; Comparator: Culture; Any fungal growth	Reconstruction status: No-Table 1; Source: Full text Table 1; Extraction note: Comparator-specific cells.
D120 OMD-2011-Li [25]	All clinical nails; Unit: Nail specimen	Triplex PCR; Target: Broad fungal group detection; Comparator: Direct microscopy; Fungal elements	Reconstruction status: No-Table 1; Source: Full text Table 1; Extraction note: Study composite not used.
D121 OMD-2004-Arca [79]	Suspected onychomycosis; Unit: Nail specimen	Direct fungal PCR; Target: Fungal DNA; Comparator: Culture; Any fungal culture	Reconstruction status: Yes-reported conditional counts; Source: Full text Results; Extraction note: Cells reproduce PCR 20 and culture 12.
D122 OMD-2004-Arca [79]	Suspected onychomycosis; Unit: Nail specimen	Direct fungal PCR; Target: Fungal DNA; Comparator: Direct microscopy; Fungal elements	Reconstruction status: Yes-reported conditional counts; Source: Full text Results; Extraction note: Cells reproduce PCR 20 and microscopy 40.
D123 OMD-2022-Trave [82]	Real-life suspected onychomycosis; Unit: Nail specimen	EUROArray Dermatomycosis; Target: Any targeted fungus; Comparator: Culture; Any fungal culture	Reconstruction status: Yes-Table 1 combination patterns; Source: Full text Table 1; Extraction note: Cells reproduce PCR 49 and culture 33.
D124 OMD-2022-Trave [82]	Real-life suspected onychomycosis; Unit: Nail specimen	EUROArray Dermatomycosis; Target: Any targeted fungus; Comparator: KOH microscopy; Fungal elements	Reconstruction status: Yes-Table 1 combination patterns; Source: Full text Table 1; Extraction note: Cells reproduce microscopy 36.
D125 OMD-2019-Watanabe-LAMP [84]	Microscopy-positive nails; Unit: Nail specimen	LAMP; Target: *T. rubrum* or *T. interdigitale*; Comparator: Culture; Target dermatophyte growth	Reconstruction status: No-Table 4; Source: Full text Table 4; Extraction note: Zero reverse-discordant cell retained.
D126 OMD-2019-Watanabe-LAMP [84]	Microscopy-positive nails; Unit: Nail specimen	LAMP; Target: *T. rubrum* or *T. interdigitale*; Comparator: Direct microscopy; Fungal elements; all positive by selection	Reconstruction status: No-selection counts; Source: Full text Results; Extraction note: Not pooled with unselected cohorts.
D127 OMD-2016-Petinataud [85]	Optimal nail sample; Unit: Nail specimen	Bio Evolution real-time PCR; Target: Dermatophyte DNA; Comparator: Culture; Dermatophyte culture	Reconstruction status: No-Table 4; Source: Full text Table 4; Extraction note: Linked to non-optimal sample from same patient.
D128 OMD-2016-Petinataud [85]	Optimal nail sample; Unit: Nail specimen	Bio Evolution real-time PCR; Target: Dermatophyte DNA; Comparator: Direct examination; Hyphae only	Reconstruction status: No-Table 4; Source: Full text Table 4; Extraction note: Hypha-only rule used.
D129 OMD-2016-Petinataud [85]	Non-optimal nail sample; Unit: Nail specimen	Bio Evolution real-time PCR; Target: Dermatophyte DNA; Comparator: Culture; Dermatophyte culture	Reconstruction status: No-Table 4; Source: Full text Table 4; Extraction note: Linked to optimal sample from same patient.
D130 OMD-2016-Petinataud [85]	Non-optimal nail sample; Unit: Nail specimen	Bio Evolution real-time PCR; Target: Dermatophyte DNA; Comparator: Direct examination; Hyphae only	Reconstruction status: No-Table 4; Source: Full text Table 4; Extraction note: Hypha-only rule used.
D131 OMD-2013-Miyajima [90]	Microscopy-positive clinical nails; Unit: Nail specimen	Pan-fungal real-time PCR; Target: Fungal DNA ≥1,500 copies/reaction; Comparator: Culture; Any fungal growth	Reconstruction status: Yes-patient-level Table 4; Source: Full text Table 4; Extraction note: Healthy controls excluded from clinical discordance cells.
D132 OMD-2013-Miyajima [90]	Microscopy-positive clinical nails; Unit: Nail specimen	Pan-fungal real-time PCR; Target: Fungal DNA ≥1,500 copies/reaction; Comparator: Direct microscopy; Fungal elements; all positive by selection	Reconstruction status: Yes-patient-level Table 4; Source: Full text Table 4; Extraction note: Threshold applied exactly as stated.
D133 OMD-2012-Luk [91]	Suspected onychomycosis; Unit: Nail specimen	Pan-dermatophyte PCR; Target: Dermatophyte DNA; Comparator: Culture; Dermatophyte culture	Reconstruction status: Yes-Table 2; Source: Full text Table 2; Extraction note: Cells reproduce PCR 48 and culture 12.
D134 OMD-2012-Luk [91]	Suspected onychomycosis; Unit: Nail specimen	Pan-dermatophyte PCR; Target: Dermatophyte DNA; Comparator: KOH microscopy; Fungal elements	Reconstruction status: Yes-Table 2; Source: Full text Table 2; Extraction note: Cells reproduce microscopy 35.
D135 OMD-2009-Ebihara [92]	Microscopy-positive nails; Unit: Nail specimen	First-round 28S PCR; Target: Any fungal DNA; Comparator: Direct microscopy; Fungal elements; all positive by selection	Reconstruction status: No-reported totals; Source: Full text Results; Extraction note: No culture comparator performed.
D136 OMD-2009-Ebihara [92]	Microscopy-positive nails; Unit: Nail specimen	Nested 28S PCR; Target: Any fungal DNA; Comparator: Direct microscopy; Fungal elements; all positive by selection	Reconstruction status: No-reported totals; Source: Full text Results; Extraction note: Nested and first-round variants are not independent cohorts.
D137 OMD-2010-Litz [94]	Suspected onychomycosis; Unit: Nail specimen	CHS1 dermatophyte PCR; Target: Dermatophyte DNA; Comparator: Culture; Dermatophyte culture	Reconstruction status: Yes-Table 2; Source: Full text Table 2; Extraction note: Culture categories other than dermatophyte treated comparator-negative.
D138 OMD-2010-Litz [94]	Suspected onychomycosis; Unit: Nail specimen	CHS1 dermatophyte PCR; Target: Dermatophyte DNA; Comparator: PAS histology; Fungal elements	Reconstruction status: Yes-Table 2; Source: Full text Table 2; Extraction note: Exact aggregation across table rows.
D139 OMD-2010-Litz [94]	Suspected onychomycosis; Unit: Nail specimen	CHS1 dermatophyte PCR; Target: Dermatophyte DNA; Comparator: KOH microscopy; Fungal elements	Reconstruction status: Yes-Table 2; Source: Full text Table 2; Extraction note: Exact aggregation across table rows.
D140 OMD-2018-Adel-Taha [95]	Suspected onychomycosis; recent therapy excluded; Unit: Nail specimen	Multiplex dermatophyte PCR; Target: Dermatophyte DNA; Comparator: Culture; Dermatophyte culture	Reconstruction status: Yes-Tables 2–4 plus exact performance; Source: Full text Tables 2–4; Extraction note: Cells reproduce PCR 29, culture 20, sensitivity 90%, specificity 63.3%.
D141 OMD-2018-Adel-Taha [95]	Suspected onychomycosis; recent therapy excluded; Unit: Nail specimen	Multiplex dermatophyte PCR; Target: Dermatophyte DNA; Comparator: KOH microscopy; Fungal elements	Reconstruction status: Yes-microscopy-stratified Results; Source: Full text Results and Table 4; Extraction note: Microscopy-negative section gives three PCR positives.
D142 OMD-2011-Brasch [96]	Routine nail samples; Unit: Nail specimen	*T. rubrum*-specific PCR; Target: *T. rubrum* DNA; Comparator: Culture; *T. rubrum* culture	Reconstruction status: No-exact counts in Results; Source: Full text Results and Figure 3; Extraction note: Other cultured fungi remain comparator-negative for *T. rubrum* endpoint.
D143 OMD-2025-Wang-RPA [99]	Suspected onychomycosis; Unit: Nail specimen	Pan-dermatophyte RPA-LFD; Target: Dermatophyte DNA; Comparator: Culture; Dermatophyte culture	Reconstruction status: Yes-Table 3 plus culture species counts; Source: Full text Table 3; Extraction note: Five non-dermatophyte cultures remain comparator-negative for the dermatophyte endpoint.
D144 OMD-2025-Wang-RPA [99]	Suspected onychomycosis; Unit: Nail specimen	Pan-dermatophyte RPA-LFD; Target: Dermatophyte DNA; Comparator: KOH microscopy; Fungal elements	Reconstruction status: No-direct Table 3 aggregation; Source: Full text Table 3; Extraction note: Microscopy detects fungal elements without dermatophyte speciation.
D145 OMD-2012-Dhib [100]	Suspected onychomycosis; Unit: Nail specimen	CHS1 pan-dermatophyte PCR; Target: Dermatophyte DNA; Comparator: Conventional composite; KOH microscopy positive and/or culture positive/contaminated	Reconstruction status: Yes-Table 2; Source: Full text Table 2; Extraction note: All four non-negative conventional pattern groups define comparator-positive.
D146 OMD-2012-Dhib [100]	Suspected onychomycosis; Unit: Nail specimen	CHS1 pan-dermatophyte PCR; Target: Dermatophyte DNA; Comparator: KOH microscopy; Fungal elements	Reconstruction status: Yes-Table 2; Source: Full text Table 2; Extraction note: Exact aggregation across microscopy strata.
D147 OMD-2012-Dhib [100]	Suspected onychomycosis; contaminated cultures excluded; Unit: Nail specimen	CHS1 pan-dermatophyte PCR; Target: Dermatophyte DNA; Comparator: Culture; Dermatophyte culture; contaminated cultures excluded	Reconstruction status: Yes-Table 2; Source: Full text Table 2; Extraction note: Fifteen contaminated cultures omitted rather than classified negative.
D148 OMD-2019-Koo [101]	Suspected onychomycosis; prior oral therapy excluded; Unit: Nail specimen	Pan-dermatophyte qPCR; Target: Dermatophyte DNA; Comparator: Culture; Dermatophyte culture	Reconstruction status: No-published 2 × 2; Source: Full text Table 3; Extraction note: Published cells against fungal culture.
D149 OMD-2019-Koo [101]	Suspected onychomycosis; prior oral therapy excluded; Unit: Nail specimen	Pan-dermatophyte qPCR; Target: Dermatophyte DNA; Comparator: Expanded standard; Culture plus repeat qPCR and ITS2 sequencing	Reconstruction status: No-published 2 × 2; Source: Full text Table 3; Extraction note: Not pooled with conventional-comparator rows.
D150 OMD-2019-Koo [101]	Suspected onychomycosis; prior oral therapy excluded; Unit: Nail specimen	*T. rubrum* qPCR; Target: *T. rubrum* DNA; Comparator: Culture; *T. rubrum* culture	Reconstruction status: No-published 2 × 2; Source: Full text Table 3; Extraction note: Shares specimens with the pan-dermatophyte row.
D151 OMD-2019-Koo [101]	Suspected onychomycosis; prior oral therapy excluded; Unit: Nail specimen	*Fusarium* qPCR; Target: *Fusarium* DNA; Comparator: Culture; *Fusarium* culture	Reconstruction status: No-published 2 × 2; Source: Full text Table 3; Extraction note: Shares specimens with the pan-dermatophyte row.
D152 OMD-2020-Ross [102]	Prospective nail subgroup; yeast/NDM comparison excluded; Unit: Nail specimen	Commercial multiplex tandem PCR; Target: Dermatophyte DNA; Comparator: Culture; Dermatophyte culture	Reconstruction status: Yes-Table 1; Source: Full text Table 1; Extraction note: Exact aggregation of the nail block.
D153 OMD-2020-Ross [102]	Prospective nail subgroup; yeast/NDM comparison excluded; Unit: Nail specimen	Commercial multiplex tandem PCR; Target: Dermatophyte DNA; Comparator: Microscopy; Dermatophyte-compatible hyphae	Reconstruction status: Yes-Table 1; Source: Full text Table 1; Extraction note: Exact aggregation of the nail block.
D154 OMD-2020-Ross [102]	Prospective nail subgroup; yeast/NDM comparison excluded; Unit: Nail specimen	Commercial multiplex tandem PCR; Target: Dermatophyte DNA; Comparator: Conventional composite; Dermatophyte microscopy positive and/or culture positive	Reconstruction status: Yes-Table 1; Source: Full text Table 1; Extraction note: Exact aggregation of the first three microscopy/culture strata.
D155 OMD-2021-Lin [103]	Part 3 prospective nail subgroup; Unit: Nail specimen	AusDiagnostics multiplex RT-PCR v2; Target: Dermatophyte DNA; Comparator: Culture; Dermatophyte culture	Reconstruction status: Yes-Tables 3 and Figure 1; Source: Full text Table 3, Figure 1 and Results; Extraction note: All culture-positive nails were RT-PCR positive; totals reconstruct remaining cells.
D156 OMD-2021-Lin [103]	Part 3 prospective nail subgroup; Unit: Nail specimen	AusDiagnostics multiplex RT-PCR v2; Target: Dermatophyte DNA; Comparator: Microscopy; Dermatophyte-compatible fungal elements	Reconstruction status: Yes-Figure 1 and Results; Source: Full text Figure 1 and Results; Extraction note: All microscopy-positive nails were RT-PCR positive.
D157 OMD-2021-Lin [103]	Part 3 prospective nail subgroup; Unit: Nail specimen	AusDiagnostics multiplex RT-PCR v2; Target: Dermatophyte DNA; Comparator: Conventional composite; Microscopy positive and/or dermatophyte culture positive	Reconstruction status: Yes-Figure 1 and exact overall totals; Source: Full text Figure 1 and Results; Extraction note: Figure reports 32% conventional positivity in 128 nails; exact union 41 is confirmed by overall totals and 100% RT-PCR sensitivity.
D158 OMD-2021-Lin [103]	Part 2 culture-positive nail validation cohort; Unit: Nail specimen	AusDiagnostics multiplex RT-PCR v2; Target: Dermatophyte DNA; Comparator: Culture; Dermatophyte culture; all positive by selection	Reconstruction status: No-direct Table 2; Source: Full text Table 2; Extraction note: Sensitivity validation stratum is not combined with the prospective cohort.
D159 OMD-2021-Cuchi-Burgos [104]	KOH-enriched workflow cohort; valid qPCR only; Unit: Nail specimen	DermaGenius 2.0 qPCR; Target: Dermatophytes and *C. albicans*; Comparator: KOH microscopy; Fungal elements	Reconstruction status: Yes-Table 1; Source: Full text Table 1; Extraction note: Ten non-amplifiable qPCR results omitted, not recoded negative.
D160 OMD-2021-Cuchi-Burgos [104]	KOH-enriched workflow cohort; valid qPCR only; Unit: Nail specimen	DermaGenius 2.0 qPCR; Target: Dermatophytes and *C. albicans*; Comparator: Culture; Dermatophyte culture	Reconstruction status: Yes-Table 1; Source: Full text Table 1; Extraction note: Molecular endpoint includes *C. albicans*; comparator is dermatophyte culture. Ten invalid qPCR results excluded.
D161 OMD-2026-Sharma [28]	Clinically suspected onychomycosis; Unit: Nail specimen	Commercial two-tube real-time PCR; Target: Panel-covered dermatophyte DNA; Comparator: Culture; Dermatophyte culture	Reconstruction status: No-direct Table 1; Source: Full text Table 1; Extraction note: One culture-positive *T. schoenleinii* was outside the PCR panel.
D162 OMD-2026-Sharma [28]	Clinically suspected onychomycosis; Unit: Nail specimen	Commercial two-tube real-time PCR; Target: Panel-covered dermatophyte DNA; Comparator: KOH microscopy; Dermatophyte-compatible fungal elements	Reconstruction status: No-direct Table 1; Source: Full text Table 1; Extraction note: Cells reproduce PCR 43 and KOH 55.
D163 OMD-2004-Menotti [9]	Series 1; optimally sampled nail divided between methods; Unit: Patient/nail	PCR-RFLP; Target: Dermatophytes or Scytalidium DNA; Comparator: Conventional composite; Black chlorazol microscopy positive and/or fungal culture positive	Reconstruction status: No-published 2×2 table; Source: Full text Table 1; Extraction note: Published cells; reported sensitivity 93% and specificity 94% are reproduced.
D164 OMD-2004-Menotti [9]	Series 2; non-optimal distal clipping for PCR versus optimal sample from same nail for mycology; Unit: Patient/nail	PCR-RFLP; Target: Dermatophytes or Scytalidium DNA; Comparator: Conventional composite; Black chlorazol microscopy positive and/or fungal culture positive on optimally collected sample	Reconstruction status: No-published 2×2 table; Source: Full text Table 1; Extraction note: Published cells; reported sensitivity 93% and specificity 84% are reproduced.
D165 OMD-2007-Gupta-DoublePCR [10]	Prior *T. rubrum* culture-positive cohort; mixed treatment exposure; Unit: Nail specimen	Actin double-round PCR; Target: *T. rubrum* DNA; Comparator: Culture; Contemporaneous *T. rubrum* culture	Reconstruction status: Yes-reconstructed from complete 12-pattern table; Source: Full text Table 2; Extraction note: At least 15 actin-PCR-positive/culture-negative samples were collected during antifungal treatment.
D166 OMD-2007-Gupta-DoublePCR [10]	Prior *T. rubrum* culture-positive cohort; mixed treatment exposure; Unit: Nail specimen	ITS1 double-round PCR; Target: *T. rubrum* DNA; Comparator: Culture; Contemporaneous *T. rubrum* culture	Reconstruction status: Yes-reconstructed from complete 12-pattern table; Source: Full text Table 2; Extraction note: ITS1 assay result kept separate from actin and combined endpoints.
D167 OMD-2007-Gupta-DoublePCR [10]	Prior *T. rubrum* culture-positive cohort; mixed treatment exposure; Unit: Nail specimen	Combined actin-or-ITS1 double-round PCR; Target: *T. rubrum* DNA detected by either assay; Comparator: Culture; Contemporaneous *T. rubrum* culture	Reconstruction status: Yes-reconstructed from complete 12-pattern table; Source: Full text Table 2; Extraction note: Primary molecular endpoint for this report; no duplicate counting of assay-variant rows.
D168 OMD-2007-Gupta-DoublePCR [10]	Prior *T. rubrum* culture-positive cohort; mixed treatment exposure; Unit: Nail specimen	Actin double-round PCR; Target: *T. rubrum* DNA; Comparator: KOH microscopy; Fungal elements	Reconstruction status: Yes-reconstructed from complete 12-pattern table; Source: Full text Table 2; Extraction note: KOH detects fungal elements without species attribution.
D169 OMD-2007-Gupta-DoublePCR [10]	Prior *T. rubrum* culture-positive cohort; mixed treatment exposure; Unit: Nail specimen	ITS1 double-round PCR; Target: *T. rubrum* DNA; Comparator: KOH microscopy; Fungal elements	Reconstruction status: Yes-reconstructed from complete 12-pattern table; Source: Full text Table 2; Extraction note: KOH detects fungal elements without species attribution.
D170 OMD-2007-Gupta-DoublePCR [10]	Prior *T. rubrum* culture-positive cohort; mixed treatment exposure; Unit: Nail specimen	Combined actin-or-ITS1 double-round PCR; Target: *T. rubrum* DNA detected by either assay; Comparator: KOH microscopy; Fungal elements	Reconstruction status: Yes-reconstructed from complete 12-pattern table; Source: Full text Table 2; Extraction note: Primary combined molecular endpoint against microscopy.
D171 OMD-2024-Felek [8]	37 mycology-positive patients selected from 46 suspected cases; 21/37 previously treated; Unit: Patient-linked nail specimen	EUROArray Dermatomycosis PCR-based microarray on primary nail material; Target: Any fungal detection; Comparator: Culture; Any fungal detection by culture; organism coverage differs from molecular panel	Reconstruction status: Yes-aggregated from patient-level results in Table 2; Source: Table 2, printed p. 1133; Methods p. 1132; Results p. 1134; Extraction note: Abstract reports 14 culture-positive specimens; Table 2 and Results p. 1134 establish 15 (14 single-organism plus one dual-organism culture). Conclusion 65.2% culture-negative does not match the displayed cohort. Table 2 governs extracted cells. Nine patients not included in the displayed cohort are not imputed as double negative. Four endpoint rows share the same patients and must not be combined. Species disagreement is not a negative any-fungus result.
D172 OMD-2024-Felek [8]	37 mycology-positive patients selected from 46 suspected cases; 21/37 previously treated; Unit: Patient-linked nail specimen	EUROArray Dermatomycosis PCR-based microarray on primary nail material; Target: Dermatophyte detection; Comparator: Culture; Dermatophyte detection by culture; organism coverage differs from molecular panel	Reconstruction status: Yes-aggregated from patient-level results in Table 2; Source: Table 2, printed p. 1133; Methods p. 1132; Results p. 1134; Extraction note: Abstract reports 14 culture-positive specimens; Table 2 and Results p. 1134 establish 15 (14 single-organism plus one dual-organism culture). Conclusion 65.2% culture-negative does not match the displayed cohort. Table 2 governs extracted cells. Nine patients not included in the displayed cohort are not imputed as double negative. Four endpoint rows share the same patients and must not be combined. Species disagreement is not a negative any-fungus result.
D173 OMD-2024-Felek [8]	37 mycology-positive patients selected from 46 suspected cases; 21/37 previously treated; Unit: Patient-linked nail specimen	EUROArray Dermatomycosis PCR-based microarray on primary nail material; Target: Candida detection; Comparator: Culture; Candida detection by culture; organism coverage differs from molecular panel	Reconstruction status: Yes-aggregated from patient-level results in Table 2; Source: Table 2, printed p. 1133; Methods p. 1132; Results p. 1134; Extraction note: Abstract reports 14 culture-positive specimens; Table 2 and Results p. 1134 establish 15 (14 single-organism plus one dual-organism culture). Conclusion 65.2% culture-negative does not match the displayed cohort. Table 2 governs extracted cells. Nine patients not included in the displayed cohort are not imputed as double negative. Four endpoint rows share the same patients and must not be combined. Species disagreement is not a negative any-fungus result.
D174 OMD-2024-Felek [8]	37 mycology-positive patients selected from 46 suspected cases; 21/37 previously treated; Unit: Patient-linked nail specimen	EUROArray Dermatomycosis PCR-based microarray on primary nail material; Target: Non-dermatophyte mold detection; Comparator: Culture; Non-dermatophyte mold detection by culture; organism coverage differs from molecular panel	Reconstruction status: Yes-aggregated from patient-level results in Table 2; Source: Table 2, printed p. 1133; Methods p. 1132; Results p. 1134; Extraction note: Abstract reports 14 culture-positive specimens; Table 2 and Results p. 1134 establish 15 (14 single-organism plus one dual-organism culture). Conclusion 65.2% culture-negative does not match the displayed cohort. Table 2 governs extracted cells. Nine patients not included in the displayed cohort are not imputed as double negative. Four endpoint rows share the same patients and must not be combined. Species disagreement is not a negative any-fungus result.
D089V OMD-2019-Hayette [19]	Archived nail clippings; valid PCR results only (revision derivation); Unit: Nail specimen	DermaGenius Nail RT-PCR; Target: Dermatophyte or *C. albicans*; Comparator: Culture; Dermatophyte or *C. albicans* culture	Reconstruction status: Yes-Table 2 plus Inhibition and Discordance with culture results paragraphs (p. 280); Source: Hayette2019, Table 2 and p. 280: eight inhibited PCR; two target-culture positive and six target-culture negative.; Extraction note: Original D089 = 60/27/8/43; N = 138. Exclude eight inhibited PCR results: subtract 2 from M−/C+ and 6 from M−/C−. D089V = 60/27/6/37; N = 130. This is an alternative derivation, not additional independent evidence.

**Table 11:** All paired cells and net detection differences with conditional uncertainty intervals. a = M+/C+; b = M+/C-; c = M-/C+; d = M-/C-; N = a + b + c + d. Gross molecular-only detection is 100b/N, and the net difference is 100(b - c)/N. For a count x, let Lx and Ux be the equal-tailed 97.5% Clopper-Pearson limits for x/N: Lx = BetaQuantile(0.0125; x, N - x + 1) when x > 0 (otherwise zero), and Ux = BetaQuantile(0.9875; x + 1, N - x) when x < N (otherwise one). Net limits are 100(Lb - Uc) and 100(Ub - Lc). The union bound guarantees at least 95% per-comparison coverage under independent sampling of paired observations; this is not simultaneous coverage of all rows [7]. Source error, clustering, and reconstruction uncertainty are not represented. D104-D106 aggregate correlated targets per nail and have no intervals. D089V is an alternative, not an additional cohort.

Row	N	a	b	c	d	Gross M-only, %	Net, pp	Conditional 95% CI, pp
D001	346	57	67	15	207	19.36	15.03	7.34 to 22.35
D002	41223	21290	2796	2122	15015	6.78	1.64	1.11 to 2.16
D003	151	65	15	28	43	9.93	-8.61	-21.51 to 4.77
D004	151	67	13	0	71	8.61	8.61	1.38 to 15.13
D005	139	79	19	2	39	13.67	12.23	2.12 to 21.42
D006	103	30	8	21	44	7.77	-12.62	-27.78 to 3.57
D007	51	23	7	12	9	13.73	-9.8	-34.51 to 16.48
D008	204	78	24	9	93	11.76	7.35	-1.60 to 15.97
D009	425	168	116	35	106	27.29	19.06	10.83 to 26.93
D010	2452	1159	79	290	924	3.22	-8.61	-10.89 to -6.29
D011	2452	638	600	280	934	24.47	13.05	9.61 to 16.46
D012	152	38	83	0	31	54.61	54.61	42.36 to 63.77
D013	152	81	40	15	16	26.32	16.45	2.01 to 30.01
D014	83	0	83	0	0	100	100	89.71 to 100.00
D015	69	38	0	0	31	0	0	-6.15 to 6.15
D016	118	23	27	4	64	22.88	19.49	5.51 to 32.01
D017	195	43	113	0	39	57.95	57.95	47.46 to 65.91
D018	195	151	5	30	9	2.56	-12.82	-21.39 to -3.63
D019	316	191	51	6	68	16.14	14.24	7.33 to 20.74
D020	316	216	26	24	50	8.23	0.63	-6.50 to 7.75
D021	1148	142	587	108	311	51.13	41.72	36.27 to 46.91
D022	624	379	171	27	47	27.4	23.08	16.94 to 28.92
D023	67	8	22	3	34	32.84	28.36	6.70 to 46.41
D024	67	21	9	5	32	13.43	5.97	-12.35 to 23.48
D025	67	22	8	8	29	11.94	0	-19.05 to 19.05
D026	306	44	87	1	174	28.43	28.1	20.72 to 34.61
D027	306	20	18	8	260	5.88	3.27	-2.22 to 8.64
D028	182	94	22	5	61	12.09	9.34	0.36 to 17.82
D029	182	116	0	7	59	0	-3.85	-8.38 to 1.03
D030	236	83	30	23	100	12.71	2.97	-6.68 to 12.51
D031	236	89	42	17	88	17.8	10.59	0.65 to 20.16
D032	236	103	10	48	75	4.24	-16.1	-25.04 to -6.58
D033	75	49	6	4	16	8	2.67	-11.77 to 16.74
D034	75	53	6	0	16	8	8	-3.12 to 17.93
D035	75	55	0	10	10	0	-13.33	-24.62 to -0.22
D036	127	34	32	0	61	25.2	25.2	13.62 to 34.88
D037	127	34	38	0	55	29.92	29.92	17.74 to 39.92
D038	127	31	35	2	59	27.56	25.98	12.82 to 37.28
D039	125	27	33	27	38	26.4	4.8	-13.05 to 22.35
D040	106	29	14	12	51	13.21	1.89	-13.31 to 16.93
D041	106	43	0	12	51	0	-11.32	-20.09 to -1.38
D042	106	54	0	1	51	0	-0.94	-5.87 to 4.04
D043	13	4	9	0	0	69.23	69.23	6.36 to 92.59
D044	7	0	7	0	0	100	100	6.94 to 100.00
D045	6	0	6	0	0	100	100	-3.65 to 100.00
D046	14	14	0	0	0	0	0	-26.88 to 26.88
D047	8	7	1	0	0	12.5	12.5	-42.02 to 57.55
D048	7	6	1	0	0	14.29	14.29	-46.35 to 62.87
D049	35	24	5	4	2	14.29	2.86	-24.99 to 29.99
D050	32	10	11	0	11	34.38	34.38	4.05 to 55.65
D051	32	19	2	0	11	6.25	6.25	-12.27 to 23.07
D052	81	42	16	0	23	19.75	19.75	5.56 to 31.58
D053	81	44	14	17	6	17.28	-3.7	-24.01 to 16.97
D054	19	3	3	0	13	15.79	15.79	-17.99 to 42.87
D055	19	3	1	0	15	5.26	5.26	-20.53 to 29.20
D056	106	36	65	0	5	61.32	61.32	45.95 to 71.82
D057	106	36	62	0	8	58.49	58.49	43.10 to 69.22
D058	512	323	0	26	163	0	-5.08	-7.70 to -2.29
D059	512	60	38	67	347	7.42	-5.66	-11.75 to 0.51
D060	137	35	20	7	75	14.6	9.49	-2.53 to 20.88
D061	109	43	8	14	44	7.34	-5.5	-18.94 to 8.38
D062	109	26	25	1	57	22.94	22.02	8.81 to 33.25
D063	109	51	0	14	44	0	-12.84	-21.77 to -2.65
D064	52	26	0	12	14	0	-23.08	-38.79 to -3.31
D065	57	25	0	2	30	0	-3.51	-13.51 to 7.10
D066	252	54	32	2	164	12.7	11.9	5.20 to 18.09
D067	252	66	20	13	153	7.94	2.78	-4.63 to 10.08
D068	52	7	23	0	22	44.23	44.23	20.68 to 60.56
D069	42	1	19	0	22	45.24	45.24	18.06 to 63.38
D070	42	3	17	2	20	40.48	35.71	5.88 to 58.43
D071	100	70	28	0	2	28	28	14.15 to 39.26
D072	49	8	11	0	30	22.45	22.45	2.09 to 38.63
D073	107	33	44	5	25	41.12	36.45	18.97 to 51.13
D074	107	37	40	20	10	37.38	18.69	-1.61 to 37.62
D075	107	77	0	30	0	0	-28.04	-38.89 to -14.75
D076	70	18	24	2	26	34.29	31.43	10.90 to 48.05
D077	70	31	11	10	18	15.71	1.43	-18.93 to 21.62
D078	70	18	22	20	10	31.43	2.86	-22.73 to 28.12
D079	70	14	6	24	26	8.57	-25.71	-45.55 to -2.88
D080	28	24	0	4	0	0	-14.29	-35.32 to 11.22
D081	28	1	23	0	4	82.14	82.14	45.94 to 94.92
D082	28	10	14	0	4	50	50	13.86 to 71.65
D083	201	132	63	0	6	31.34	31.34	22.01 to 39.23
D084	201	151	44	0	6	21.89	21.89	13.53 to 29.17
D085	165	45	33	2	85	20	18.79	8.64 to 27.83
D086	165	64	14	8	79	8.48	3.64	-5.72 to 12.78
D087	203	68	9	41	85	4.43	-15.76	-25.47 to -5.39
D088	138	63	24	14	37	17.39	7.25	-6.62 to 20.67
D089	138	60	27	8	43	19.57	13.77	0.62 to 26.06
D090	138	76	11	19	32	7.97	-5.8	-18.04 to 6.82
D091	90	67	8	5	10	8.89	3.33	-10.14 to 16.44
D092	90	20	55	0	15	61.11	61.11	44.02 to 72.50
D093	131	33	29	0	69	22.14	22.14	11.24 to 31.39
D094	109	19	18	3	69	16.51	13.76	0.69 to 25.62
D095	109	28	9	4	68	8.26	4.59	-6.60 to 15.33
D096	109	32	5	5	67	4.59	0	-10.04 to 10.04
D097	177	57	21	3	96	11.86	10.17	1.59 to 18.14
D098	177	69	9	12	87	5.08	-1.69	-10.19 to 6.90
D099	183	45	53	3	82	28.96	27.32	16.45 to 36.87
D100	183	76	22	8	77	12.02	7.65	-1.88 to 16.80
D101	183	79	19	11	74	10.38	4.37	-5.28 to 13.81
D102	149	59	34	3	53	22.82	20.81	9.16 to 31.17
D103	149	90	3	3	53	2.01	0	-6.07 to 6.07
D104	894	59	36	3	796	4.03	3.69	Not assigned
D105	894	90	5	3	796	0.56	0.22	Not assigned
D106	894	87	8	0	799	0.89	0.89	Not assigned
D107	25	10	6	3	6	24	12	-25.99 to 46.02
D108	70	47	3	18	2	4.29	-21.43	-38.55 to -1.62
D109	48	38	6	4	0	12.5	4.17	-17.73 to 25.27
D110	83	8	13	18	44	15.66	-6.02	-25.74 to 14.28
D111	83	2	19	0	62	22.89	22.89	8.25 to 34.92
D112	225	57	16	33	119	7.11	-7.56	-16.97 to 2.14
D113	418	38	88	6	286	21.05	19.62	13.37 to 25.44
D114	418	58	68	5	287	16.27	15.07	9.39 to 20.41
D115	121	32	31	8	50	25.62	19.01	3.65 to 33.05
D116	61	37	4	0	20	6.56	6.56	-5.46 to 17.41
D117	80	28	30	2	20	37.5	35	15.82 to 50.40
D118	80	22	30	8	20	37.5	27.5	5.50 to 46.73
D119	104	29	13	0	62	12.5	12.5	2.09 to 21.61
D120	104	42	0	11	51	0	-10.58	-19.28 to -0.74
D121	52	6	14	6	26	26.92	15.38	-10.93 to 39.26
D122	52	16	4	24	8	7.69	-38.46	-60.66 to -10.27
D123	100	27	22	6	45	22	16	-0.21 to 30.84
D124	100	27	22	9	42	22	13	-4.10 to 29.02
D125	90	59	27	0	4	30	30	14.90 to 42.05
D126	90	86	0	4	0	0	-4.44	-12.03 to 3.76
D127	90	21	10	0	59	11.11	11.11	0.13 to 20.75
D128	90	30	1	19	40	1.11	-20	-32.40 to -5.42
D129	90	15	16	0	59	17.78	17.78	4.95 to 28.64
D130	90	29	2	15	44	2.22	-14.44	-27.18 to -0.14
D131	32	13	15	1	3	46.88	43.75	8.64 to 67.48
D132	32	28	0	4	0	0	-12.5	-31.42 to 9.95
D133	120	10	38	2	70	31.67	30	15.84 to 41.93
D134	120	19	29	16	56	24.17	10.83	-5.93 to 26.87
D135	50	35	0	15	0	0	-30	-46.61 to -8.08
D136	50	47	0	3	0	0	-6	-18.21 to 7.43
D137	550	91	112	23	324	20.36	16.18	10.12 to 22.01
D138	550	170	33	126	221	6	-16.91	-23.25 to -10.32
D139	550	138	65	84	263	11.82	-3.45	-10.13 to 3.27
D140	50	18	11	2	19	22	18	-4.87 to 37.60
D141	50	24	5	11	10	10	-12	-35.16 to 13.19
D142	230	40	47	8	135	20.43	16.96	7.53 to 25.74
D143	190	66	32	6	86	16.84	13.68	3.88 to 22.83
D144	190	85	13	9	83	6.84	2.11	-6.08 to 10.18
D145	201	150	32	13	6	15.92	9.45	-0.90 to 19.41
D146	201	141	41	10	9	20.4	15.42	4.80 to 25.39
D147	186	122	46	10	8	24.73	19.35	7.61 to 30.23
D148	204	19	41	1	143	20.1	19.61	11.08 to 27.15
D149	204	48	12	1	143	5.88	5.39	-0.30 to 10.69
D150	204	8	21	0	175	10.29	10.29	3.92 to 16.06
D151	204	19	6	3	176	2.94	1.47	-3.77 to 6.59
D152	156	35	52	2	67	33.33	32.05	19.94 to 42.33
D153	156	73	14	11	58	8.97	1.92	-8.51 to 12.24
D154	156	76	11	12	57	7.05	-0.64	-10.66 to 9.41
D155	128	24	48	0	56	37.5	37.5	24.65 to 47.74
D156	128	33	39	0	56	30.47	30.47	18.29 to 40.46
D157	128	41	31	0	56	24.22	24.22	12.84 to 33.78
D158	47	43	0	4	0	0	-8.51	-22.20 to 6.99
D159	142	81	3	30	28	2.11	-19.01	-29.54 to -7.25
D160	142	52	32	4	54	22.54	19.72	7.40 to 30.79
D161	65	5	38	3	19	58.46	53.85	29.62 to 71.34
D162	65	40	3	15	7	4.62	-18.46	-36.17 to 1.77
D163	48	28	1	2	17	2.08	-2.08	-15.85 to 12.22
D164	88	64	3	5	16	3.41	-2.27	-13.33 to 9.09
D165	62	10	27	4	21	43.55	37.1	12.26 to 57.04
D166	62	7	21	7	27	33.87	22.58	-2.54 to 44.75
D167	62	11	32	3	16	51.61	46.77	21.99 to 65.39
D168	62	30	7	18	7	11.29	-17.74	-39.70 to 6.55
D169	62	20	8	28	6	12.9	-32.26	-55.00 to -5.39
D170	62	34	9	14	5	14.52	-8.06	-30.64 to 15.58
D171	37	15	22	0	0	59.46	59.46	28.70 to 77.09
D172	37	7	26	0	4	70.27	70.27	39.52 to 85.62
D173	37	3	1	2	31	2.7	-2.7	-20.18 to 15.59
D174	37	1	12	3	21	32.43	24.32	-7.60 to 50.79
D089V	130	60	27	6	37	20.77	16.15	2.77 to 28.46

Appendix 3

Individual Methodological Appraisal

Table 12 gives all 97 report-level judgments and their reasons. S = population selection; I = index interpretation; C = comparator interpretation; F = flow and pairing; A = clinical applicability. High/low denote concern in that domain, not a global quality ranking. Unclear means that the available assessment record was insufficient. In particular, all I judgments are unclear because neither prospectively fixed thresholds nor blinded interpretation was established by this audit; this does not assert that all source papers omitted those methods. The appraisal is based on 96 existing full-text extractions, with 21 targeted source rechecks and one newly retrieved full-text assessment, and is not an independent duplicate QUADAS-2 assessment.

**Table 12:** Report-level domain judgments with supporting grounds and provenance.

Report	S / I / C / F / A	Selection and index grounds	Comparator, flow and applicability grounds	Assessment basis and overlap
OMD-2006-Walberg [11]	Unclear / Unclear / Low / Unclear / Low	S: Recruitment sequence, consecutive enrollment and the complete exclusion process are not established by the available extraction. Clinically suspected onychomycosis; 10 healthy controls reported separately. I: Prospective threshold selection and blinded interpretation are not established by the available assessment record. Molecular endpoint: Broad fungal amplification; primary binary analysis restricted to dermatophyte detection.	C: For raw paired discordance, the comparator is interpreted as a separate detection method rather than disease truth. No infallible gold standard; sequence plus culture comparison. F: Completeness of valid, same-specimen paired observations is not fully established for all reported analyses. Eight binary-positive pairs are species/genus-discordant; some broad fungal amplicons were unsequenced or counted outside dermatophyte endpoint. A: Clinical nail comparison applicable within its stated organism target and setting; it does not establish all-pathogen accuracy or clinical benefit. Norway; tertiary dermatology/mycology	Workbook v0.12 Evidence_Table row 5; Full_Text_Screening record OMD-2006-Walberg; source locator: Full text Table I; Results. Existing full-text extraction only; source PDF not re-examined for this appraisal. Overlap: None identified
OMD-2025-Ivagnes [48]	Unclear / Unclear / Low / Unclear / High	S: Recruitment sequence, consecutive enrollment and the complete exclusion process are not established by the available extraction. 120 mixed dermatomycosis specimens: 78 nails, 35 skin scrapings, 7 other. I: Prospective threshold selection and blinded interpretation are not established by the available assessment record. Molecular endpoint: Commercial multiplex panel; 56 fungal targets.	C: For raw paired discordance, the comparator is interpreted as a separate detection method rather than disease truth. Culture and composite conventional reference; discordances tested by panfungal PCR. F: Completeness of valid, same-specimen paired observations is not fully established for all reported analyses. Mixed skin/nail cohort and small pilot; adjusted analysis excludes off-panel organisms. A: Restricted applicability to unselected, first-line suspected onychomycosis: Mixed cohort-nail subgroup not cross-tabulated. 120 mixed dermatomycosis specimens: 78 nails, 35 skin scrapings, 7 other	Workbook v0.12 Evidence_Table row 6; Full_Text_Screening record OMD-2025-Ivagnes; source locator: Full text Tables 1-2; Discussion. Existing full-text extraction only; source PDF not re-examined for this appraisal. Overlap: None identified
OMD-2024-Gupta-US [49]	Unclear / Unclear / Low / High / Unclear	S: Recruitment sequence, consecutive enrollment and the complete exclusion process are not established by the available extraction. Abnormal-appearing toenail specimens submitted in 2022-2023. I: Prospective threshold selection and blinded interpretation are not established by the available assessment record. Molecular endpoint: Dermatophytes, yeasts and NDM panel.	C: For raw paired discordance, the comparator is interpreted as a separate detection method rather than disease truth. Histopathology comparator, not assumed infallible. F: Paired PCR/histology analysis is a test-order-selected subset of a larger laboratory database; the article contains a competing denominator outside the exact figure. A: Applicability depends on treatment, sampling, target or subgroup qualifications: Retrospective test-order selection; internal text denominator of 41,284 conflicts with exact Figure 4 total of 41,223.	Workbook v0.12 Evidence_Table row 7; Full_Text_Screening record OMD-2024-Gupta-US; source locator: Full text Figure 4; Results §3.4. Existing full-text extraction only; source PDF not re-examined for this appraisal. Overlap: Potential Bako assay/institutional linkage; check patient/time overlap before multi-study pooling
OMD-2023-Marin-Maldonado [50]	High / Unclear / Low / High / High	S: Only the high/moderate sequencing-quality subset contributes paired estimates; sequencing quality was associated with molecular positivity. I: Prospective threshold selection and blinded interpretation are not established by the available assessment record. Molecular endpoint: *T. rubrum* plus pan-dermatophyte.	C: For raw paired discordance, the comparator is interpreted as a separate detection method rather than disease truth. High/moderate-quality Sanger as resolver/reference. F: Only 151 quality-selected sequencing results from 237 valid qPCR specimens inform paired comparisons. A: Restricted applicability to unselected, first-line suspected onychomycosis: Verification-selected subgroup. 242 PAS-submitted toenails; 237 valid qPCR; 151 high/moderate-quality Sanger sequences	Workbook v0.12 Evidence_Table row 8; Full_Text_Screening record OMD-2023-Marin-Maldonado; source locator: Full text Tables 3-6. Existing full-text extraction only; source PDF not re-examined for this appraisal. Overlap: None identified
OMD-2026-Dey [32]	Unclear / Unclear / Unclear / Unclear / Low	S: Recruitment sequence, consecutive enrollment and the complete exclusion process are not established by the available extraction. 139 clinically suspected onychomycosis outpatients, July 2023-June 2024. I: Prospective threshold selection and blinded interpretation are not established by the available assessment record. Species calls inferred from ITS amplicon size are not equivalent to sequence-confirmed identity; this concern must not be confused with binary fungal amplification.	C: The extracted endpoint or its handling has unresolved reporting/target-scope qualifications. Culture used by authors as gold standard. F: Completeness of valid, same-specimen paired observations is not fully established for all reported analyses. Species identification from universal ITS amplicon size without sequencing/RFLP raises classification-bias concerns. A: Clinical nail comparison applicable within its stated organism target and setting; it does not establish all-pathogen accuracy or clinical benefit. Bangladesh; Sir Salimullah Medical College, Dhaka	Workbook v0.12 Evidence_Table row 9; Full_Text_Screening record OMD-2026-Dey; source locator: Full text Table 5. Existing full-text extraction only; source PDF not re-examined for this appraisal. Overlap: None identified
OMD-2026-Villanueva-Reyes [46]	High / Unclear / Low / Low / High	S: Conventional-test-defined cases and healthy controls create a two-gate, selected spectrum; clinical-case culture rows have a different denominator. I: Prospective threshold selection and blinded interpretation are not established by the available assessment record. Molecular endpoint: Broad fungal sequencing.	C: For raw paired discordance, the comparator is interpreted as a separate detection method rather than disease truth. Case/control classification based on KOH and/or culture. F: The extracted clinical/nail endpoint has an exact paired denominator; no additional selective verification or unresolved invalid-result loss is documented for those raw cells. Same nail evaluated by KOH, culture, ITS PCR and nanopore sequencing. A: Restricted applicability to unselected, first-line suspected onychomycosis: Case/control spectrum. 51 composite-confirmed onychomycosis cases and 53 healthy nails; molecular results in 103	Workbook v0.12 Evidence_Table row 10; Full_Text_Screening record OMD-2026-Villanueva-Reyes; source locator: Full text Results and diagnostic-performance table. Existing full-text extraction only; source PDF not re-examined for this appraisal. Overlap: None identified
OMD-2006-Yoshimura [51]	High / Unclear / Low / Unclear / High	S: All enrolled nails were KOH-confirmed, excluding clinically suspected microscopy-negative disease. I: Prospective threshold selection and blinded interpretation are not established by the available assessment record. Molecular endpoint: Dermatophyte identification.	C: For raw paired discordance, the comparator is interpreted as a separate detection method rather than disease truth. KOH-confirmed clinical cohort; culture compared descriptively. F: Completeness of valid, same-specimen paired observations is not fully established for all reported analyses. Margins do not establish paired cells; culture isolates were themselves molecularly confirmed. A: Restricted applicability to unselected, first-line suspected onychomycosis: Complete paired-cross-tab not reported. 100 KOH-confirmed tinea unguium nail specimens	Workbook v0.12 Evidence_Table row 11; Full_Text_Screening record OMD-2006-Yoshimura; source locator: Full text Results and species table. Existing full-text extraction only; source PDF not re-examined for this appraisal. Overlap: None identified
OMD-2018-Bao [52]	Unclear / Unclear / Low / Unclear / Low	S: Recruitment sequence, consecutive enrollment and the complete exclusion process are not established by the available extraction. 204 specimens from 187 patients with suspected onychomycosis. I: Prospective threshold selection and blinded interpretation are not established by the available assessment record. Molecular endpoint: Broad fungal species identification.	C: For raw paired discordance, the comparator is interpreted as a separate detection method rather than disease truth. Conventional methods; author composite KOH-or-culture also reported. F: Completeness of valid, same-specimen paired observations is not fully established for all reported analyses. Authors' composite totals are internally inconsistent by one sample; binary culture comparison is exact. A: Clinical nail comparison applicable within its stated organism target and setting; it does not establish all-pathogen accuracy or clinical benefit. China; clinical dermatology/mycology	Workbook v0.12 Evidence_Table row 12; Full_Text_Screening record OMD-2018-Bao; source locator: Full text diagnostic comparison table. Existing full-text extraction only; source PDF not re-examined for this appraisal. Overlap: None identified
OMD-2019-Gustafson [18]	Unclear / Unclear / Low / Low / Low	S: Recruitment sequence, consecutive enrollment and the complete exclusion process are not established by the available extraction. 425-sample PCR-culture cohort; separate sequential 2,452-sample PCR-culture-histology cohort. I: Prospective threshold selection and blinded interpretation are not established by the available assessment record. Molecular endpoint: Up to 15 fungi across dermatophyte, saprophyte/NDM and yeast classes.	C: For raw paired discordance, the comparator is interpreted as a separate detection method rather than disease truth. Histopathology and discordant sequencing used for adjudication; culture not assumed infallible. F: The extracted clinical/nail endpoint has an exact paired denominator; no additional selective verification or unresolved invalid-result loss is documented for those raw cells. Dry nail divided for histopathology, molecular testing and culture. A: Clinical nail comparison applicable within its stated organism target and setting; it does not establish all-pathogen accuracy or clinical benefit. United States; Bako Diagnostics	Workbook v0.12 Evidence_Table row 13; Full_Text_Screening record OMD-2019-Gustafson; source locator: Full text Tables 2, 5A and 6. Existing full-text extraction only; source PDF not re-examined for this appraisal. Overlap: Bako assay family; no demonstrated temporal overlap with later national cohort
OMD-2007-Garg [53]	Unclear / Unclear / Low / Low / Unclear	S: Recruitment sequence, consecutive enrollment and the complete exclusion process are not established by the available extraction. 152 clinically suspected onychomycosis patients; 83 empirically treated nonresponders and 69 untreated. I: Prospective threshold selection and blinded interpretation are not established by the available assessment record. Molecular endpoint: Dermatophyte-specific.	C: For raw paired discordance, the comparator is interpreted as a separate detection method rather than disease truth. Conventional methods; no composite adjudication. F: The extracted clinical/nail endpoint has an exact paired denominator; no additional selective verification or unresolved invalid-result loss is documented for those raw cells. Nail portions for KOH, culture, first-round PCR and nested PCR. A: Applicability depends on treatment, sampling, target or subgroup qualifications: Strong treatment confounding; zero cells; culture comparator restricted to dermatophytes. Treatment exposure: 83 received 2-3 months empirical antifungal therapy; analyze separately	Workbook v0.12 Evidence_Table row 14; Full_Text_Screening record OMD-2007-Garg; source locator: Full text Table 1 and Results. Existing full-text extraction only; source PDF not re-examined for this appraisal. Overlap: None identified
OMD-2007-Brillowska-Dabrowska [23]	Unclear / Unclear / Low / Low / Low	S: Recruitment sequence, consecutive enrollment and the complete exclusion process are not established by the available extraction. 118 clinically diseased nails submitted for routine microscopy and culture. I: Prospective threshold selection and blinded interpretation are not established by the available assessment record. Molecular endpoint: Dermatophytes plus specific *T. rubrum* call.	C: For raw paired discordance, the comparator is interpreted as a separate detection method rather than disease truth. Conventional methods; detailed discordance review. F: The extracted clinical/nail endpoint has an exact paired denominator; no additional selective verification or unresolved invalid-result loss is documented for those raw cells. Routine nail material; direct DNA extraction after conventional sampling. A: Clinical nail comparison applicable within its stated organism target and setting; it does not establish all-pathogen accuracy or clinical benefit. Denmark; Statens Serum Institut	Workbook v0.12 Evidence_Table row 15; Full_Text_Screening record OMD-2007-Brillowska-Dabrowska; source locator: Full text Results; Table 2 narrative. Existing full-text extraction only; source PDF not re-examined for this appraisal. Overlap: None identified
OMD-2006-Kardjeva [21]	Unclear / Unclear / Low / High / Low	S: Recruitment sequence, consecutive enrollment and the complete exclusion process are not established by the available extraction. 195 direct nail specimens; separate 66 previously collected etiologic strains. I: Prospective threshold selection and blinded interpretation are not established by the available assessment record. PCR inhibition was documented in seven negative nails; negative-result interpretation requires the original handling rules.	C: For raw paired discordance, the comparator is interpreted as a separate detection method rather than disease truth. Molecular workflow with sequencing/RFLP resolver. F: Seven PCR-negative nails were reported as inhibited in the existing extraction; final resolution/retest handling is not established. Keep source-reported rows flagged and exclude from strict valid-result sensitivity. A: Clinical nail comparison applicable within its stated organism target and setting; it does not establish all-pathogen accuracy or clinical benefit. Bulgaria/Greece with German molecular laboratory	Workbook v0.12 Evidence_Table row 16; Full_Text_Screening record OMD-2006-Kardjeva; source locator: Full text Table 2. Existing full-text extraction only; source PDF not re-examined for this appraisal. Overlap: None identified
OMD-2026-Guegan [16]	Unclear / Unclear / Low / Low / Low	S: Recruitment sequence, consecutive enrollment and the complete exclusion process are not established by the available extraction. 487 valid samples from 407 patients; nail subgroup n=316. I: Prospective threshold selection and blinded interpretation are not established by the available assessment record. Molecular endpoint: Dermatophyte panel plus *C. albicans*; automated chip interpretation.	C: For raw paired discordance, the comparator is interpreted as a separate detection method rather than disease truth. Culture and composite conventional standards; DermaGenius resolver plus clinical/sample evidence for discordances. F: The extracted clinical/nail endpoint has an exact paired denominator; no additional selective verification or unresolved invalid-result loss is documented for those raw cells. Nail, skin and scalp clinical samples; nail-specific analysis reported. A: Clinical nail comparison applicable within its stated organism target and setting; it does not establish all-pathogen accuracy or clinical benefit. France; prospective multicenter laboratories	Workbook v0.12 Evidence_Table row 17; Full_Text_Screening record OMD-2026-Guegan; source locator: Full text Tables 1-3. Existing full-text extraction only; source PDF not re-examined for this appraisal. Overlap: None identified
OMD-2022-Pospischil [44]	High / Unclear / Low / High / High	S: Molecular testing was triggered by inconclusive or discordant conventional findings, rather than applied to the original consecutive population. I: Prospective threshold selection and blinded interpretation are not established by the available assessment record. Molecular endpoint: Broad fungal identification.	C: For raw paired discordance, the comparator is interpreted as a separate detection method rather than disease truth. No fixed gold standard; conditional molecular add-on. F: Conventional-test results determine whether molecular verification is performed; the 1,148 molecular specimens are not the full 16,094-nail series. A: Restricted applicability to unselected, first-line suspected onychomycosis: Reflex/conditional. 16,094 consecutive nail samples; PCR/sequencing applied to 1,148 inconclusive/inconsistent cases	Workbook v0.12 Evidence_Table row 18; Full_Text_Screening record OMD-2022-Pospischil; source locator: Full text Tables 1-3. Existing full-text extraction only; source PDF not re-examined for this appraisal. Overlap: Reinhardt2021 thesis is an overlapping report already linked to Pospischil2022; not an independent study. Possible relation to selected Lausanne reports requires caution.
OMD-2012-Verrier [54]	High / Unclear / Low / Low / High	S: Primary comparison restricted to microscopy-positive abnormal nails; the initial unselected denominator was larger. I: Prospective threshold selection and blinded interpretation are not established by the available assessment record. Molecular endpoint: Dermatophytes plus 12 NDF markers, expandable reference ladder.	C: For raw paired discordance, the comparator is interpreted as a separate detection method rather than disease truth. Microscopy-positive abnormal nail; sequencing for unresolved peaks. F: The extracted clinical/nail endpoint has an exact paired denominator; no additional selective verification or unresolved invalid-result loss is documented for those raw cells. One nail split for fluorescence microscopy, culture, and stored direct molecular testing. A: Restricted applicability to unselected, first-line suspected onychomycosis: Microscopy-positive direct nail cohort. 679 abnormal nails; primary comparison in 624 microscopy-positive specimens; 20 healthy controls	Workbook v0.12 Evidence_Table row 19; Full_Text_Screening record OMD-2012-Verrier; source locator: Full text Table 2 and Results. Existing full-text extraction only; source PDF not re-examined for this appraisal. Overlap: Potential relation to earlier Lausanne PCR-RFLP cohorts; no duplicate confirmed
OMD-2022-Bieber [29]	Unclear / Unclear / High / Low / Low	S: Recruitment sequence, consecutive enrollment and the complete exclusion process are not established by the available extraction. 67 suspected OM cases plus 24 healthy nail controls; separate swab substudy. I: Prospective threshold selection and blinded interpretation are not established by the available assessment record. Molecular endpoint: 56 fungal pathogens.	C: High for the index-containing/composite or selectively adjudicated results; raw culture and microscopy pair counts do not inherit this incorporation bias. F: The extracted clinical/nail endpoint has an exact paired denominator; no additional selective verification or unresolved invalid-result loss is documented for those raw cells. Scraped nail material for microscopy, culture, DNA chip; PCR-only discordances sequenced. A: Clinical nail comparison applicable within its stated organism target and setting; it does not establish all-pathogen accuracy or clinical benefit. Germany; single-center prospective study	Workbook v0.12 Evidence_Table row 20; Full_Text_Screening record OMD-2022-Bieber; source locator: Full text Figure 1 and Table 1. Existing full-text extraction only; source PDF not re-examined for this appraisal. Overlap: None identified
OMD-2024-Eijkenboom [55]	Unclear / Unclear / High / Unclear / High	S: Recruitment sequence, consecutive enrollment and the complete exclusion process are not established by the available extraction. 86 clinically suspected cases and 14 controls; analyzed composite-positive/negative groups. I: Prospective threshold selection and blinded interpretation are not established by the available assessment record. Molecular endpoint: Commercial fungal panel.	C: The disease composite includes PCR, so PCR performance against that composite is circular; no paired molecular/conventional cells are extracted. F: Completeness of valid, same-specimen paired observations is not fully established for all reported analyses. Index-test incorporation/circularity and no molecular cross-tab; primary research question is non-molecular imaging. A: Restricted applicability to unselected, first-line suspected onychomycosis: Narrative/supportive. 86 clinically suspected cases and 14 controls; analyzed composite-positive/negative groups	Workbook v0.12 Evidence_Table row 21; Full_Text_Screening record OMD-2024-Eijkenboom; source locator: Full text Table 2 and Methods. Existing full-text extraction only; source PDF not re-examined for this appraisal. Overlap: None identified
OMD-2024-Katsiaunis [34]	Unclear / Unclear / Low / Low / Low	S: Recruitment sequence, consecutive enrollment and the complete exclusion process are not established by the available extraction. 306 patients with both PCR and fungal culture. I: Prospective threshold selection and blinded interpretation are not established by the available assessment record. Molecular endpoint: Separate dermatophyte and NDM categories.	C: For raw paired discordance, the comparator is interpreted as a separate detection method rather than disease truth. Culture comparison with histology resolver and clinical outcomes. F: The extracted clinical/nail endpoint has an exact paired denominator; no additional selective verification or unresolved invalid-result loss is documented for those raw cells. Same patient nail testing with PCR and culture; histology used to adjudicate discordances. A: Clinical nail comparison applicable within its stated organism target and setting; it does not establish all-pathogen accuracy or clinical benefit. United States; academic center in New York City with external laboratory testing	Workbook v0.12 Evidence_Table row 22; Full_Text_Screening record OMD-2024-Katsiaunis; source locator: Full text Table I and Results. Existing full-text extraction only; source PDF not re-examined for this appraisal. Overlap: Potential Bako assay linkage; patient/time overlap with national 2022-23 cohort unlikely but must be checked
OMD-2024-Harel [30]	Unclear / Unclear / High / Low / Low	S: Recruitment sequence, consecutive enrollment and the complete exclusion process are not established by the available extraction. 312 dermatophytosis specimens from 236 patients; nail subgroup 182 (161 toenails, 21 fingernails). I: Prospective threshold selection and blinded interpretation are not established by the available assessment record. Molecular endpoint: *T. rubrum*, *T. mentagrophytes*/*interdigitale*/*benhamiae* complex, *T. tonsurans* and Microsporum; no NDM/yeast coverage.	C: High for final-diagnosis composite rows that incorporate molecular/contextual findings; raw nail culture cells are interpreted separately. F: The extracted clinical/nail endpoint has an exact paired denominator; no additional selective verification or unresolved invalid-result loss is documented for those raw cells. Routine specimens tested by direct examination, culture and Novaplex qPCR. A: Clinical nail comparison applicable within its stated organism target and setting; it does not establish all-pathogen accuracy or clinical benefit. Israel; tertiary dermatology/mycology laboratory	Workbook v0.12 Evidence_Table row 23; Full_Text_Screening record OMD-2024-Harel; source locator: Full text Tables 4-5; Results. Existing full-text extraction only; source PDF not re-examined for this appraisal. Overlap: None identified
OMD-2007-Savin [56]	Unclear / Unclear / Low / High / Unclear	S: Recruitment sequence, consecutive enrollment and the complete exclusion process are not established by the available extraction. 546 tested specimens: 236 regional proximal samples, 75 linked distal samples, 127 routine regional samples and 108 healthy controls. I: Prospective threshold selection and blinded interpretation are not established by the available assessment record. Positive-only and gray-zone-positive thresholds yield different results and are kept as separate, dependent variants.	C: For raw paired discordance, the comparator is interpreted as a separate detection method rather than disease truth. Comparator-specific analyses; no single gold standard. F: The distal-versus-proximal rows compare linked but anatomically different samples; these are not interchangeable with same-specimen proximal rows. Threshold alternatives also reuse samples. A: Applicability depends on treatment, sampling, target or subgroup qualifications: Distal and proximal samples are anatomically linked rather than identical aliquots; gray-zone handling changes cells; one reported routine-sample composite is internally incomplete.	Workbook v0.12 Evidence_Table row 24; Full_Text_Screening record OMD-2007-Savin; source locator: Full text Tables 2-5. Existing full-text extraction only; source PDF not re-examined for this appraisal. Overlap: None identified
OMD-2024-Navarro-Perez [31]	Unclear / Unclear / Low / Low / Low	S: Recruitment sequence, consecutive enrollment and the complete exclusion process are not established by the available extraction. 125 patients with clinically suspected toenail onychomycosis. I: Prospective threshold selection and blinded interpretation are not established by the available assessment record. Molecular endpoint: Fungal/dermatophyte molecular diagnosis as reported.	C: For raw paired discordance, the comparator is interpreted as a separate detection method rather than disease truth. PCR-or-culture laboratory positivity used in comparative analysis. F: The extracted clinical/nail endpoint has an exact paired denominator; no additional selective verification or unresolved invalid-result loss is documented for those raw cells. Same affected toenail assessed by dermatoscopy, culture and PCR. A: Clinical nail comparison applicable within its stated organism target and setting; it does not establish all-pathogen accuracy or clinical benefit. Spain; dermatology clinic	Workbook v0.12 Evidence_Table row 25; Full_Text_Screening record OMD-2024-Navarro-Perez; source locator: Full text Results and diagnostic table. Existing full-text extraction only; source PDF not re-examined for this appraisal. Overlap: None identified
OMD-2024-Evrard [45]	Unclear / Unclear / High / Low / Low	S: Recruitment sequence, consecutive enrollment and the complete exclusion process are not established by the available extraction. 106 nail samples from 102 patients with suspected onychomycosis. I: Prospective threshold selection and blinded interpretation are not established by the available assessment record. Molecular endpoint: Dermatophytes and selected fungi in a commercial multiplex panel.	C: High for PCR-containing composite and algorithm-versus-composite claims; the raw systematic PCR/culture table is separate. F: The extracted clinical/nail endpoint has an exact paired denominator; no additional selective verification or unresolved invalid-result loss is documented for those raw cells. Nail material tested systematically by DermaGenius PCR and culture; calcofluor microscopy informed selective culture algorithm. A: Clinical nail comparison applicable within its stated organism target and setting; it does not establish all-pathogen accuracy or clinical benefit. Belgium; university hospital	Workbook v0.12 Evidence_Table row 26; Full_Text_Screening record OMD-2024-Evrard; source locator: Full text Tables 2-4; Results. Existing full-text extraction only; source PDF not re-examined for this appraisal. Overlap: None identified
OMD-2017-Iwanaga [43]	High / Unclear / Low / Low / High	S: Treatment-monitoring cohort required KOH-confirmed disease and subsequently provided repeated samples. I: Prospective threshold selection and blinded interpretation are not established by the available assessment record. Molecular endpoint: Dermatophyte DNA and viability-related quantitative signal.	C: For raw paired discordance, the comparator is interpreted as a separate detection method rather than disease truth. Serial conventional results; no independent gold standard. F: The extracted clinical/nail endpoint has an exact paired denominator; no additional selective verification or unresolved invalid-result loss is documented for those raw cells. Serial nail lesion samples; culture, KOH and dermatophyte actin qPCR. A: Restricted applicability to unselected, first-line suspected onychomycosis: Treatment monitoring. 14 KOH-confirmed big-toenail tinea unguium patients treated with oral terbinafine	Workbook v0.12 Evidence_Table row 27; Full_Text_Screening record OMD-2017-Iwanaga; source locator: Full text Tables 1-2 and Results. Existing full-text extraction only; source PDF not re-examined for this appraisal. Overlap: Repeated measures within the same patients
OMD-2018-Lubis [57]	Unclear / Unclear / Low / Low / Low	S: Recruitment sequence, consecutive enrollment and the complete exclusion process are not established by the available extraction. 35 patients with clinically suspected onychomycosis. I: Prospective threshold selection and blinded interpretation are not established by the available assessment record. Molecular endpoint: Dermatophyte/fungal identification.	C: For raw paired discordance, the comparator is interpreted as a separate detection method rather than disease truth. Culture used as comparator. F: The extracted clinical/nail endpoint has an exact paired denominator; no additional selective verification or unresolved invalid-result loss is documented for those raw cells. Same nail specimen tested by culture and PCR-RFLP. A: Clinical nail comparison applicable within its stated organism target and setting; it does not establish all-pathogen accuracy or clinical benefit. Indonesia; hospital dermatology clinic	Workbook v0.12 Evidence_Table row 28; Full_Text_Screening record OMD-2018-Lubis; source locator: Full text Table 2. Existing full-text extraction only; source PDF not re-examined for this appraisal. Overlap: None identified
OMD-2014-Han [58]	Unclear / Unclear / High / Low / Low	S: Recruitment sequence, consecutive enrollment and the complete exclusion process are not established by the available extraction. 32 nail specimens from 29 patients with suspected onychomycosis; 10 healthy nails separately. I: Prospective threshold selection and blinded interpretation are not established by the available assessment record. Molecular endpoint: Dermatophytes and Candida albicans.	C: High for the selectively adjudicated composite, which uses selected sequencing, treatment response and clinical information; the raw culture comparison remains separate. F: The extracted clinical/nail endpoint has an exact paired denominator; no additional selective verification or unresolved invalid-result loss is documented for those raw cells. Same nail specimen evaluated by culture and PCR oligonucleotide array. A: Clinical nail comparison applicable within its stated organism target and setting; it does not establish all-pathogen accuracy or clinical benefit. Taiwan; medical center	Workbook v0.12 Evidence_Table row 29; Full_Text_Screening record OMD-2014-Han; source locator: Full text Results and Tables 2-3. Existing full-text extraction only; source PDF not re-examined for this appraisal. Overlap: None identified
OMD-2026-Wu [47]	Unclear / Unclear / Low / Low / Low	S: Recruitment sequence, consecutive enrollment and the complete exclusion process are not established by the available extraction. 81 clinical nail specimens with suspected onychomycosis. I: Prospective threshold selection and blinded interpretation are not established by the available assessment record. Molecular endpoint: Panfungal plus *T. rubrum*, *T. interdigitale*, pan-Candida/*C. albicans*, pan-Trichosporon and pan-Aspergillus.	C: For raw paired discordance, the comparator is interpreted as a separate detection method rather than disease truth. No gold standard; random-effects latent-class model. F: The extracted clinical/nail endpoint has an exact paired denominator; no additional selective verification or unresolved invalid-result loss is documented for those raw cells. Same nail specimen tested by microscopy, culture and multi-tube RPA. A: Clinical nail comparison applicable within its stated organism target and setting; it does not establish all-pathogen accuracy or clinical benefit. China; hospital dermatology/mycology	Workbook v0.12 Evidence_Table row 30; Full_Text_Screening record OMD-2026-Wu; source locator: Full text Table 3 and latent-class results. Existing full-text extraction only; source PDF not re-examined for this appraisal. Overlap: None identified
OMD-2021-Kabtani [59]	Unclear / Unclear / Unclear / Unclear / Unclear	S: Recruitment sequence, consecutive enrollment and the complete exclusion process are not established by the available extraction. 105 dermatophytosis specimens; nail subgroup n=19. I: Prospective threshold selection and blinded interpretation are not established by the available assessment record. Molecular endpoint: Dermatophyte detection.	C: The extracted endpoint or its handling has unresolved reporting/target-scope qualifications. Direct examination/culture conventional classification. F: Completeness of valid, same-specimen paired observations is not fully established for all reported analyses. Very small nail subgroup; Table 7 conflicts with Table 6/text for commercial-kit positives; mixed-site parent cohort. A: Applicability depends on treatment, sampling, target or subgroup qualifications: Very small nail subgroup; Table 7 conflicts with Table 6/text for commercial-kit positives; mixed-site parent cohort.	Workbook v0.12 Evidence_Table row 31; Full_Text_Screening record OMD-2021-Kabtani; source locator: Full text Tables 5-7; Results. Existing full-text extraction only; source PDF not re-examined for this appraisal. Overlap: None identified
OMD-2010-Sato [22]	High / Unclear / Low / Low / High	S: All clinical specimens were microscopy-positive; the intended-use negative spectrum is absent. I: Prospective threshold selection and blinded interpretation are not established by the available assessment record. Broad fungal signal and species identification are separate molecular endpoints.	C: For raw paired discordance, the comparator is interpreted as a separate detection method rather than disease truth. Microscopy-positive entry spectrum; culture comparison. F: The extracted clinical/nail endpoint has an exact paired denominator; no additional selective verification or unresolved invalid-result loss is documented for those raw cells. Microscopy-positive nail material divided for culture and PCR-DNA microarray. A: Restricted applicability to unselected, first-line suspected onychomycosis: Microscopy-positive spectrum. 106 microscopy-confirmed onychomycosis nail specimens; patient count internally reported as 83 versus 86	Workbook v0.12 Evidence_Table row 32; Full_Text_Screening record OMD-2010-Sato; source locator: Full text Tables 2-3 and Methods. Existing full-text extraction only; source PDF not re-examined for this appraisal. Overlap: None identified
OMD-2023-Gupta-NDM [20]	Unclear / Unclear / Low / Low / Low	S: Recruitment sequence, consecutive enrollment and the complete exclusion process are not established by the available extraction. 512 toenail specimens/patients evaluated for onychomycosis and NDM. I: Prospective threshold selection and blinded interpretation are not established by the available assessment record. Molecular endpoint: Dermatophytes, yeasts and seven NDM genera; separate bacterial Pseudomonas output.	C: For raw paired discordance, the comparator is interpreted as a separate detection method rather than disease truth. Histopathology for fungal presence; culture for NDM comparison. F: The extracted clinical/nail endpoint has an exact paired denominator; no additional selective verification or unresolved invalid-result loss is documented for those raw cells. Single-point nail sample tested by multiplex PCR, histopathology and culture. A: Clinical nail comparison applicable within its stated organism target and setting; it does not establish all-pathogen accuracy or clinical benefit. United States; national podiatric pathology laboratory	Workbook v0.12 Evidence_Table row 33; Full_Text_Screening record OMD-2023-Gupta-NDM; source locator: Full text Tables 2-3. Existing full-text extraction only; source PDF not re-examined for this appraisal. Overlap: Potential overlap with other Bako laboratory cohorts requires date/population check
OMD-2025-Santosh [60]	Unclear / Unclear / Unclear / Unclear / High	S: Recruitment sequence, consecutive enrollment and the complete exclusion process are not established by the available extraction. Nine bags of communal ground toenail dust representing approximately 400-500 podiatric patients. I: Prospective threshold selection and blinded interpretation are not established by the available assessment record. Molecular endpoint: *T. rubrum* and *T. interdigitale*.	C: The extracted endpoint or its handling has unresolved reporting/target-scope qualifications. No patient-level reference standard. F: Completeness of valid, same-specimen paired observations is not fully established for all reported analyses. Ecological pooled unit, historical comparator and no individual clinical phenotype. A: Restricted applicability to unselected, first-line suspected onychomycosis: Narrative only-no individual diagnostic unit. Nine bags of communal ground toenail dust representing approximately 400-500 podiatric patients	Workbook v0.12 Evidence_Table row 34; Full_Text_Screening record OMD-2025-Santosh; source locator: Full text Results; publisher DOI 10.3390/jof11070520. Existing full-text extraction only; source PDF not re-examined for this appraisal. Overlap: Same pooled material as authors' earlier culture report
OMD-2020-Pakshir [61]	Unclear / Unclear / Low / Low / Low	S: Recruitment sequence, consecutive enrollment and the complete exclusion process are not established by the available extraction. 137 suspected onychomycosis nail specimens plus 10 healthy controls. I: Prospective threshold selection and blinded interpretation are not established by the available assessment record. Molecular endpoint: Candida species except reduced coverage for *C. glabrata*.	C: For raw paired discordance, the comparator is interpreted as a separate detection method rather than disease truth. Culture comparison; microscopy supportive. F: The extracted clinical/nail endpoint has an exact paired denominator; no additional selective verification or unresolved invalid-result loss is documented for those raw cells. Same nail specimen tested by microscopy, culture and TEF1-alpha PCR. A: Clinical nail comparison applicable within its stated organism target and setting; it does not establish all-pathogen accuracy or clinical benefit. Iran; Shiraz medical centers	Workbook v0.12 Evidence_Table row 35; Full_Text_Screening record OMD-2020-Pakshir; source locator: Full text Table 2. Existing full-text extraction only; source PDF not re-examined for this appraisal. Overlap: None identified
OMD-2022-Trovato [41]	Unclear / Unclear / Low / Low / Unclear	S: Recruitment sequence, consecutive enrollment and the complete exclusion process are not established by the available extraction. 109 toenail specimens: 52 untreated and 57 after empirical antifungal therapy. I: Prospective threshold selection and blinded interpretation are not established by the available assessment record. Molecular endpoint: Dermatophyte detection.	C: For raw paired discordance, the comparator is interpreted as a separate detection method rather than disease truth. Conventional microscopy-and/or-culture classification. F: The extracted clinical/nail endpoint has an exact paired denominator; no additional selective verification or unresolved invalid-result loss is documented for those raw cells. Same toenail specimen tested by microscopy, culture and multiplex real-time PCR. A: Applicability depends on treatment, sampling, target or subgroup qualifications: Treatment confounding; conventional composite has target mismatch; zero cells in composite comparisons. Treatment exposure: 57 previously treated empirically; 52 untreated	Workbook v0.12 Evidence_Table row 36; Full_Text_Screening record OMD-2022-Trovato; source locator: Full text Table 2 and Results. Existing full-text extraction only; source PDF not re-examined for this appraisal. Overlap: None identified
OMD-2017-Vrioni [62]	Unclear / Unclear / Low / Unclear / Unclear	S: Recruitment sequence, consecutive enrollment and the complete exclusion process are not established by the available extraction. 252 separate patients with suspected onychomycosis. I: Prospective threshold selection and blinded interpretation are not established by the available assessment record. Molecular endpoint: Pan-dermatophyte CHS1 plus *T. rubrum* ITS2.	C: For raw paired discordance, the comparator is interpreted as a separate detection method rather than disease truth. Microscopy and/or dermatophyte culture; NDM culture classified dermatophyte-negative. F: Completeness of valid, same-specimen paired observations is not fully established for all reported analyses. Previous treatment permitted; author composite target mismatch; figure and narrative contain count inconsistencies for microscopy/NDM strata. A: Applicability depends on treatment, sampling, target or subgroup qualifications: Previous treatment permitted; author composite target mismatch; figure and narrative contain count inconsistencies for microscopy/NDM strata. Treatment exposure: Prior antifungal treatment was not excluded; 12 PCR-positive specimens were from previously treated patients	Workbook v0.12 Evidence_Table row 37; Full_Text_Screening record OMD-2017-Vrioni; source locator: Full text Results and Figure 2. Targeted revision PDF recheck: clinical nail cohort and sampling allocation. Overlap: None identified
OMD-2026-Gellrich [35]	Low / Unclear / Unclear / Unclear / Unclear	S: The source explicitly reports consecutive samples requested for both tests; inference remains restricted to that submitted, dual-tested population. I: Prospective threshold selection and blinded interpretation are not established by the available assessment record. Manufacturer/software fluorescence cut-offs are reported; reader blinding is not established. Mold growth was suppressed by the culture medium, limiting broad fungal comparisons.	C: The extracted endpoint or its handling has unresolved reporting/target-scope qualifications. No independent reference standard; limited clinical follow-up of discordances. F: Completeness of valid, same-specimen paired observations is not fully established for all reported analyses. One table reports 51 nails while abstract/text/Table 2 report 52; mixed-site follow-up; mold-inhibiting agar; microscopy omitted. A: Applicability depends on treatment, sampling, target or subgroup qualifications: One table reports 51 nails while abstract/text/Table 2 report 52; mixed-site follow-up; mold-inhibiting agar; microscopy omitted.	Workbook v0.12 Evidence_Table row 38; Full_Text_Screening record OMD-2026-Gellrich; source locator: Full text Table 2 and overall overlap results. Targeted revision PDF recheck: consecutive dual-test submissions, exclusions, cut-offs and culture medium. Overlap: None identified
OMD-2025-Steixner [63]	Unclear / Unclear / Low / High / High	S: Recruitment sequence, consecutive enrollment and the complete exclusion process are not established by the available extraction. 4483 skin, hair and nail specimens submitted 2018-2024. I: Prospective threshold selection and blinded interpretation are not established by the available assessment record. Molecular endpoint: CHS1 pan-dermatophyte detection with multilocus sequencing.	C: For raw paired discordance, the comparator is interpreted as a separate detection method rather than disease truth. Stage-wise algorithm; conventional tests not applied to PCR-positive specimens. F: Conventional tests are conditional on PCR negativity/inconclusiveness, and culture is conditional on positive microscopy. A: Restricted applicability to unselected, first-line suspected onychomycosis: Reflex/conditional mixed-site workflow. 4483 skin, hair and nail specimens submitted 2018-2024	Workbook v0.12 Evidence_Table row 39; Full_Text_Screening record OMD-2025-Steixner; source locator: Full text Figures 2-4 and Results. Targeted revision PDF recheck: PCR-first conditional testing workflow. Overlap: None identified
OMD-2026-Lotsch [36]	Unclear / Unclear / Unclear / High / High	S: Recruitment sequence, consecutive enrollment and the complete exclusion process are not established by the available extraction. 124 clinical samples with suspected dermatophytosis. I: Prospective threshold selection and blinded interpretation are not established by the available assessment record. The authors discuss possible reagent-related *F. solani* detections; analytical signal and clinically causal infection should not be equated.	C: The extracted endpoint or its handling has unresolved reporting/target-scope qualifications. No independent resolver; reagent and reference-strain controls. F: Six cultures were missing and no nail-specific cross-table is available; inhibited and negative molecular states are combined in aggregate reporting. A: Restricted applicability to unselected, first-line suspected onychomycosis: Mixed-site assay comparison. 124 clinical samples with suspected dermatophytosis	Workbook v0.12 Evidence_Table row 40; Full_Text_Screening record OMD-2026-Lotsch; source locator: Full text Results and Table 3. Targeted revision PDF recheck: missing cultures, inhibitor state and contamination discussion. Overlap: None identified
OMD-2025-Muranen [64]	High / Unclear / Unclear / High / High	S: Poster cohort was selected to be microscopy-positive and dermatophyte-culture-negative/inconclusive. I: Prospective threshold selection and blinded interpretation are not established by the available assessment record. Molecular endpoint: Broad fungal sequencing and commercial microarray.	C: The extracted endpoint or its handling has unresolved reporting/target-scope qualifications. No independent reference standard; culture status defined the cohort. F: Culture status defines entry and there is no unselected conventional-positive stratum; possible overlap with the Lausanne source population is unresolved. A: Restricted applicability to unselected, first-line suspected onychomycosis: Verification-selected grey literature. 817 microscopy-positive nails with culture negative for dermatophytes or inconclusive	Workbook v0.12 Evidence_Table row 41; Full_Text_Screening record OMD-2025-Muranen; source locator: Conference poster result diagram. Targeted revision PDF recheck: verification-selected cohort and grey-literature limitations. Overlap: Likely related/overlapping Lausanne-Pospischil source population; extent and study period not reported
OMD-2023-Guschin [65]	Unclear / Unclear / Low / Low / Low	S: Recruitment sequence, consecutive enrollment and the complete exclusion process are not established by the available extraction. 258 patients: 154 skin, 62 hair and 42 nail specimens. I: Prospective threshold selection and blinded interpretation are not established by the available assessment record. Molecular endpoint: *T. rubrum*, *T. mentagrophytes* complex, *T. tonsurans*, *M. canis* and *E. floccosum*.	C: For raw paired discordance, the comparator is interpreted as a separate detection method rather than disease truth. Selective Sanger sequencing of PCR+/conventional− results. F: The extracted clinical/nail endpoint has an exact paired denominator; no additional selective verification or unresolved invalid-result loss is documented for those raw cells. Separate routine samples collected simultaneously for KOH microscopy, culture and RT-PCR. A: Clinical nail comparison applicable within its stated organism target and setting; it does not establish all-pathogen accuracy or clinical benefit. Russia; Moscow dermatovenereology center	Workbook v0.12 Evidence_Table row 42; Full_Text_Screening record OMD-2023-Guschin; source locator: Full text Table 2 and Results. Targeted revision PDF recheck: simultaneous specimen testing and selective sequencing. Overlap: None identified
OMD-2022-Makled [66]	Unclear / Unclear / Low / Low / Low	S: Recruitment sequence, consecutive enrollment and the complete exclusion process are not established by the available extraction. 100 clinically diagnosed onychomycosis patients. I: Prospective threshold selection and blinded interpretation are not established by the available assessment record. Only 30 products were sequenced; binary amplification and sequence-confirmed species identification are different outcomes.	C: For raw paired discordance, the comparator is interpreted as a separate detection method rather than disease truth. Culture used by authors as cornerstone. F: The extracted clinical/nail endpoint has an exact paired denominator; no additional selective verification or unresolved invalid-result loss is documented for those raw cells. Each nail specimen divided into five portions for KOH, PAS, fluorescence, culture and PCR. A: Clinical nail comparison applicable within its stated organism target and setting; it does not establish all-pathogen accuracy or clinical benefit. Egypt; Menoufia University dermatology/microbiology	Workbook v0.12 Evidence_Table row 43; Full_Text_Screening record OMD-2022-Makled; source locator: Full text Table 3 and Methods. Targeted revision PDF recheck: split specimens, exclusions and partial sequencing. Overlap: Distinct from Bao et al. 2018
OMD-2019-Camacaro [67]	Unclear / Unclear / Unclear / High / Unclear	S: Recruitment sequence, consecutive enrollment and the complete exclusion process are not established by the available extraction. 49 patients with clinical tinea unguium plus 10 healthy controls. I: Prospective threshold selection and blinded interpretation are not established by the available assessment record. Molecular endpoint: Pan-dermatophyte CHS1 plus *T. rubrum* and *T. mentagrophytes* ITS targets.	C: The extracted endpoint or its handling has unresolved reporting/target-scope qualifications. Culture treated as author gold standard. F: PCR and conventional tests used different nail locations; a reported sensitivity is incompatible with the integer table reconstructed from the remaining data. A: Applicability depends on treatment, sampling, target or subgroup qualifications: Small cohort; different nail portions/locations; no direct cross-tab; reported 97% sensitivity is incompatible with the discrete reconstructed table (100%). Treatment exposure: 51% stopped topical/systemic antifungals one month before evaluation; 42.9% reported no use; 6.1% unclear	Workbook v0.12 Evidence_Table row 44; Full_Text_Screening record OMD-2019-Camacaro; source locator: Full text Results and reported performance. Targeted revision PDF recheck: different sampling sites and previous treatment. Overlap: None identified
OMD-2013-Chandran [27]	High / Unclear / Low / Low / High	S: Entry required positive direct microscopy, verified in the full-text methods. I: Prospective threshold selection and blinded interpretation are not established by the available assessment record. Molecular endpoint: *T. rubrum* plus pan-dermatophyte.	C: For raw paired discordance, the comparator is interpreted as a separate detection method rather than disease truth. Microscopy-positive entry spectrum; no independent resolver. F: The extracted clinical/nail endpoint has an exact paired denominator; no additional selective verification or unresolved invalid-result loss is documented for those raw cells. Diseased nail divided into microscopy, culture and PCR portions. A: Restricted applicability to unselected, first-line suspected onychomycosis: Microscopy-selected prospective cohort. 99 patients with clinically suspected OM; 107 microscopy-positive nail specimens	Workbook v0.12 Evidence_Table row 45; Full_Text_Screening record OMD-2013-Chandran; source locator: Full text Figure 2 and Results. Targeted revision PDF recheck: entry restricted to direct-microscopy-positive nails. Overlap: None identified
OMD-2022-Gupta-Therapy [42]	High / Unclear / Low / High / High	S: PCR was performed in a treatment-study completer subset with stored baseline and month-24 material. I: Prospective threshold selection and blinded interpretation are not established by the available assessment record. Molecular endpoint: *T. rubrum*, *T. mentagrophytes* complex and selected NDMs.	C: For raw paired discordance, the comparator is interpreted as a separate detection method rather than disease truth. Timepoint-specific comparison; visual clearance is not a conventional comparator. F: Selected month-24 completers, repeated measures and stored-material availability govern the analyzed subset. A: Restricted applicability to unselected, first-line suspected onychomycosis: Treatment monitoring. 28 culture-confirmed participants completing topical efinaconazole follow-up to month 24	Workbook v0.12 Evidence_Table row 46; Full_Text_Screening record OMD-2022-Gupta-Therapy; source locator: Full text Tables 1-2. Targeted revision PDF recheck: selected treatment-study completers and stored specimens. Overlap: Possible source-trial overlap; no duplicate report identified
OMD-2014-Dhib [14]	Unclear / Unclear / Low / Low / Low	S: Recruitment sequence, consecutive enrollment and the complete exclusion process are not established by the available extraction. 201 suspected onychomycosis toenail specimens. I: Prospective threshold selection and blinded interpretation are not established by the available assessment record. Analytical cross-amplification affects species assignment but not necessarily the pan-dermatophyte binary endpoint.	C: For raw paired discordance, the comparator is interpreted as a separate detection method rather than disease truth. No independent resolver. F: The extracted clinical/nail endpoint has an exact paired denominator; no additional selective verification or unresolved invalid-result loss is documented for those raw cells. Specimens divided for direct examination, culture and multiplex PCR. A: Clinical nail comparison applicable within its stated organism target and setting; it does not establish all-pathogen accuracy or clinical benefit. Tunisia; clinical mycology laboratory	Workbook v0.12 Evidence_Table row 47; Full_Text_Screening record OMD-2014-Dhib; source locator: Full text Table 3. Targeted revision PDF recheck: clinical/control sampling and analytical cross-reactivity. Overlap: Probable overlap: same Sousse group, 201 suspected nails and 80 controls, identical conventional marginals (microscopy 151, culture 132) in Dhib2012/2014; recruitment linkage not confirmed. Conservatively test one-report retention.
OMD-2020-Caldwell [17]	Unclear / Unclear / Low / Low / Unclear	S: Recruitment sequence, consecutive enrollment and the complete exclusion process are not established by the available extraction. 209 clinically suspected hallux OM patients; 203 with adequate paired samples. I: Prospective threshold selection and blinded interpretation are not established by the available assessment record. Molecular endpoint: Dermatophytes, selected molds and *C. albicans*.	C: For raw paired discordance, the comparator is interpreted as a separate detection method rather than disease truth. No independent resolver. F: The extracted clinical/nail endpoint has an exact paired denominator; no additional selective verification or unresolved invalid-result loss is documented for those raw cells. Proximal hallux clipping/debris split equally; PCR and PAS sent to separate commercial laboratories. A: Applicability depends on treatment, sampling, target or subgroup qualifications: Severe-spectrum cohort; no culture/resolver; different laboratories; targeted PCR cannot detect organisms outside its panel.	Workbook v0.12 Evidence_Table row 48; Full_Text_Screening record OMD-2020-Caldwell; source locator: Full text Table 1. Targeted revision PDF recheck: treatment exclusion and inadequate paired material. Overlap: Possible commercial laboratory family; not demonstrated
OMD-2023-Debuysschere [15]	High / Unclear / Low / High / Unclear	S: Of 320 submitted specimens, 189 were excluded for insufficient material or unsuitable transport, leaving 131 paired samples; this restricts the submitted-sample spectrum. I: Prospective threshold selection and blinded interpretation are not established by the available assessment record. Molecular endpoint: Dermatophyte complexes/species.	C: For raw paired discordance, the comparator is interpreted as a separate detection method rather than disease truth. DermaGenius applied to PCR/culture-discordant samples. F: A large preanalytic exclusion fraction (189/320) is documented; all 131 retained specimens have paired raw culture/PCR results. Selective molecular resolver is separate. A: Applicability depends on treatment, sampling, target or subgroup qualifications: Molecular resolver is not independent of the index principle; very low direct-examination positivity; no clinical outcome adjudication.	Workbook v0.12 Evidence_Table row 49; Full_Text_Screening record OMD-2023-Debuysschere; source locator: Full text Results and Figure 1. Targeted revision PDF recheck: preanalytic exclusions, paired sample flow and selective resolver. Overlap: None identified
OMD-2017-Hafirassou [37]	Unclear / Unclear / Low / Low / Low	S: Recruitment sequence, consecutive enrollment and the complete exclusion process are not established by the available extraction. 70 clinical OM nail samples and 15 healthy volunteer nails. I: Prospective threshold selection and blinded interpretation are not established by the available assessment record. Raw panfungal amplification and sequence-confirmed identification differ; follow-on organism-specific assays were applied selectively.	C: For raw paired discordance, the comparator is interpreted as a separate detection method rather than disease truth. Amplicon sequencing for panfungal/pandermatophyte assays. F: The extracted clinical/nail endpoint has an exact paired denominator; no additional selective verification or unresolved invalid-result loss is documented for those raw cells. Clinical nails tested by conventional methods, panfungal and pandermatophyte RT-PCR; selected samples received Candida/Aspergillus assays. A: Clinical nail comparison applicable within its stated organism target and setting; it does not establish all-pathogen accuracy or clinical benefit. Algeria/Spain; Constantine Hospital samples with reference-laboratory molecular testing	Workbook v0.12 Evidence_Table row 50; Full_Text_Screening record OMD-2017-Hafirassou; source locator: Full text Tables 1-4. Targeted revision PDF recheck: clinical sample allocation and assay-specific targets. Overlap: None identified
OMD-2016-Gong [26]	High / Unclear / Low / Low / High	S: The 165-sample analysis combines 70 clinically suspected nails with 95 asymptomatic nails/controls. I: Prospective threshold selection and blinded interpretation are not established by the available assessment record. Molecular endpoint: Pan-dermatophyte and *T. rubrum*.	C: For raw paired discordance, the comparator is interpreted as a separate detection method rather than disease truth. No gold standard; latent-class model additionally reported. F: The extracted clinical/nail endpoint has an exact paired denominator; no additional selective verification or unresolved invalid-result loss is documented for those raw cells. Nails tested by microscopy, culture, conventional PCR and novel RT-PCR. A: Restricted applicability to unselected, first-line suspected onychomycosis: Case/control spectrum plus latent-class analysis. 165 nails: 70 with suspected mycosis and 95 without obvious symptoms	Workbook v0.12 Evidence_Table row 51; Full_Text_Screening record OMD-2016-Gong; source locator: Full text Tables 3-4. Targeted revision PDF recheck: symptomatic/asymptomatic recruitment and internal control. Overlap: None identified
OMD-2019-Hayette [19]	Unclear / Unclear / Low / High / Unclear	S: Recruitment sequence, consecutive enrollment and the complete exclusion process are not established by the available extraction. 138 archived finger/toenail clippings. I: Prospective threshold selection and blinded interpretation are not established by the available assessment record. Manufacturer instructions and internal control were used; eight inhibited results require explicit invalid-state handling.	C: For raw paired discordance, the comparator is interpreted as a separate detection method rather than disease truth. Conventional composite independent of index PCR; ITS sequencing for selected species discrepancies. F: The source-reported N=138 tables include 8 inhibited PCR results among negatives. Valid-only target-culture cells can be reconstructed (N=130); valid-only histology/composite cells cannot. A: Applicability depends on treatment, sampling, target or subgroup qualifications: Archived material; eight inhibited PCRs; panel excludes NDMs; reference definitions yield different conclusions. Revision recheck: patients were first consultations without previous treatment; workbook treatment status was incomplete.	Workbook v0.12 Evidence_Table row 52; Full_Text_Screening record OMD-2019-Hayette; source locator: Full text Tables 1-4. Targeted revision PDF recheck: absence of prior treatment and inhibited PCR handling (printed pp. 278-280). Overlap: None identified
OMD-2011-Berk [68]	Unclear / Unclear / Low / Low / Low	S: Recruitment sequence, consecutive enrollment and the complete exclusion process are not established by the available extraction. 90 patients with suspected OM and 20 healthy volunteers. I: Prospective threshold selection and blinded interpretation are not established by the available assessment record. Molecular endpoint: *T. rubrum*-specific.	C: For raw paired discordance, the comparator is interpreted as a separate detection method rather than disease truth. Healthy controls evaluated separately; no resolver. F: The extracted clinical/nail endpoint has an exact paired denominator; no additional selective verification or unresolved invalid-result loss is documented for those raw cells. Direct nail scrapings tested by KOH-based microscopy, culture and *T. rubrum* qPCR. A: Clinical nail comparison applicable within its stated organism target and setting; it does not establish all-pathogen accuracy or clinical benefit. Turkey; hospital dermatology clinic	Workbook v0.12 Evidence_Table row 53; Full_Text_Screening record OMD-2011-Berk; source locator: English abstract and full-text results. Targeted revision PDF recheck: English abstract only; full body poorly text-extractable. Overlap: None identified
OMD-2026-Khoo [69]	High / Unclear / Low / Unclear / High	S: All entrants were direct-microscopy-positive; the report also combines nail and non-nail sites. I: Prospective threshold selection and blinded interpretation are not established by the available assessment record. Molecular endpoint: Dermatophytes, selected yeasts and molds.	C: For raw paired discordance, the comparator is interpreted as a separate detection method rather than disease truth. No independent resolver. F: Completeness of valid, same-specimen paired observations is not fully established for all reported analyses. Mixed sites; microscopy-positive selection; no complete nail cross-tab; small sample; assay sponsorship. A: Restricted applicability to unselected, first-line suspected onychomycosis: Mixed-site microscopy-positive cohort. 61 analyzable superficial fungal infection specimens: 50 nails, 10 skin, 1 hair	Workbook v0.12 Evidence_Table row 54; Full_Text_Screening record OMD-2026-Khoo; source locator: Full text Results and Tables 1-2. Targeted revision PDF recheck: microscopy-positive inclusion and discarded specimens. Overlap: Same institution as Chandran, but study periods 2021-2022 versus 2011-2012
OMD-2010-Brillowska-Routine [12]	Unclear / Unclear / Low / Low / Low	S: Recruitment sequence, consecutive enrollment and the complete exclusion process are not established by the available extraction. 109 nail specimens submitted for suspected tinea unguium. I: Prospective threshold selection and blinded interpretation are not established by the available assessment record. An internal inhibition control and reduced-template repeat rule are documented; blinded interpretation is not established.	C: For raw paired discordance, the comparator is interpreted as a separate detection method rather than disease truth. No independent resolver; explicit inhibitor repeat rule. F: The extracted clinical/nail endpoint has an exact paired denominator; no additional selective verification or unresolved invalid-result loss is documented for those raw cells. Material allocated to microscopy/culture and PCR; inhibited PCRs repeated at half template volume. A: Clinical nail comparison applicable within its stated organism target and setting; it does not establish all-pathogen accuracy or clinical benefit. Denmark; routine reference PCR laboratory	Workbook v0.12 Evidence_Table row 55; Full_Text_Screening record OMD-2010-Brillowska-Routine; source locator: Full text Table 1. Targeted revision PDF recheck: material-adequacy inclusion and inhibitor repeat rule. Overlap: Assay-development lineage with 2007 paper; clinical cohort appears distinct
OMD-2010-Kondori [13]	Unclear / Unclear / Low / Low / Low	S: Recruitment sequence, consecutive enrollment and the complete exclusion process are not established by the available extraction. 177 nail specimens with suspected onychomycosis. I: Prospective threshold selection and blinded interpretation are not established by the available assessment record. Molecular endpoint: Pan-dermatophyte plus *T. rubrum*.	C: For raw paired discordance, the comparator is interpreted as a separate detection method rather than disease truth. No independent resolver. F: The extracted clinical/nail endpoint has an exact paired denominator; no additional selective verification or unresolved invalid-result loss is documented for those raw cells. Nails divided into fluorescence microscopy, culture and duplex PCR portions. A: Clinical nail comparison applicable within its stated organism target and setting; it does not establish all-pathogen accuracy or clinical benefit. Sweden; Gothenburg clinical laboratory	Workbook v0.12 Evidence_Table row 56; Full_Text_Screening record OMD-2010-Kondori; source locator: Full text Table 1. Targeted revision PDF recheck: aliquot allocation and inhibition control. Overlap: None identified
OMD-2014-Dubljanin [24]	Unclear / Unclear / Low / Unclear / Low	S: Recruitment sequence, consecutive enrollment and the complete exclusion process are not established by the available extraction. 183 patients with suspected toenail onychomycosis. I: Prospective threshold selection and blinded interpretation are not established by the available assessment record. Internal inhibition control and a dilution/retest procedure are described; blinded interpretation is not established.	C: For raw paired discordance, the comparator is interpreted as a separate detection method rather than disease truth. No independent resolver. F: Completeness of valid, same-specimen paired observations is not fully established for all reported analyses. Table/text overlap inconsistency; set intersections algebraically reconstructed; dermatophyte-only target; no resolver. A: Clinical nail comparison applicable within its stated organism target and setting; it does not establish all-pathogen accuracy or clinical benefit. Serbia; University of Belgrade clinical cohort with SSI molecular collaboration	Workbook v0.12 Evidence_Table row 57; Full_Text_Screening record OMD-2014-Dubljanin; source locator: Full text Table 1 and Results. Targeted revision PDF recheck: six-month treatment washout and inhibitor repeat rule. Overlap: None identified
OMD-2026-Caza [33]	Unclear / Unclear / High / High / Unclear	S: Recruitment sequence, consecutive enrollment and the complete exclusion process are not established by the available extraction. 149 residual nail samples submitted for KOH and/or fungal culture: 65 toenails, 4 fingernails and 80 unspecified nails. I: Prospective threshold selection and blinded interpretation are not established by the available assessment record. Molecular endpoint: *T. rubrum* complex, *T. mentagrophytes* complex, *T. tonsurans*, *Microsporum* spp., *E. floccosum* and *C. albicans*.	C: High for consensus/adjudicated rows incorporating PCR and selectively applied sequencing; specimen-level raw culture data remain a separate endpoint. F: Four KOH results are missing; residual-material availability and variable storage affect inclusion; selected molecular adjudication and target-level repeated determinations require separate handling. A: Applicability depends on treatment, sampling, target or subgroup qualifications: Retrospective residual samples; variable storage; no clinical correlation; 4 missing KOH results; selective molecular resolver; sample-level cells reconstructed from margins/agreement; target-level analyses are not independent nails; panel omits most non-dermatophyte fungi and resistance markers; contrived-nail experiments are analytical only.	Workbook v0.12 Evidence_Table row 58; Full_Text_Screening record OMD-2026-Caza; source locator: Full text Tables 1 and 3-5, Figure 1, Results, Discussion and supplement. Existing full-text extraction only; source PDF not re-examined for this appraisal. Overlap: None identified
OMD-2025-Tirard-Collet [39]	Unclear / Unclear / Unclear / High / Unclear	S: Recruitment sequence, consecutive enrollment and the complete exclusion process are not established by the available extraction. 85 clinical samples: 50 skin scrapings, stated 29 nails and 6 hair samples; Table 1 accounts for 52 skin, 27 nail and 6 hair samples. I: Prospective threshold selection and blinded interpretation are not established by the available assessment record. Molecular endpoint: *C. albicans* and 12 dermatophyte targets.	C: The extracted endpoint or its handling has unresolved reporting/target-scope qualifications. Selective panfungal MITS 2a/2b PCR and Sanger sequencing for unexpected kit identifications. F: Stated and tabulated nail denominators disagree; two invalid PCRs and two additional nails are unaccounted for. Only the supportive interpretable subset is used. A: Applicability depends on treatment, sampling, target or subgroup qualifications: Mixed-site retrospective selection; stated 29 nails versus 27 tabulated; two invalid nail PCRs and two additional nails unaccounted; selective resolver; panel excludes most NDMs.	Workbook v0.12 Evidence_Table row 59; Full_Text_Screening record OMD-2025-Tirard-Collet; source locator: Full text Table 1, Results and Supplementary Table S1. Existing full-text extraction only; source PDF not re-examined for this appraisal. Overlap: None identified
OMD-2025-Kharibam [40]	High / Unclear / Low / Low / High	S: PCR comparison restricted from 350 suspected patients to a KOH-positive, dermatophyte-classified subset of 70. I: Prospective threshold selection and blinded interpretation are not established by the available assessment record. Molecular endpoint: *T. rubrum* and *T. mentagrophytes* only.	C: For raw paired discordance, the comparator is interpreted as a separate detection method rather than disease truth. No independent resolver. F: The extracted clinical/nail endpoint has an exact paired denominator; no additional selective verification or unresolved invalid-result loss is documented for those raw cells. Finger- and toenail material divided for 40% KOH, culture and PCR. A: Restricted applicability to unselected, first-line suspected onychomycosis: KOH-positive enriched clinical nail cohort. 350 clinically suspected patients screened; 100 KOH-positive, of whom 70 were classified as dermatophyte-positive and entered PCR comparison	Workbook v0.12 Evidence_Table row 60; Full_Text_Screening record OMD-2025-Kharibam; source locator: Full text Table 4 and Results. Existing full-text extraction only; source PDF not re-examined for this appraisal. Overlap: None identified
OMD-2020-Gill [38]	High / Unclear / Low / Low / High	S: The small cohort is described as fungal dystrophic nails and contains no doubly negative pair; the disease spectrum is strongly enriched. I: Prospective threshold selection and blinded interpretation are not established by the available assessment record. Molecular endpoint: Band-pattern identification of *T. rubrum* and *T. mentagrophytes*; nondermatophytes off-target.	C: For raw paired discordance, the comparator is interpreted as a separate detection method rather than disease truth. No independent resolver. F: The extracted clinical/nail endpoint has an exact paired denominator; no additional selective verification or unresolved invalid-result loss is documented for those raw cells. Nail material divided for 20% KOH, Sabouraud culture and direct AP-PCR. A: Restricted applicability to unselected, first-line suspected onychomycosis: Enriched dystrophic-nail cohort. 48 patients with dystrophic fungal nail infection	Workbook v0.12 Evidence_Table row 61; Full_Text_Screening record OMD-2020-Gill; source locator: Full text Table 2 and Results. Existing full-text extraction only; source PDF not re-examined for this appraisal. Overlap: None identified
OMD-2008-Gupta-Treated [70]	Unclear / Unclear / Low / Low / High	S: Recruitment sequence, consecutive enrollment and the complete exclusion process are not established by the available extraction. 83 patients with onychomycosis receiving antifungal therapy. I: Prospective threshold selection and blinded interpretation are not established by the available assessment record. Molecular endpoint: *T. rubrum* actin gene.	C: For raw paired discordance, the comparator is interpreted as a separate detection method rather than disease truth. No independent resolver. F: The extracted clinical/nail endpoint has an exact paired denominator; no additional selective verification or unresolved invalid-result loss is documented for those raw cells. Nail material assessed by calcofluor microscopy, culture and PCR. A: Restricted applicability to unselected, first-line suspected onychomycosis: Treated cohort. 83 patients with onychomycosis receiving antifungal therapy All patients were receiving antifungal therapy.	Workbook v0.12 Evidence_Table row 62; Full_Text_Screening record OMD-2008-Gupta-Treated; source locator: Full text combination table and Results. Existing full-text extraction only; source PDF not re-examined for this appraisal. Overlap: None identified
OMD-2014-Iglesias-Sanchez [71]	Unclear / Unclear / Low / Low / Low	S: Recruitment sequence, consecutive enrollment and the complete exclusion process are not established by the available extraction. 232 suspected nail fungal infections; 225 had sufficient material for PCR. I: Prospective threshold selection and blinded interpretation are not established by the available assessment record. Molecular endpoint: Pan-dermatophyte CHS1 plus *T. rubrum*-specific target.	C: For raw paired discordance, the comparator is interpreted as a separate detection method rather than disease truth. No independent resolver. F: The extracted clinical/nail endpoint has an exact paired denominator; no additional selective verification or unresolved invalid-result loss is documented for those raw cells. Fragments of the same nail used for culture and nested PCR; seven insufficient for PCR. A: Clinical nail comparison applicable within its stated organism target and setting; it does not establish all-pathogen accuracy or clinical benefit. Spain; three clinical centres in Cáceres province	Workbook v0.12 Evidence_Table row 63; Full_Text_Screening record OMD-2014-Iglesias-Sanchez; source locator: Full text Table 1 and Results. Existing full-text extraction only; source PDF not re-examined for this appraisal. Overlap: None identified
OMD-2015-Spiliopoulou [72]	Unclear / Unclear / Low / Low / Low	S: Recruitment sequence, consecutive enrollment and the complete exclusion process are not established by the available extraction. 418 nail scrapings from dermatology outpatients. I: Prospective threshold selection and blinded interpretation are not established by the available assessment record. Molecular endpoint: Pan-dermatophyte with *T. rubrum* identification.	C: For raw paired discordance, the comparator is interpreted as a separate detection method rather than disease truth. No independent resolver. F: The extracted clinical/nail endpoint has an exact paired denominator; no additional selective verification or unresolved invalid-result loss is documented for those raw cells. Routine nail scrapings processed by microscopy, culture and multiplex PCR. A: Clinical nail comparison applicable within its stated organism target and setting; it does not establish all-pathogen accuracy or clinical benefit. Greece; dermatology outpatient laboratory	Workbook v0.12 Evidence_Table row 64; Full_Text_Screening record OMD-2015-Spiliopoulou; source locator: Full text combination table and Results. Existing full-text extraction only; source PDF not re-examined for this appraisal. Overlap: None identified
OMD-2023-Navarro-Perez-Culture [73]	Unclear / Unclear / Low / Low / Unclear	S: Recruitment sequence, consecutive enrollment and the complete exclusion process are not established by the available extraction. 121 patients with suspected onychomycosis and both culture and PCR. I: Prospective threshold selection and blinded interpretation are not established by the available assessment record. Molecular endpoint: Dermatophyte positive/negative report; culture broader.	C: For raw paired discordance, the comparator is interpreted as a separate detection method rather than disease truth. Culture treated analytically as reference; no independent resolver. F: The extracted clinical/nail endpoint has an exact paired denominator; no additional selective verification or unresolved invalid-result loss is documented for those raw cells. Nail portions large enough for both methods; external laboratory culture. A: Applicability depends on treatment, sampling, target or subgroup qualifications: Retrospective, no independent resolver, target mismatch, limited comorbidity data	Workbook v0.12 Evidence_Table row 65; Full_Text_Screening record OMD-2023-Navarro-Perez-Culture; source locator: Full text Table 3. Existing full-text extraction only; source PDF not re-examined for this appraisal. Overlap: Possible author-group overlap with later study; distinct data set
OMD-2020-Mohtady [74]	Unclear / Unclear / Unclear / Unclear / Low	S: Recruitment sequence, consecutive enrollment and the complete exclusion process are not established by the available extraction. 61 patients with suspected onychomycosis. I: Prospective threshold selection and blinded interpretation are not established by the available assessment record. Molecular endpoint: Dermatophyte detection.	C: The extracted endpoint or its handling has unresolved reporting/target-scope qualifications. Culture used as reference. F: Completeness of valid, same-specimen paired observations is not fully established for all reported analyses. Small cohort, culture-reference assumption, internal marginal-label inconsistency. A: Clinical nail comparison applicable within its stated organism target and setting; it does not establish all-pathogen accuracy or clinical benefit. Egypt; university clinical cohort	Workbook v0.12 Evidence_Table row 66; Full_Text_Screening record OMD-2020-Mohtady; source locator: Full text diagnostic-validity cross-table. Existing full-text extraction only; source PDF not re-examined for this appraisal. Overlap: None identified
OMD-2017-Motamedi [75]	Unclear / Unclear / Low / High / High	S: Recruitment sequence, consecutive enrollment and the complete exclusion process are not established by the available extraction. 853 suspected dermatophyte lesions; 538 nail specimens; culture available for a subset of the overall cohort. I: Prospective threshold selection and blinded interpretation are not established by the available assessment record. Molecular endpoint: Pan-dermatophyte beta-tubulin.	C: For raw paired discordance, the comparator is interpreted as a separate detection method rather than disease truth. No independent resolver. F: Culture was applied only to a subset, with no exact nail-specific cross-table or valid paired denominator. A: Restricted applicability to unselected, first-line suspected onychomycosis: Mixed-site cohort with nail-specific performance metrics. 853 suspected dermatophyte lesions; 538 nail specimens; culture available for a subset of the overall cohort	Workbook v0.12 Evidence_Table row 67; Full_Text_Screening record OMD-2017-Motamedi; source locator: Full text subgroup performance table and Results. Existing full-text extraction only; source PDF not re-examined for this appraisal. Overlap: Related assay-development group; distinct clinical cohort
OMD-2016-Mirhendi [76]	High / Unclear / Unclear / Unclear / High	S: Clinical validation specimens were selected to be microscopy-positive and/or culture-positive; controls were recruited separately. I: Prospective threshold selection and blinded interpretation are not established by the available assessment record. Molecular endpoint: Pan-dermatophyte beta-tubulin.	C: The extracted endpoint or its handling has unresolved reporting/target-scope qualifications. Eligibility enrichment by either conventional test. F: Completeness of valid, same-specimen paired observations is not fully established for all reported analyses. Verification-enriched positive spectrum, mixed sites, nail-specific paired cells unavailable. A: Restricted applicability to unselected, first-line suspected onychomycosis: Verification-enriched mixed-site validation. 234 clinical specimens selected as positive by microscopy and/or culture across skin, nail and hair, including 80 nail specimens; 32 healthy nail controls	Workbook v0.12 Evidence_Table row 68; Full_Text_Screening record OMD-2016-Mirhendi; source locator: Full text Methods and Results. Existing full-text extraction only; source PDF not re-examined for this appraisal. Overlap: Related to later Motamedi clinical validation; no duplicate cohort established
OMD-2016-Emam [77]	Unclear / Unclear / Unclear / Unclear / Unclear	S: Recruitment sequence, consecutive enrollment and the complete exclusion process are not established by the available extraction. 80 patients with clinically suspected dermatophyte onychomycosis. I: Prospective threshold selection and blinded interpretation are not established by the available assessment record. Molecular endpoint: Dermatophyte detection/species targets.	C: The extracted endpoint or its handling has unresolved reporting/target-scope qualifications. Dermatophyte culture used as reference. F: Completeness of valid, same-specimen paired observations is not fully established for all reported analyses. Cells reconstructed from rounded diagnostic metrics and exact marginals; culture-reference assumption. A: Applicability depends on treatment, sampling, target or subgroup qualifications: Cells reconstructed from rounded diagnostic metrics and exact marginals; culture-reference assumption	Workbook v0.12 Evidence_Table row 69; Full_Text_Screening record OMD-2016-Emam; source locator: Full text tables and Results. Existing full-text extraction only; source PDF not re-examined for this appraisal. Overlap: None identified
OMD-2012-Moallemzadeh [78]	Unclear / Unclear / Unclear / Unclear / Low	S: Recruitment sequence, consecutive enrollment and the complete exclusion process are not established by the available extraction. 71 nail samples from patients with suspected tinea unguium. I: Prospective threshold selection and blinded interpretation are not established by the available assessment record. Molecular endpoint: Universal fungal and *T. rubrum*-specific endpoints.	C: The extracted endpoint or its handling has unresolved reporting/target-scope qualifications. No independent resolver. F: Completeness of valid, same-specimen paired observations is not fully established for all reported analyses. Marginal-only reporting, differing target definitions, incomplete bibliographic indexing. A: Clinical nail comparison applicable within its stated organism target and setting; it does not establish all-pathogen accuracy or clinical benefit. Iran; clinical nail cohort	Workbook v0.12 Evidence_Table row 70; Full_Text_Screening record OMD-2012-Moallemzadeh; source locator: Full text Results. Existing full-text extraction only; source PDF not re-examined for this appraisal. Overlap: None identified
OMD-2011-Li [25]	Unclear / Unclear / High / Unclear / Low	S: Recruitment sequence, consecutive enrollment and the complete exclusion process are not established by the available extraction. Yes-n=104. I: Prospective threshold selection and blinded interpretation are not established by the available assessment record. Molecular endpoint: Dermatophytes, yeasts and molds.	C: The study-defined disease standard requires two of PCR, microscopy and culture and therefore incorporates the index; only raw comparator-specific rows support discordance summaries. F: Completeness of valid, same-specimen paired observations is not fully established for all reported analyses. No independent gold standard; comparator/target mismatch and spectrum selection handled in separate strata. A: Clinical nail comparison applicable within its stated organism target and setting; it does not establish all-pathogen accuracy or clinical benefit. China	Workbook v0.12 Evidence_Table row 71; Full_Text_Screening record OMD-2011-Li; source locator: Full text Table 1 and Results. Existing full-text extraction only; source PDF not re-examined for this appraisal. Overlap: No
OMD-2004-Arca [79]	Unclear / Unclear / Low / Unclear / Low	S: Recruitment sequence, consecutive enrollment and the complete exclusion process are not established by the available extraction. Yes-n=52. I: Prospective threshold selection and blinded interpretation are not established by the available assessment record. Molecular endpoint: Fungal detection.	C: For raw paired discordance, the comparator is interpreted as a separate detection method rather than disease truth. No independent resolver unless stated; paired method comparison. F: Completeness of valid, same-specimen paired observations is not fully established for all reported analyses. No independent gold standard; comparator/target mismatch and spectrum selection handled in separate strata. A: Clinical nail comparison applicable within its stated organism target and setting; it does not establish all-pathogen accuracy or clinical benefit. Turkey	Workbook v0.12 Evidence_Table row 72; Full_Text_Screening record OMD-2004-Arca; source locator: Full text Results. Existing full-text extraction only; source PDF not re-examined for this appraisal. Overlap: No
OMD-2011-Beifuss [80]	Unclear / Unclear / Unclear / Unclear / High	S: Recruitment sequence, consecutive enrollment and the complete exclusion process are not established by the available extraction. Nail strata reported, but no nail-specific paired cross-table. I: Prospective threshold selection and blinded interpretation are not established by the available assessment record. Molecular endpoint: Five dermatophyte species.	C: The extracted endpoint or its handling has unresolved reporting/target-scope qualifications. No independent resolver unless stated; paired method comparison. F: Completeness of valid, same-specimen paired observations is not fully established for all reported analyses. Exact nail-specific paired binary cells unavailable or invalid/species states prevent reconstruction. A: Restricted applicability to unselected, first-line suspected onychomycosis: Narrative only. Nail strata reported, but no nail-specific paired cross-table	Workbook v0.12 Evidence_Table row 73; Full_Text_Screening record OMD-2011-Beifuss; source locator: Full text Tables 2-3. Existing full-text extraction only; source PDF not re-examined for this appraisal. Overlap: No duplicate cohort established
OMD-2009-Uchida [81]	Unclear / Unclear / Unclear / Unclear / High	S: Recruitment sequence, consecutive enrollment and the complete exclusion process are not established by the available extraction. Yes-80 nails; paired marginals only. I: Prospective threshold selection and blinded interpretation are not established by the available assessment record. Molecular endpoint: Dermatophytes with species differentiation.	C: The extracted endpoint or its handling has unresolved reporting/target-scope qualifications. No independent resolver unless stated; paired method comparison. F: Completeness of valid, same-specimen paired observations is not fully established for all reported analyses. Exact nail-specific paired binary cells unavailable or invalid/species states prevent reconstruction. A: Restricted applicability to unselected, first-line suspected onychomycosis: Narrative only. Yes-80 nails; paired marginals only	Workbook v0.12 Evidence_Table row 74; Full_Text_Screening record OMD-2009-Uchida; source locator: Full text nail subgroup table. Existing full-text extraction only; source PDF not re-examined for this appraisal. Overlap: No
OMD-2022-Trave [82]	Unclear / Unclear / Low / Low / Low	S: Recruitment sequence, consecutive enrollment and the complete exclusion process are not established by the available extraction. Yes-n=100. I: Prospective threshold selection and blinded interpretation are not established by the available assessment record. Molecular endpoint: Dermatophytes and selected fungi.	C: For raw paired discordance, the comparator is interpreted as a separate detection method rather than disease truth. No independent resolver unless stated; paired method comparison. F: The extracted clinical/nail endpoint has an exact paired denominator; no additional selective verification or unresolved invalid-result loss is documented for those raw cells. Primary nail material divided or sampled for molecular and conventional testing; see source-specific notes. A: Clinical nail comparison applicable within its stated organism target and setting; it does not establish all-pathogen accuracy or clinical benefit. Italy; Genoa	Workbook v0.12 Evidence_Table row 75; Full_Text_Screening record OMD-2022-Trave; source locator: Full text Table 1. Existing full-text extraction only; source PDF not re-examined for this appraisal. Overlap: No
OMD-2017-Gupta-Nakrieko [83]	Unclear / Unclear / Unclear / Unclear / Unclear	S: Recruitment sequence, consecutive enrollment and the complete exclusion process are not established by the available extraction. Yes-167 single samples plus 43 serial-sample patient sets. I: Prospective threshold selection and blinded interpretation are not established by the available assessment record. Molecular endpoint: 16 dermatophytes and five nondermatophyte molds.	C: The extracted endpoint or its handling has unresolved reporting/target-scope qualifications. No independent resolver unless stated; paired method comparison. F: Completeness of valid, same-specimen paired observations is not fully established for all reported analyses. Exact nail-specific paired binary cells unavailable or invalid/species states prevent reconstruction. A: Applicability depends on treatment, sampling, target or subgroup qualifications: Exact nail-specific paired binary cells unavailable or invalid/species states prevent reconstruction.	Workbook v0.12 Evidence_Table row 76; Full_Text_Screening record OMD-2017-Gupta-Nakrieko; source locator: Full text agreement tables. Existing full-text extraction only; source PDF not re-examined for this appraisal. Overlap: No
OMD-2019-Watanabe-LAMP [84]	High / Unclear / Low / Low / High	S: All clinical nails were microscopy-positive. I: Prospective threshold selection and blinded interpretation are not established by the available assessment record. Molecular endpoint: *T. rubrum* and *T. interdigitale.*	C: For raw paired discordance, the comparator is interpreted as a separate detection method rather than disease truth. No independent resolver unless stated; paired method comparison. F: The extracted clinical/nail endpoint has an exact paired denominator; no additional selective verification or unresolved invalid-result loss is documented for those raw cells. Primary nail material divided or sampled for molecular and conventional testing; see source-specific notes. A: Restricted applicability to unselected, first-line suspected onychomycosis: Paired 2 × 2. Yes-n=90	Workbook v0.12 Evidence_Table row 77; Full_Text_Screening record OMD-2019-Watanabe-LAMP; source locator: Full text Table 4. Existing full-text extraction only; source PDF not re-examined for this appraisal. Overlap: No
OMD-2016-Petinataud [85]	Unclear / Unclear / Low / High / Unclear	S: Recruitment sequence, consecutive enrollment and the complete exclusion process are not established by the available extraction. Yes-90 patients; 180 nail samples. I: Prospective threshold selection and blinded interpretation are not established by the available assessment record. Molecular endpoint: Dermatophytes.	C: For raw paired discordance, the comparator is interpreted as a separate detection method rather than disease truth. No independent resolver unless stated; paired method comparison. F: Optimal and non-optimal samples come from the same 90 patients and cannot be counted as independent observations in a combined summary. A: Applicability depends on treatment, sampling, target or subgroup qualifications: No independent gold standard; comparator/target mismatch and spectrum selection handled in separate strata.	Workbook v0.12 Evidence_Table row 78; Full_Text_Screening record OMD-2016-Petinataud; source locator: Full text Table 4. Existing full-text extraction only; source PDF not re-examined for this appraisal. Overlap: Optimal and non-optimal samples are paired within the same 90 patients
OMD-2013-Paugam [86]	Unclear / Unclear / Unclear / Unclear / High	S: Recruitment sequence, consecutive enrollment and the complete exclusion process are not established by the available extraction. Yes-54 nails, two PCR uninterpretable; metrics only. I: Prospective threshold selection and blinded interpretation are not established by the available assessment record. Molecular endpoint: Dermatophytes.	C: The extracted endpoint or its handling has unresolved reporting/target-scope qualifications. No independent resolver unless stated; paired method comparison. F: Completeness of valid, same-specimen paired observations is not fully established for all reported analyses. Exact nail-specific paired binary cells unavailable or invalid/species states prevent reconstruction. A: Restricted applicability to unselected, first-line suspected onychomycosis: Narrative only. Yes-54 nails, two PCR uninterpretable; metrics only	Workbook v0.12 Evidence_Table row 79; Full_Text_Screening record OMD-2013-Paugam; source locator: Full text nail subgroup results. Existing full-text extraction only; source PDF not re-examined for this appraisal. Overlap: No
OMD-2013-Winter [87]	Unclear / Unclear / Low / Unclear / High	S: Recruitment sequence, consecutive enrollment and the complete exclusion process are not established by the available extraction. Yes-218 nail specimens; marginals only. I: Prospective threshold selection and blinded interpretation are not established by the available assessment record. Molecular endpoint: *T. rubrum*, *T. interdigitale*, *E. floccosum*.	C: For raw paired discordance, the comparator is interpreted as a separate detection method rather than disease truth. No independent resolver unless stated; paired method comparison. F: Completeness of valid, same-specimen paired observations is not fully established for all reported analyses. Exact nail-specific paired binary cells unavailable or invalid/species states prevent reconstruction. A: Restricted applicability to unselected, first-line suspected onychomycosis: Narrative only. Yes-218 nail specimens; marginals only	Workbook v0.12 Evidence_Table row 80; Full_Text_Screening record OMD-2013-Winter; source locator: Full text Table 3 and Results. Existing full-text extraction only; source PDF not re-examined for this appraisal. Overlap: Likely overlapping 2007 cohort with Pankewitz et al. 2013 Pankewitz is outside the included-report matrix; no additional included paired row is assumed.
OMD-2006-Monod [88]	High / Unclear / Unclear / Unclear / High	S: Molecular validation used selected microscopy-positive nails from a much larger routine series. I: Prospective threshold selection and blinded interpretation are not established by the available assessment record. Molecular endpoint: Broad fungal 28S identification.	C: The extracted endpoint or its handling has unresolved reporting/target-scope qualifications. No independent resolver unless stated; paired method comparison. F: Completeness of valid, same-specimen paired observations is not fully established for all reported analyses. Exact nail-specific paired binary cells unavailable or invalid/species states prevent reconstruction. A: Restricted applicability to unselected, first-line suspected onychomycosis: Narrative only. Yes-111 selected microscopy-positive nails; 6,848-sample descriptive denominator	Workbook v0.12 Evidence_Table row 81; Full_Text_Screening record OMD-2006-Monod; source locator: Full text Tables 2-3. Existing full-text extraction only; source PDF not re-examined for this appraisal. Overlap: Precursor method/report related to Bontems et al. 2009; cohorts not identical
OMD-2013-Rothmund [89]	Unclear / Unclear / High / Unclear / Unclear	S: Recruitment sequence, consecutive enrollment and the complete exclusion process are not established by the available extraction. Yes-n=60. I: Prospective threshold selection and blinded interpretation are not established by the available assessment record. Molecular endpoint: Dermatophytes.	C: The composite contains PCR, culture and PAS and is circular for the performance of those component tests. F: Completeness of valid, same-specimen paired observations is not fully established for all reported analyses. Exact nail-specific paired binary cells unavailable or invalid/species states prevent reconstruction. A: Applicability depends on treatment, sampling, target or subgroup qualifications: Exact nail-specific paired binary cells unavailable or invalid/species states prevent reconstruction.	Workbook v0.12 Evidence_Table row 82; Full_Text_Screening record OMD-2013-Rothmund; source locator: Full text Table 1. Existing full-text extraction only; source PDF not re-examined for this appraisal. Overlap: No
OMD-2013-Miyajima [90]	High / Unclear / Unclear / Low / High	S: The clinical nail series was microscopy-positive; healthy controls were separate. I: Prospective threshold selection and blinded interpretation are not established by the available assessment record. The extraction uses the stated 1,500-copy threshold; prospective selection of that threshold and blinding are not established by the audit trail.	C: The extracted endpoint or its handling has unresolved reporting/target-scope qualifications. No independent resolver unless stated; paired method comparison. F: The extracted clinical/nail endpoint has an exact paired denominator; no additional selective verification or unresolved invalid-result loss is documented for those raw cells. Primary nail material divided or sampled for molecular and conventional testing; see source-specific notes. A: Restricted applicability to unselected, first-line suspected onychomycosis: Paired 2 × 2. Yes-32 clinical nails plus 39 healthy nail controls	Workbook v0.12 Evidence_Table row 83; Full_Text_Screening record OMD-2013-Miyajima; source locator: Full text Table 4. Existing full-text extraction only; source PDF not re-examined for this appraisal. Overlap: Assay-development group overlaps Uchida/Watanabe; no duplicate cohort established
OMD-2012-Luk [91]	Unclear / Unclear / Low / Low / Low	S: Recruitment sequence, consecutive enrollment and the complete exclusion process are not established by the available extraction. Yes-n=120. I: Prospective threshold selection and blinded interpretation are not established by the available assessment record. Molecular endpoint: Dermatophytes.	C: For raw paired discordance, the comparator is interpreted as a separate detection method rather than disease truth. No independent resolver unless stated; paired method comparison. F: The extracted clinical/nail endpoint has an exact paired denominator; no additional selective verification or unresolved invalid-result loss is documented for those raw cells. Primary nail material divided or sampled for molecular and conventional testing; see source-specific notes. A: Clinical nail comparison applicable within its stated organism target and setting; it does not establish all-pathogen accuracy or clinical benefit. Hong Kong	Workbook v0.12 Evidence_Table row 84; Full_Text_Screening record OMD-2012-Luk; source locator: Full text Table 2. Existing full-text extraction only; source PDF not re-examined for this appraisal. Overlap: No
OMD-2009-Ebihara [92]	High / Unclear / Unclear / Low / High	S: All specimens were microscopy-positive and no culture comparison was performed. I: Prospective threshold selection and blinded interpretation are not established by the available assessment record. Molecular endpoint: Dermatophytes and selected nondermatophytes.	C: The extracted endpoint or its handling has unresolved reporting/target-scope qualifications. No independent resolver unless stated; paired method comparison. F: The extracted clinical/nail endpoint has an exact paired denominator; no additional selective verification or unresolved invalid-result loss is documented for those raw cells. Primary nail material divided or sampled for molecular and conventional testing; see source-specific notes. A: Restricted applicability to unselected, first-line suspected onychomycosis: Paired 2 × 2. Yes-n=50	Workbook v0.12 Evidence_Table row 85; Full_Text_Screening record OMD-2009-Ebihara; source locator: Full text Table 3 and Results. Existing full-text extraction only; source PDF not re-examined for this appraisal. Overlap: No
OMD-2009-Bontems [93]	High / Unclear / Unclear / Unclear / High	S: The clinical series was microscopy-positive; it supports identification rather than an unselected diagnostic spectrum. I: Prospective threshold selection and blinded interpretation are not established by the available assessment record. Molecular endpoint: Broad fungal identification.	C: The extracted endpoint or its handling has unresolved reporting/target-scope qualifications. No independent resolver unless stated; paired method comparison. F: Completeness of valid, same-specimen paired observations is not fully established for all reported analyses. Exact nail-specific paired binary cells unavailable or invalid/species states prevent reconstruction. A: Restricted applicability to unselected, first-line suspected onychomycosis: Narrative only. Yes-n=410	Workbook v0.12 Evidence_Table row 86; Full_Text_Screening record OMD-2009-Bontems; source locator: Full text Table 2. Existing full-text extraction only; source PDF not re-examined for this appraisal. Overlap: Method lineage related to Monod et al. 2006; distinct larger cohort
OMD-2010-Litz [94]	Unclear / Unclear / Low / Low / Low	S: Recruitment sequence, consecutive enrollment and the complete exclusion process are not established by the available extraction. Yes-n=550. I: Prospective threshold selection and blinded interpretation are not established by the available assessment record. Molecular endpoint: Dermatophytes.	C: For raw paired discordance, the comparator is interpreted as a separate detection method rather than disease truth. No independent resolver unless stated; paired method comparison. F: The extracted clinical/nail endpoint has an exact paired denominator; no additional selective verification or unresolved invalid-result loss is documented for those raw cells. Primary nail material divided or sampled for molecular and conventional testing; see source-specific notes. A: Clinical nail comparison applicable within its stated organism target and setting; it does not establish all-pathogen accuracy or clinical benefit. United States; Dallas	Workbook v0.12 Evidence_Table row 87; Full_Text_Screening record OMD-2010-Litz; source locator: Full text Table 2. Existing full-text extraction only; source PDF not re-examined for this appraisal. Overlap: No
OMD-2018-Adel-Taha [95]	Unclear / Unclear / Low / Low / Low	S: Recruitment sequence, consecutive enrollment and the complete exclusion process are not established by the available extraction. Yes-n=50. I: Prospective threshold selection and blinded interpretation are not established by the available assessment record. Molecular endpoint: Pan-dermatophyte plus *T. rubrum*/*T. mentagrophytes*.	C: For raw paired discordance, the comparator is interpreted as a separate detection method rather than disease truth. No independent resolver unless stated; paired method comparison. F: The extracted clinical/nail endpoint has an exact paired denominator; no additional selective verification or unresolved invalid-result loss is documented for those raw cells. Primary nail material divided or sampled for molecular and conventional testing; see source-specific notes. A: Clinical nail comparison applicable within its stated organism target and setting; it does not establish all-pathogen accuracy or clinical benefit. Egypt	Workbook v0.12 Evidence_Table row 88; Full_Text_Screening record OMD-2018-Adel-Taha; source locator: Full text Tables 2-4 and Results. Existing full-text extraction only; source PDF not re-examined for this appraisal. Overlap: No
OMD-2011-Brasch [96]	Unclear / Unclear / Low / Low / Low	S: Recruitment sequence, consecutive enrollment and the complete exclusion process are not established by the available extraction. Yes-n=230 nails. I: Prospective threshold selection and blinded interpretation are not established by the available assessment record. Molecular endpoint: *T. rubrum*.	C: For raw paired discordance, the comparator is interpreted as a separate detection method rather than disease truth. No independent resolver unless stated; paired method comparison. F: The extracted clinical/nail endpoint has an exact paired denominator; no additional selective verification or unresolved invalid-result loss is documented for those raw cells. Primary nail material divided or sampled for molecular and conventional testing; see source-specific notes. A: Clinical nail comparison applicable within its stated organism target and setting; it does not establish all-pathogen accuracy or clinical benefit. Germany	Workbook v0.12 Evidence_Table row 89; Full_Text_Screening record OMD-2011-Brasch; source locator: Full text Results and Figure 3. Existing full-text extraction only; source PDF not re-examined for this appraisal. Overlap: No
OMD-1998-Baek [97]	High / Unclear / Unclear / Unclear / High	S: Feasibility work selected culture-proven infected nails and healthy nails; the clinical denominator is unavailable. I: Prospective threshold selection and blinded interpretation are not established by the available assessment record. Molecular endpoint: Broad fungal detection with class/species-pattern differentiation.	C: The extracted endpoint or its handling has unresolved reporting/target-scope qualifications. Culture-proven infected nails; no independent resolver. F: Completeness of valid, same-specimen paired observations is not fully established for all reported analyses. No clinical sample denominator, spectrum-selected specimens, and no paired diagnostic table. A: Restricted applicability to unselected, first-line suspected onychomycosis: Narrative only. Culture-proven fungus-infected nails and normal nails; clinical denominator not reported	Workbook v0.12 Evidence_Table row 90; Full_Text_Screening record OMD-1998-Baek; source locator: Full text Methods, Results and Table 1. Existing full-text extraction only; source PDF not re-examined for this appraisal. Overlap: No
OMD-2018-Alvarez-Mosquera [98]	Unclear / Unclear / Unclear / Unclear / High	S: Recruitment sequence, consecutive enrollment and the complete exclusion process are not established by the available extraction. 626 unselected clinical specimens, including 440 toenails and 83 fingernails. I: Prospective threshold selection and blinded interpretation are not established by the available assessment record. Molecular endpoint: Broad fungal detection and sequencing.	C: The extracted endpoint or its handling has unresolved reporting/target-scope qualifications. No independent resolver; three-method comparison. F: Completeness of valid, same-specimen paired observations is not fully established for all reported analyses. Mixed specimen types in the cross-table; incomplete sequencing of PCR-positive specimens; complete nail-only 2×2 cannot be reconstructed. A: Restricted applicability to unselected, first-line suspected onychomycosis: Narrative only. 626 unselected clinical specimens, including 440 toenails and 83 fingernails	Workbook v0.12 Evidence_Table row 91; Full_Text_Screening record OMD-2018-Alvarez-Mosquera; source locator: Full text Tables 1-3 and Results. Existing full-text extraction only; source PDF not re-examined for this appraisal. Overlap: No
OMD-2025-Wang-RPA [99]	Low / Unclear / Low / Low / Low	S: The extraction explicitly describes continuously collected clinically suspected nail samples. I: Prospective threshold selection and blinded interpretation are not established by the available assessment record. Molecular endpoint: Dermatophytes; reflex *T. rubrum* identification.	C: For raw paired discordance, the comparator is interpreted as a separate detection method rather than disease truth. Latent class analysis of microscopy, culture and RPA-LFD; pairwise cells retained. F: The extracted clinical/nail endpoint has an exact paired denominator; no additional selective verification or unresolved invalid-result loss is documented for those raw cells. Each sample split: two-thirds for microscopy/culture and one-third stored for molecular testing. A: Clinical nail comparison applicable within its stated organism target and setting; it does not establish all-pathogen accuracy or clinical benefit. China; Beijing	Workbook v0.12 Evidence_Table row 92; Full_Text_Screening record OMD-2025-Wang-RPA; source locator: Full text Tables 3-4. Existing full-text extraction only; source PDF not re-examined for this appraisal. Overlap: No
OMD-2012-Dhib [100]	Unclear / Unclear / Low / Unclear / Unclear	S: Recruitment sequence, consecutive enrollment and the complete exclusion process are not established by the available extraction. 201 nails from suspected onychomycosis patients plus 80 healthy nail controls. I: Prospective threshold selection and blinded interpretation are not established by the available assessment record. Molecular endpoint: Dermatophytes.	C: For raw paired discordance, the comparator is interpreted as a separate detection method rather than disease truth. No independent resolver; clinical cohort plus healthy controls. F: Completeness of valid, same-specimen paired observations is not fully established for all reported analyses. No independent reference; suspected cohort plus healthy controls; contaminated cultures require separate handling. A: Applicability depends on treatment, sampling, target or subgroup qualifications: No independent reference; suspected cohort plus healthy controls; contaminated cultures require separate handling.	Workbook v0.12 Evidence_Table row 93; Full_Text_Screening record OMD-2012-Dhib; source locator: Full text Tables 2-3. Existing full-text extraction only; source PDF not re-examined for this appraisal. Overlap: Probable overlap: same Sousse group, 201 suspected nails and 80 controls, identical conventional marginals (microscopy 151, culture 132) in Dhib2012/2014; recruitment linkage not confirmed. Conservatively test one-report retention.
OMD-2019-Koo [101]	Unclear / Unclear / Low / Low / Low	S: Recruitment sequence, consecutive enrollment and the complete exclusion process are not established by the available extraction. 201 patients with suspected onychomycosis contributing 204 nail specimens. I: Prospective threshold selection and blinded interpretation are not established by the available assessment record. Molecular endpoint: Pan-dermatophytes, *Fusarium* spp., *T. rubrum* and *Microsporum* spp.	C: For raw paired discordance, the comparator is interpreted as a separate detection method rather than disease truth. Culture; discordances adjudicated with repeat qPCR and ITS2 sequencing. F: The extracted clinical/nail endpoint has an exact paired denominator; no additional selective verification or unresolved invalid-result loss is documented for those raw cells. Nail material divided for microscopy, culture and qPCR. A: Clinical nail comparison applicable within its stated organism target and setting; it does not establish all-pathogen accuracy or clinical benefit. Singapore	Workbook v0.12 Evidence_Table row 94; Full_Text_Screening record OMD-2019-Koo; source locator: Full text Table 3. Existing full-text extraction only; source PDF not re-examined for this appraisal. Overlap: No
OMD-2020-Ross [102]	Unclear / Unclear / Low / Low / Low	S: Recruitment sequence, consecutive enrollment and the complete exclusion process are not established by the available extraction. 156 prospectively tested nail specimens in the dermatophyte comparison. I: Prospective threshold selection and blinded interpretation are not established by the available assessment record. Molecular endpoint: Dermatophytes plus selected yeasts and molds; discordance rows use dermatophyte endpoint.	C: For raw paired discordance, the comparator is interpreted as a separate detection method rather than disease truth. No independent resolver; microscopy/culture combinations. F: The extracted clinical/nail endpoint has an exact paired denominator; no additional selective verification or unresolved invalid-result loss is documented for those raw cells. Clinical specimens tested in parallel by microscopy, culture and commercial tandem PCR. A: Clinical nail comparison applicable within its stated organism target and setting; it does not establish all-pathogen accuracy or clinical benefit. Australia; South Australia	Workbook v0.12 Evidence_Table row 95; Full_Text_Screening record OMD-2020-Ross; source locator: Full text Tables 1-2. Existing full-text extraction only; source PDF not re-examined for this appraisal. Overlap: No
OMD-2021-Lin [103]	Unclear / Unclear / Low / Unclear / Low	S: Recruitment sequence, consecutive enrollment and the complete exclusion process are not established by the available extraction. Part 3: 128 prospective nail samples; Part 2: 47 culture-positive nail samples. I: Prospective threshold selection and blinded interpretation are not established by the available assessment record. Molecular endpoint: Dermatophytes.	C: For raw paired discordance, the comparator is interpreted as a separate detection method rather than disease truth. No independent resolver; sequencing of selected species discrepancies. F: Completeness of valid, same-specimen paired observations is not fully established for all reported analyses. Nail composite union reconstructed from Figure 1 and exact totals; selected culture-positive validation is a separate cohort; manufacturer supplied kits. A: Clinical nail comparison applicable within its stated organism target and setting; it does not establish all-pathogen accuracy or clinical benefit. Australia; Victoria	Workbook v0.12 Evidence_Table row 96; Full_Text_Screening record OMD-2021-Lin; source locator: Full text Tables 2-3, Figure 1. Existing full-text extraction only; source PDF not re-examined for this appraisal. Overlap: No established overlap with Ross; separate laboratory and cohort
OMD-2021-Cuchi-Burgos [104]	High / Unclear / Unclear / High / High	S: Samples were deliberately selected at a 3:1 KOH-positive:negative ratio. I: Prospective threshold selection and blinded interpretation are not established by the available assessment record. Molecular endpoint: Dermatophytes and Candida albicans.	C: The extracted endpoint or its handling has unresolved reporting/target-scope qualifications. No independent resolver; workflow comparison. F: Ten invalid molecular results were excluded from 152 selected samples; analyses describe 142 valid pairs rather than all submitted specimens. A: Restricted applicability to unselected, first-line suspected onychomycosis: Paired 2 × 2. 152 clinical nail samples selected at a 3:1 KOH-positive:negative ratio	Workbook v0.12 Evidence_Table row 97; Full_Text_Screening record OMD-2021-Cuchi-Burgos; source locator: Full text Table 1. Existing full-text extraction only; source PDF not re-examined for this appraisal. Overlap: No
OMD-2026-Sharma [28]	Unclear / Unclear / Unclear / Low / Unclear	S: Recruitment sequence, consecutive enrollment and the complete exclusion process are not established by the available extraction. 65 clinically suspected onychomycosis patients. I: Prospective threshold selection and blinded interpretation are not established by the available assessment record. Molecular endpoint: Common dermatophytes with species identification.	C: The extracted endpoint or its handling has unresolved reporting/target-scope qualifications. No independent resolver. F: The extracted clinical/nail endpoint has an exact paired denominator; no additional selective verification or unresolved invalid-result loss is documented for those raw cells. Each nail scraping divided for KOH, culture and PCR. A: Applicability depends on treatment, sampling, target or subgroup qualifications: Small single-center cohort, very high KOH positivity, no independent reference, and restricted commercial target panel. Treatment exposure: Prior antifungal use discussed but not used as an exclusion criterion	Workbook v0.12 Evidence_Table row 98; Full_Text_Screening record OMD-2026-Sharma; source locator: Full text Tables 1-2. Existing full-text extraction only; source PDF not re-examined for this appraisal. Overlap: No
OMD-2004-Menotti [9]	Unclear / Unclear / Low / High / Unclear	S: Recruitment sequence, consecutive enrollment and the complete exclusion process are not established by the available extraction. Series 1: 48 patients with optimally collected nail samples; series 2: 88 patients contributing a non-optimal clipping for PCR and an optimally collected specimen from the same nail for conventional mycology. I: Prospective threshold selection and blinded interpretation are not established by the available assessment record. Molecular endpoint: Dermatophytes versus *Scytalidium*; does not detect Candida or other molds and does not identify individual dermatophyte species.	C: For raw paired discordance, the comparator is interpreted as a separate detection method rather than disease truth. Positive conventional result defined as direct microscopy and/or culture on the optimally collected sample. F: Series 2 pairs a non-optimal PCR clipping with an optimally collected conventional specimen from the same nail; sample quality differs systematically. A: Applicability depends on treatment, sampling, target or subgroup qualifications: Comparator is a microscopy-or-culture composite rather than a single reference; the second series changes sampling quality between index and comparator; narrow taxonomic scope; no independent adjudication.	Workbook v0.12 Evidence_Table row 99; Full_Text_Screening record OMD-2004-Menotti; source locator: Full text Table 1 and Results. Existing full-text extraction only; source PDF not re-examined for this appraisal. Overlap: No
OMD-2007-Gupta-DoublePCR [10]	High / Unclear / Low / Low / High	S: Eligibility required a previous *T. rubrum*-positive culture, restricting the spectrum before contemporaneous paired testing. I: Prospective threshold selection and blinded interpretation are not established by the available assessment record. Analytical cross-reactivity with *T. violaceum* limits species-level attribution; the review does not equate species attribution with generic fungal presence.	C: For raw paired discordance, the comparator is interpreted as a separate detection method rather than disease truth. No independent resolver; prior culture positivity was an entry criterion. F: The extracted clinical/nail endpoint has an exact paired denominator; no additional selective verification or unresolved invalid-result loss is documented for those raw cells. Each nail sample was divided into aliquots for KOH microscopy, contemporaneous culture, and direct DNA extraction. A: Restricted applicability to unselected, first-line suspected onychomycosis: Paired 2 × 2. 62 patients with toenail onychomycosis and a prior *T. rubrum*-positive culture. Treatment exposure: Mixed; at least 15 culture-negative/actin-PCR-positive samples were obtained during antifungal treatment	Workbook v0.12 Evidence_Table row 100; Full_Text_Screening record OMD-2007-Gupta-DoublePCR; source locator: Full text Table 2 and Results. Existing full-text extraction only; source PDF not re-examined for this appraisal. Overlap: No
OMD-2024-Felek [8]	High / Unclear / Low / High / High	S: Only 37/46 suspected cases with positive culture and/or EUROArray enter the reported cohort. Conditioning on either test restricts the spectrum and negative-cell interpretation (p. 1132). I: Manufacturer protocol and four assay controls are described; interpretation blinded to culture and prospective study-specific thresholds are not established (p. 1132).	C: Culture is a separately acquired detection endpoint, not disease truth. Panel omissions are tracked under applicability; the entry criterion is assessed under selection. F: Patient-linked pairs are reconstructable for 37 selected cases, but nine of 46 evaluated cases are not individually displayed; KOH available for 18 only; conflicting culture summaries require use of Table 2 and Results (pp. 1132-1135). A: Positive-test-enriched sample; 21/37 prior antifungal exposure; no treatment timing or stratified cells; panel excludes Aspergillus/Alternaria and NDM not confirmed on repeat sampling (pp. 1132-1135).	New complete full-text assessment and Table 2 visual check on September 21, 2026. Newly retrieved full text read in full; patient-level Table 2 transcribed and reconstructed by organism class. Overlap: No overlap with other included reports identified; D171-D174 share the same 37 patients.

Appendix 4

Comparison Selection and Analytic Amendments

Table 13 preserves every original designation and documents the revised rule for all 170 historical rows, four new Felek R rows, and D089V. H40 indicates membership in the historical 40-row culture set; H37 indicates the one-per-report reduction of that set; B50 indicates selection for the broader 50-report analysis. Small sample size, zero cells, and exact reconstruction alone do not exclude a row. The largest eligible N is preferred, followed by target breadth and frozen row ID. Standard thresholds and same-material sampling take precedence over alternate gray-zone or anatomically mismatched contrasts. The source register and full selection script accompany the revision package.

**Table 13:** Complete historical designations and revised selection audit. KOH: Potassium hydroxide; RPA: Recombinase polymerase amplification; LAMP: Loop-mediated isothermal amplification; NDM: Non-dermatophyte mold.

Row / report	Historical designation and reason	H40 / H37 / B50	Broader-rule decision and qualification
D001 OMD-2006-Walberg	Main candidate Complete paired; culture comparator	Yes / Yes / Yes	Selected: eligible clinical nail detection-culture pair; largest analyzable N, then broadest target class, then frozen row-ID tie-break.
D002 OMD-2024-Gupta-US	Main candidate Histopathology comparator; overlap check	No / No / No	Outside culture synthesis; retained in the comparator-specific paired map.
D003 OMD-2023-Marin-Maldonado	Separate Verification-selected subset	No / No / No	Outside culture synthesis; retained in the comparator-specific paired map.
D004 OMD-2023-Marin-Maldonado	Separate Verification-selected subset; zero cell	No / No / No	Outside culture synthesis; retained in the comparator-specific paired map.
D005 OMD-2026-Dey	Sensitivity High classification-bias concern	No / No / No	Target-class mismatch: universal fungal PCR versus dermatophyte/*C. albicans* culture; NDM excluded from comparator.
D006 OMD-2026-Villanueva-Reyes	Separate Case/control spectrum and composite incorporation	No / No / No	Outside culture synthesis; retained in the comparator-specific paired map.
D007 OMD-2026-Villanueva-Reyes	Separate Case-only spectrum	No / No / No	Laboratory/composite-confirmed case-only spectrum.
D008 OMD-2018-Bao	Main candidate Complete paired; culture comparator	Yes / Yes / Yes	Selected: eligible clinical nail detection-culture pair; largest analyzable N, then broadest target class, then frozen row-ID tie-break.
D009 OMD-2019-Gustafson	Main candidate Complete paired; binary detection	Yes / No / No	Eligible alternative from same report; D011 retained by N, target breadth, frozen-ID hierarchy.
D010 OMD-2019-Gustafson	Main candidate Histopathology comparator	No / No / No	Outside culture synthesis; retained in the comparator-specific paired map.
D011 OMD-2019-Gustafson	Main candidate Culture comparator; choose nonoverlapping cohort	Yes / Yes / Yes	Selected: eligible clinical nail detection-culture pair; largest analyzable N, then broadest target class, then frozen row-ID tie-break.
D012 OMD-2007-Garg	Separate Mixed treated/untreated; zero cell	No / No / No	Mixture includes a deliberately selected treated-nonresponder stratum; use the separately available untreated clinical stratum D015.
D013 OMD-2007-Garg	Separate Mixed treated/untreated	No / No / No	Outside culture synthesis; retained in the comparator-specific paired map.
D014 OMD-2007-Garg	Separate Treated stratum; multiple zero cells	No / No / No	Treated-nonresponder stratum.
D015 OMD-2007-Garg	Separate Untreated stratum; no discordance	No / No / Yes	Selected: eligible clinical nail detection-culture pair; largest analyzable N, then broadest target class, then frozen row-ID tie-break. Untreated stratum retained; treated-nonresponder strata excluded.
D016 OMD-2007-Brillowska-Dabrowska	Main candidate Complete paired; culture comparator	Yes / Yes / Yes	Selected: eligible clinical nail detection-culture pair; largest analyzable N, then broadest target class, then frozen row-ID tie-break.
D017 OMD-2006-Kardjeva	Main candidate Direct nail cohort; zero cell	Yes / Yes / Yes	Selected: eligible clinical nail detection-culture pair; largest analyzable N, then broadest target class, then frozen row-ID tie-break.
D018 OMD-2006-Kardjeva	Main candidate Direct nail cohort; microscopy comparator	No / No / No	Outside culture synthesis; retained in the comparator-specific paired map.
D019 OMD-2026-Guegan	Main candidate Prospective multicenter nail subgroup	Yes / Yes / Yes	Selected: eligible clinical nail detection-culture pair; largest analyzable N, then broadest target class, then frozen row-ID tie-break.
D020 OMD-2026-Guegan	Main candidate Composite comparator; analyze separately	No / No / No	Outside culture synthesis; retained in the comparator-specific paired map.
D021 OMD-2022-Pospischil	Separate Reflex/conditional cohort	No / No / No	Reflex/conditional PCR testing after inconclusive conventional workup.
D022 OMD-2012-Verrier	Separate Microscopy-positive method-development cohort	No / No / No	Microscopy-positive selection.
D023 OMD-2022-Bieber	Sensitivity Small composite-reference study	No / No / Yes	Selected: eligible clinical nail detection-culture pair; largest analyzable N, then broadest target class, then frozen row-ID tie-break.
D024 OMD-2022-Bieber	Sensitivity Small study; microscopy comparator	No / No / No	Outside culture synthesis; retained in the comparator-specific paired map.
D025 OMD-2022-Bieber	Sensitivity Composite incorporates conventional methods	No / No / No	Outside culture synthesis; retained in the comparator-specific paired map.
D026 OMD-2024-Katsiaunis	Main candidate Category-specific; culture comparator	Yes / Yes / Yes	Selected: eligible clinical nail detection-culture pair; largest analyzable N, then broadest target class, then frozen row-ID tie-break.
D027 OMD-2024-Katsiaunis	Main candidate Category-specific; do not combine with dermatophyte row	Yes / No / No	Eligible alternative from same report; D026 retained by N, target breadth, frozen-ID hierarchy.
D028 OMD-2024-Harel	Main candidate Nail-specific culture comparator	Yes / Yes / Yes	Selected: eligible clinical nail detection-culture pair; largest analyzable N, then broadest target class, then frozen row-ID tie-break.
D029 OMD-2024-Harel	Separate Composite incorporation risk; zero cell	No / No / No	Outside culture synthesis; retained in the comparator-specific paired map.
D030 OMD-2007-Savin	Main candidate Prospective multicenter regional samples	Yes / Yes / Yes	Selected: eligible clinical nail detection-culture pair; largest analyzable N, then broadest target class, then frozen row-ID tie-break.
D031 OMD-2007-Savin	Sensitivity Threshold sensitivity analysis	No / No / No	Alternative gray-zone-positive threshold; retain author primary positive-only threshold.
D032 OMD-2007-Savin	Separate Microscopy comparator has broader target	No / No / No	Outside culture synthesis; retained in the comparator-specific paired map.
D033 OMD-2007-Savin	Separate Linked distal/proximal samples, not same aliquot	No / No / No	Different anatomically linked distal/proximal samples across index and comparator.
D034 OMD-2007-Savin	Sensitivity Threshold and sampling-location sensitivity	No / No / No	Different anatomically linked distal/proximal samples and alternative threshold.
D035 OMD-2007-Savin	Separate Linked distal/proximal samples; microscopy target mismatch	No / No / No	Outside culture synthesis; retained in the comparator-specific paired map.
D036 OMD-2007-Savin	Main candidate Routine regional sample stratum; zero cell	Yes / No / No	Eligible alternative from same report; D030 retained by N, target breadth, frozen-ID hierarchy.
D037 OMD-2007-Savin	Sensitivity Threshold sensitivity analysis; zero cell	No / No / No	Alternative gray-zone-positive threshold; retain author primary positive-only threshold.
D038 OMD-2007-Savin	Separate Microscopy comparator has broader target	No / No / No	Outside culture synthesis; retained in the comparator-specific paired map.
D039 OMD-2024-Navarro-Perez	Main candidate Complete paired culture comparison	Yes / Yes / Yes	Selected: eligible clinical nail detection-culture pair; largest analyzable N, then broadest target class, then frozen row-ID tie-break.
D040 OMD-2024-Evrard	Main candidate Culture comparator	Yes / Yes / Yes	Selected: eligible clinical nail detection-culture pair; largest analyzable N, then broadest target class, then frozen row-ID tie-break.
D041 OMD-2024-Evrard	Separate Composite incorporation risk; zero cell	No / No / No	Outside culture synthesis; retained in the comparator-specific paired map.
D042 OMD-2024-Evrard	Separate Diagnostic strategy, not single index test; zero cell	No / No / No	Outside culture synthesis; retained in the comparator-specific paired map.
D043 OMD-2017-Iwanaga	Separate Treatment-monitoring baseline; zero cells	No / No / No	KOH-confirmed treatment-monitoring cohort, including its selected baseline.
D044 OMD-2017-Iwanaga	Separate Treated repeated-measure stratum; multiple zero cells	No / No / No	Repeated treatment-monitoring time point.
D045 OMD-2017-Iwanaga	Separate Treated repeated-measure stratum; multiple zero cells	No / No / No	Repeated treatment-monitoring time point.
D046 OMD-2017-Iwanaga	Separate Spectrum selection; all cells concordant-positive	No / No / No	Outside culture synthesis; retained in the comparator-specific paired map.
D047 OMD-2017-Iwanaga	Separate Treated repeated-measure stratum; zero cells	No / No / No	Outside culture synthesis; retained in the comparator-specific paired map.
D048 OMD-2017-Iwanaga	Separate Treated repeated-measure stratum; zero cells	No / No / No	Outside culture synthesis; retained in the comparator-specific paired map.
D049 OMD-2018-Lubis	Sensitivity Small complete-paired study	No / No / Yes	Selected: eligible clinical nail detection-culture pair; largest analyzable N, then broadest target class, then frozen row-ID tie-break.
D050 OMD-2014-Han	Sensitivity Small study; raw culture comparison; zero cell	No / No / Yes	Selected: eligible clinical nail detection-culture pair; largest analyzable N, then broadest target class, then frozen row-ID tie-break.
D051 OMD-2014-Han	Separate Selective adjudication and incorporation risk; zero cell	No / No / No	Outside culture synthesis; retained in the comparator-specific paired map.
D052 OMD-2026-Wu	Separate RPA grouping decision open; zero cell	No / No / Yes	Selected: eligible clinical nail detection-culture pair; largest analyzable N, then broadest target class, then frozen row-ID tie-break.
D053 OMD-2026-Wu	Separate RPA grouping decision open; microscopy comparator	No / No / No	Outside culture synthesis; retained in the comparator-specific paired map.
D054 OMD-2021-Kabtani	Separate Very small mixed-site nail subgroup; zero cell	No / No / No	Outside culture synthesis; retained in the comparator-specific paired map.
D055 OMD-2021-Kabtani	Separate Very small subgroup; commercial involvement; zero cell	No / No / No	Outside culture synthesis; retained in the comparator-specific paired map.
D056 OMD-2010-Sato	Separate Spectrum-selected method-development cohort; zero cell	No / No / No	Microscopy-positive selection.
D057 OMD-2010-Sato	Separate Identification endpoint; spectrum selection; zero cell	No / No / No	Microscopy-positive selection and successful identification rather than a detection endpoint.
D058 OMD-2023-Gupta-NDM	Main candidate Histopathology comparator; zero cell	No / No / No	Outside culture synthesis; retained in the comparator-specific paired map.
D059 OMD-2023-Gupta-NDM	Main candidate NDM-specific culture comparison	Yes / Yes / Yes	Selected: eligible clinical nail detection-culture pair; largest analyzable N, then broadest target class, then frozen row-ID tie-break.
D060 OMD-2020-Pakshir	Main candidate Candida-specific culture comparison	Yes / Yes / Yes	Selected: eligible clinical nail detection-culture pair; largest analyzable N, then broadest target class, then frozen row-ID tie-break.
D061 OMD-2022-Trovato	Separate Mixed treated/untreated; target mismatch	No / No / No	Outside culture synthesis; retained in the comparator-specific paired map.
D062 OMD-2022-Trovato	Separate Mixed treated/untreated culture comparison	No / No / Yes	Selected: eligible clinical nail detection-culture pair; largest analyzable N, then broadest target class, then frozen row-ID tie-break.
D063 OMD-2022-Trovato	Separate Mixed treated/untreated composite; zero cell	No / No / No	Outside culture synthesis; retained in the comparator-specific paired map.
D064 OMD-2022-Trovato	Separate Untreated stratum; zero cell	No / No / No	Outside culture synthesis; retained in the comparator-specific paired map.
D065 OMD-2022-Trovato	Separate Treated stratum; zero cell	No / No / No	Outside culture synthesis; retained in the comparator-specific paired map.
D066 OMD-2017-Vrioni	Main candidate Prospective complete paired; prior treatment permitted	Yes / Yes / Yes	Selected: eligible clinical nail detection-culture pair; largest analyzable N, then broadest target class, then frozen row-ID tie-break.
D067 OMD-2017-Vrioni	Separate Composite comparator and microscopy target mismatch	No / No / No	Outside culture synthesis; retained in the comparator-specific paired map.
D068 OMD-2026-Gellrich	Sensitivity Nail-specific reconstruction; denominator discrepancy	No / No / Yes	Selected: eligible clinical nail detection-culture pair; largest analyzable N, then broadest target class, then frozen row-ID tie-break. Nail denominator 51 in one table versus 52 elsewhere.
D069 OMD-2023-Guschin	Sensitivity Small nail subgroup; culture comparator	No / No / Yes	Selected: eligible clinical nail detection-culture pair; largest analyzable N, then broadest target class, then frozen row-ID tie-break.
D070 OMD-2023-Guschin	Separate Small subgroup; microscopy target broader than PCR	No / No / No	Outside culture synthesis; retained in the comparator-specific paired map.
D071 OMD-2022-Makled	Main candidate Complete paired culture comparison; sequencing subset	Yes / Yes / Yes	Selected: eligible clinical nail detection-culture pair; largest analyzable N, then broadest target class, then frozen row-ID tie-break.
D072 OMD-2019-Camacaro	Sensitivity Small reconstructed table; different nail portions	No / No / Yes	Selected: eligible clinical nail detection-culture pair; largest analyzable N, then broadest target class, then frozen row-ID tie-break. Reconstructed cells imply 100% sensitivity; reported 97% is incompatible.
D073 OMD-2013-Chandran	Sensitivity Target-matched culture; selected spectrum	No / No / No	Microscopy-positive selection.
D074 OMD-2013-Chandran	Separate Any-culture target mismatch	No / No / No	Microscopy-positive selection and dermatophyte-versus-any-fungal target-class mismatch.
D075 OMD-2013-Chandran	Separate Spectrum-selected microscopy comparator; zero cells	No / No / No	Outside culture synthesis; retained in the comparator-specific paired map.
D076 OMD-2017-Hafirassou	Sensitivity Target-matched culture	No / No / No	Eligible alternative from same report; D078 retained by N, target breadth, frozen-ID hierarchy.
D077 OMD-2017-Hafirassou	Separate Microscopy target broader than dermatophyte PCR	No / No / No	Outside culture synthesis; retained in the comparator-specific paired map.
D078 OMD-2017-Hafirassou	Sensitivity Raw band endpoint; broader than identification	No / No / Yes	Selected: eligible clinical nail detection-culture pair; largest analyzable N, then broadest target class, then frozen row-ID tie-break. Raw broad fungal detection precedes narrow dermatophyte detection; identification endpoint excluded.
D079 OMD-2017-Hafirassou	Sensitivity Identification endpoint	No / No / No	Successful sequence identification endpoint, not raw molecular detection; detection rows D076/D078 available.
D080 OMD-2022-Gupta-Therapy	Separate Treatment-study baseline; spectrum selected	No / No / No	Culture-positive treatment-study baseline.
D081 OMD-2022-Gupta-Therapy	Separate Treated repeated-measure stratum	No / No / No	Repeated treatment-monitoring time point.
D082 OMD-2022-Gupta-Therapy	Separate Treated repeated-measure/composite stratum	No / No / No	Outside culture synthesis; retained in the comparator-specific paired map.
D083 OMD-2014-Dhib	Sensitivity Complete paired culture comparison; zero cell	No / No / Yes	Selected: eligible clinical nail detection-culture pair; largest analyzable N, then broadest target class, then frozen row-ID tie-break.
D084 OMD-2014-Dhib	Separate Microscopy comparator broader; zero cell	No / No / No	Outside culture synthesis; retained in the comparator-specific paired map.
D085 OMD-2016-Gong	Sensitivity Case/control spectrum; target-matched culture	No / No / No	Suspected cases and asymptomatic controls combined in the paired denominator.
D086 OMD-2016-Gong	Separate Case/control spectrum and target mismatch	No / No / No	Outside culture synthesis; retained in the comparator-specific paired map.
D087 OMD-2020-Caldwell	Main candidate Direct histopathology comparison	No / No / No	Outside culture synthesis; retained in the comparator-specific paired map.
D088 OMD-2019-Hayette	Main candidate Histology comparator	No / No / No	Outside culture synthesis; retained in the comparator-specific paired map.
D089 OMD-2019-Hayette	Main candidate Target-matched culture	Yes / Yes / No	Original table includes eight inhibited PCR results as index-negative. Use explicitly derived valid-only D089V for revision sensitivity; preserve D089 as historical audit.
D090 OMD-2019-Hayette	Separate Composite reference; comparator definition differs	No / No / No	Outside culture synthesis; retained in the comparator-specific paired map.
D091 OMD-2011-Berk	Separate Single-species index versus broad microscopy	No / No / No	Outside culture synthesis; retained in the comparator-specific paired map.
D092 OMD-2011-Berk	Sensitivity Target-matched culture; zero cell	No / No / Yes	Selected: eligible clinical nail detection-culture pair; largest analyzable N, then broadest target class, then frozen row-ID tie-break.
D093 OMD-2023-Debuysschere	Main candidate Prospective complete paired; molecular resolver	Yes / Yes / Yes	Selected: eligible clinical nail detection-culture pair; largest analyzable N, then broadest target class, then frozen row-ID tie-break.
D094 OMD-2010-Brillowska-Routine	Main candidate Routine complete paired culture comparison	Yes / Yes / Yes	Selected: eligible clinical nail detection-culture pair; largest analyzable N, then broadest target class, then frozen row-ID tie-break.
D095 OMD-2010-Brillowska-Routine	Separate Microscopy target broader than PCR	No / No / No	Outside culture synthesis; retained in the comparator-specific paired map.
D096 OMD-2010-Brillowska-Routine	Separate Composite comparator	No / No / No	Outside culture synthesis; retained in the comparator-specific paired map.
D097 OMD-2010-Kondori	Main candidate Complete paired target-culture comparison	Yes / Yes / Yes	Selected: eligible clinical nail detection-culture pair; largest analyzable N, then broadest target class, then frozen row-ID tie-break.
D098 OMD-2010-Kondori	Separate Microscopy target broader than PCR	No / No / No	Outside culture synthesis; retained in the comparator-specific paired map.
D099 OMD-2014-Dubljanin	Main candidate Culture comparison; one Aspergillus culture is off-target	Yes / Yes / No	Target-class mismatch: dermatophyte PCR versus any fungal culture, including an Aspergillus isolate.
D100 OMD-2014-Dubljanin	Separate Microscopy target broader than PCR	No / No / No	Outside culture synthesis; retained in the comparator-specific paired map.
D101 OMD-2014-Dubljanin	Separate Composite comparator; table/text overlap discrepancy	No / No / No	Outside culture synthesis; retained in the comparator-specific paired map.
D102 OMD-2026-Caza	Main candidate Specimen-level culture comparison	Yes / Yes / Yes	Selected: eligible clinical nail detection-culture pair; largest analyzable N, then broadest target class, then frozen row-ID tie-break. Specimen-level table reconstructed from several margins and agreement after resolving correlated target counts.
D103 OMD-2026-Caza	Separate Specimen-level composite comparison; four specimens not tested by KOH retained according to published composite classification	No / No / No	Outside culture synthesis; retained in the comparator-specific paired map.
D104 OMD-2026-Caza	Support only Six correlated target determinations per nail; not an independent sample-level analysis	No / No / No	Six correlated target determinations per nail; unit is not an independent nail pair.
D105 OMD-2026-Caza	Support only Six correlated target determinations per nail; not an independent sample-level analysis	No / No / No	Outside culture synthesis; retained in the comparator-specific paired map.
D106 OMD-2026-Caza	Support only Selective index-incorporating molecular resolver and correlated target determinations	No / No / No	Outside culture synthesis; retained in the comparator-specific paired map.
D107 OMD-2025-Tirard-Collet	Support only Article states 29 nails but Table 1 accounts for 27; two invalid and two unaccounted nails; any-culture target mismatch	No / No / No	Unresolved nail denominator/missingness (29 stated, 27 tabulated, 25 analyzable) and target-class mismatch.
D108 OMD-2025-Kharibam	Separate KOH-positive enrichment and narrow two-species panel; off-panel dermatophytes drive reverse discordance	No / No / No	KOH-positive enrichment and narrow two-species panel versus all cultured dermatophytes.
D109 OMD-2020-Gill	Sensitivity Strong spectrum enrichment and zero PCR-/culture- cell	No / No / No	Strong fungal-spectrum enrichment and dermatophyte-versus-any-fungal target-class mismatch.
D110 OMD-2008-Gupta-Treated	Separate Treated cohort and species-specific versus structural target mismatch	No / No / No	Outside culture synthesis; retained in the comparator-specific paired map.
D111 OMD-2008-Gupta-Treated	Separate Treated cohort; near-complete culture suppression	No / No / No	Patients specifically recruited during antifungal therapy.
D112 OMD-2014-Iglesias-Sanchez	Main candidate Complete paired data; aliquot heterogeneity likely	Yes / Yes / Yes	Selected: eligible clinical nail detection-culture pair; largest analyzable N, then broadest target class, then frozen row-ID tie-break.
D113 OMD-2015-Spiliopoulou	Main candidate Complete paired target-matched culture comparison	Yes / Yes / Yes	Selected: eligible clinical nail detection-culture pair; largest analyzable N, then broadest target class, then frozen row-ID tie-break.
D114 OMD-2015-Spiliopoulou	Separate Structural comparator and target mismatch	No / No / No	Outside culture synthesis; retained in the comparator-specific paired map.
D115 OMD-2023-Navarro-Perez-Culture	Separate Comparator includes yeasts and molds outside PCR target breadth	No / No / No	Target-class mismatch: dermatophyte PCR versus any fungal culture, including yeast/mold results.
D116 OMD-2020-Mohtady	Separate Small cohort, zero reverse-discordant cell and internal marginal-label inconsistency	No / No / Yes	Selected: eligible clinical nail detection-culture pair; largest analyzable N, then broadest target class, then frozen row-ID tie-break. Coherent cross-table conflicts with marginal wording.
D117 OMD-2016-Emam	Separate Cells reconstructed; culture-reference assumption	No / No / Yes	Selected: eligible clinical nail detection-culture pair; largest analyzable N, then broadest target class, then frozen row-ID tie-break. Both assay variants tie on N/target; real-time PCR row is first in frozen register.
D118 OMD-2016-Emam	Separate Cells reconstructed; culture-reference assumption	No / No / No	Eligible alternative from same report; D117 retained by N, target breadth, frozen-ID hierarchy.
D119 OMD-2011-Li	Main candidate Study composite is circular; direct paired comparison retained	Yes / Yes / Yes	Selected: eligible clinical nail detection-culture pair; largest analyzable N, then broadest target class, then frozen row-ID tie-break.
D120 OMD-2011-Li	Separate Structural comparator and target mismatch	No / No / No	Outside culture synthesis; retained in the comparator-specific paired map.
D121 OMD-2004-Arca	Main candidate Exact conditional counts and marginals	Yes / Yes / Yes	Selected: eligible clinical nail detection-culture pair; largest analyzable N, then broadest target class, then frozen row-ID tie-break.
D122 OMD-2004-Arca	Separate Structural comparator	No / No / No	Outside culture synthesis; retained in the comparator-specific paired map.
D123 OMD-2022-Trave	Main candidate Complete eight-pattern table	Yes / Yes / Yes	Selected: eligible clinical nail detection-culture pair; largest analyzable N, then broadest target class, then frozen row-ID tie-break.
D124 OMD-2022-Trave	Separate Structural comparator and breadth mismatch	No / No / No	Outside culture synthesis; retained in the comparator-specific paired map.
D125 OMD-2019-Watanabe-LAMP	Separate Verification-selected microscopy-positive spectrum	No / No / No	Microscopy-positive selection.
D126 OMD-2019-Watanabe-LAMP	Separate All comparator-positive; no specificity spectrum	No / No / No	Outside culture synthesis; retained in the comparator-specific paired map.
D127 OMD-2016-Petinataud	Separate Within-patient sample-quality stratum	No / No / Yes	Selected: eligible clinical nail detection-culture pair; largest analyzable N, then broadest target class, then frozen row-ID tie-break. Optimal and non-optimal sampling strata tie on N/target; optimal row is first in frozen register.
D128 OMD-2016-Petinataud	Separate Within-patient sample-quality stratum and structural comparator	No / No / No	Outside culture synthesis; retained in the comparator-specific paired map.
D129 OMD-2016-Petinataud	Separate Within-patient sample-quality stratum	No / No / No	Eligible alternative from same report; D127 retained by N, target breadth, frozen-ID hierarchy.
D130 OMD-2016-Petinataud	Separate Within-patient sample-quality stratum and structural comparator	No / No / No	Outside culture synthesis; retained in the comparator-specific paired map.
D131 OMD-2013-Miyajima	Separate Microscopy-positive spectrum; threshold reconstruction	No / No / No	Microscopy-positive selection.
D132 OMD-2013-Miyajima	Separate All comparator-positive; no specificity spectrum	No / No / No	Outside culture synthesis; retained in the comparator-specific paired map.
D133 OMD-2012-Luk	Main candidate Complete eight-pattern table	Yes / Yes / Yes	Selected: eligible clinical nail detection-culture pair; largest analyzable N, then broadest target class, then frozen row-ID tie-break.
D134 OMD-2012-Luk	Separate Structural comparator	No / No / No	Outside culture synthesis; retained in the comparator-specific paired map.
D135 OMD-2009-Ebihara	Separate All comparator-positive; first-round assay	No / No / No	Outside culture synthesis; retained in the comparator-specific paired map.
D136 OMD-2009-Ebihara	Separate All comparator-positive; nested assay	No / No / No	Outside culture synthesis; retained in the comparator-specific paired map.
D137 OMD-2010-Litz	Main candidate Complete four-test table	Yes / Yes / Yes	Selected: eligible clinical nail detection-culture pair; largest analyzable N, then broadest target class, then frozen row-ID tie-break.
D138 OMD-2010-Litz	Separate Structural comparator and target mismatch	No / No / No	Outside culture synthesis; retained in the comparator-specific paired map.
D139 OMD-2010-Litz	Separate Structural comparator and target mismatch	No / No / No	Outside culture synthesis; retained in the comparator-specific paired map.
D140 OMD-2018-Adel-Taha	Main candidate Target-matched culture endpoint	Yes / Yes / Yes	Selected: eligible clinical nail detection-culture pair; largest analyzable N, then broadest target class, then frozen row-ID tie-break.
D141 OMD-2018-Adel-Taha	Separate Structural comparator	No / No / No	Outside culture synthesis; retained in the comparator-specific paired map.
D142 OMD-2011-Brasch	Main candidate Exact species-matched nail subgroup	Yes / Yes / Yes	Selected: eligible clinical nail detection-culture pair; largest analyzable N, then broadest target class, then frozen row-ID tie-break.
D143 OMD-2025-Wang-RPA	Main candidate Target-matched culture endpoint	Yes / Yes / Yes	Selected: eligible clinical nail detection-culture pair; largest analyzable N, then broadest target class, then frozen row-ID tie-break.
D144 OMD-2025-Wang-RPA	Separate Structural comparator and target mismatch	No / No / No	Outside culture synthesis; retained in the comparator-specific paired map.
D145 OMD-2012-Dhib	Separate Composite includes contaminated culture category	No / No / No	Outside culture synthesis; retained in the comparator-specific paired map.
D146 OMD-2012-Dhib	Separate Structural comparator	No / No / No	Outside culture synthesis; retained in the comparator-specific paired map.
D147 OMD-2012-Dhib	Main candidate Target-matched culture endpoint	Yes / Yes / Yes	Selected: eligible clinical nail detection-culture pair; largest analyzable N, then broadest target class, then frozen row-ID tie-break.
D148 OMD-2019-Koo	Main candidate Target-matched culture endpoint	Yes / Yes / Yes	Selected: eligible clinical nail detection-culture pair; largest analyzable N, then broadest target class, then frozen row-ID tie-break.
D149 OMD-2019-Koo	Separate Adjudicated reference partly incorporates index test	No / No / No	Outside culture synthesis; retained in the comparator-specific paired map.
D150 OMD-2019-Koo	Separate Species-specific overlapping target row	No / No / No	Eligible alternative from same report; D148 retained by N, target breadth, frozen-ID hierarchy.
D151 OMD-2019-Koo	Separate Species/genus-specific overlapping target row	No / No / No	Eligible alternative from same report; D148 retained by N, target breadth, frozen-ID hierarchy.
D152 OMD-2020-Ross	Main candidate Target-matched nail culture endpoint	Yes / Yes / Yes	Selected: eligible clinical nail detection-culture pair; largest analyzable N, then broadest target class, then frozen row-ID tie-break.
D153 OMD-2020-Ross	Separate Structural comparator	No / No / No	Outside culture synthesis; retained in the comparator-specific paired map.
D154 OMD-2020-Ross	Separate Composite conventional endpoint	No / No / No	Outside culture synthesis; retained in the comparator-specific paired map.
D155 OMD-2021-Lin	Main candidate Target-matched nail culture endpoint	Yes / Yes / Yes	Selected: eligible clinical nail detection-culture pair; largest analyzable N, then broadest target class, then frozen row-ID tie-break.
D156 OMD-2021-Lin	Separate Structural comparator	No / No / No	Outside culture synthesis; retained in the comparator-specific paired map.
D157 OMD-2021-Lin	Separate Reconstructed nail-specific union	No / No / No	Outside culture synthesis; retained in the comparator-specific paired map.
D158 OMD-2021-Lin	Separate Culture-positive spectrum selection; overlaps study report only	No / No / No	Culture-positive validation selection; prospective clinical cohort D155 is available.
D159 OMD-2021-Cuchi-Burgos	Separate Structural comparator; 10 invalid qPCR excluded	No / No / No	Outside culture synthesis; retained in the comparator-specific paired map.
D160 OMD-2021-Cuchi-Burgos	Separate Target mismatch plus KOH-enriched sampling	No / No / No	KOH-enriched sampling (3:1 positive) and molecular dermatophyte/Candida versus dermatophyte-culture target mismatch.
D161 OMD-2026-Sharma	Main candidate Culture endpoint with off-panel species caveat	Yes / Yes / Yes	Selected: eligible clinical nail detection-culture pair; largest analyzable N, then broadest target class, then frozen row-ID tie-break.
D162 OMD-2026-Sharma	Separate Structural comparator	No / No / No	Outside culture synthesis; retained in the comparator-specific paired map.
D163 OMD-2004-Menotti	Main candidate Same optimally sampled specimen; composite comparator	No / No / No	Outside culture synthesis; retained in the comparator-specific paired map.
D164 OMD-2004-Menotti	Separate Sampling-location/quality mismatch by design	No / No / No	Outside culture synthesis; retained in the comparator-specific paired map.
D165 OMD-2007-Gupta-DoublePCR	Separate Assay-variant row; spectrum enriched	No / No / No	Prior *T. rubrum* culture-positive enrichment with mixed treatment exposure.
D166 OMD-2007-Gupta-DoublePCR	Separate Assay-variant row; spectrum enriched	No / No / No	Prior *T. rubrum* culture-positive enrichment with mixed treatment exposure.
D167 OMD-2007-Gupta-DoublePCR	Main candidate Target-matched culture endpoint; spectrum enriched	Yes / Yes / No	Prior *T. rubrum* culture-positive enrichment with mixed treatment exposure.
D168 OMD-2007-Gupta-DoublePCR	Separate Structural comparator and target mismatch	No / No / No	Outside culture synthesis; retained in the comparator-specific paired map.
D169 OMD-2007-Gupta-DoublePCR	Separate Structural comparator and target mismatch	No / No / No	Outside culture synthesis; retained in the comparator-specific paired map.
D170 OMD-2007-Gupta-DoublePCR	Separate Structural comparator and target mismatch	No / No / No	Outside culture synthesis; retained in the comparator-specific paired map.
D089V OMD-2019-Hayette	Revision valid-only derived variant; post hoc removal of eight inhibited PCR results from original D089; not a new study or independent comparison	No / No / Yes	Selected: eligible clinical nail detection-culture pair; largest analyzable N, then broadest target class, then frozen row-ID tie-break.
D171 OMD-2024-Felek	Not in frozen extraction; retrieved September 21, 2026; new full-text assessment after historical scope exclusion	No / No / No	Excluded from broader culture synthesis: selection on culture and/or molecular positivity; mixed prior treatment. Retained as a restricted paired cohort. Abstract reports 14 culture-positive specimens; Table 2 and Results, p. 1134, establish 15 (14 single-organism plus one dual-organism culture). Conclusion 65.2% culture-negative does not match the displayed cohort. Table 2 governs extracted cells.
D172 OMD-2024-Felek	Not in frozen extraction; retrieved September 21, 2026; new full-text assessment after historical scope exclusion	No / No / No	Excluded from broader culture synthesis: selection on culture and/or molecular positivity; mixed prior treatment. Retained as a restricted paired cohort. Abstract reports 14 culture-positive specimens; Table 2 and Results, p. 1134, establish 15 (14 single-organism plus one dual-organism culture). Conclusion 65.2% culture-negative does not match the displayed cohort. Table 2 governs extracted cells.
D173 OMD-2024-Felek	Not in frozen extraction; retrieved September 21, 2026; new full-text assessment after historical scope exclusion	No / No / No	Excluded from broader culture synthesis: selection on culture and/or molecular positivity; mixed prior treatment. Retained as a restricted paired cohort. Abstract reports 14 culture-positive specimens; Table 2 and Results, p. 1134, establish 15 (14 single-organism plus one dual-organism culture). Conclusion 65.2% culture-negative does not match the displayed cohort. Table 2 governs extracted cells.
D174 OMD-2024-Felek	Not in frozen extraction; retrieved September 21, 2026; new full-text assessment after historical scope exclusion	No / No / No	Excluded from broader culture synthesis: selection on culture and/or molecular positivity; mixed prior treatment. Retained as a restricted paired cohort. Abstract reports 14 culture-positive specimens; Table 2 and Results, p. 1134, establish 15 (14 single-organism plus one dual-organism culture). Conclusion 65.2% culture-negative does not match the displayed cohort. Table 2 governs extracted cells.

Potential cohort dependence was examined conservatively and was not resolved by assuming that distinct articles represent distinct people. Dhib 2012 and 2014 share the institution, 201 clinical nails, 80 controls, and conventional-test marginals; the larger eligible denominator was retained, and the alternative report was then substituted in a separate analysis. A possible Bako laboratory family comprises Gustafson 2019, Gupta NDM 2023, and Katsiaunis 2025; the conservative analysis retained only its largest selected report. These exclusions test uncertainty and are not claims that all specimens overlapped. The historical 40-row set and one-per-report subset use the original reported cells, including D089. The broader set uses D089V, reconstructed after removing eight inhibited results from the Hayette culture comparison.

The historical b/N column label and zero-ratio suppression were corrected explicitly in the revision analysis fields; original source cells and tags remain preserved. All individual intervals use the paired discordance counts, with the conditional assumptions stated in Appendix 2. No continuity correction, inferential pooling, disease-accuracy ranking, formal certainty grading, or quantitative publication-bias test was introduced.

Appendix 5

Exclusions and Search Update

During revision, two historical scope decisions were reassessed. PMID 38868878 was retrieved on 21 September 2026 and included after full-text assessment; its positive-test-selected cohort contributes restricted paired evidence (D171-D174), not the broader culture summary [8]. CC060, identified as Shahat AK et al. (2014), “Evaluation of Pan-Dermatophyte Nested PCR in Diagnosis of Onychomycosis in Comparison with Direct Microscopy and Culture” (reported DOI 10.12816/0025922), remained unavailable after documented retrieval attempts. It is counted as a report sought but not retrieved, with eligibility unresolved, rather than a full-text exclusion. No outcome data from that unavailable report enter the evidence map (Tables 14-15).

**Table 14:** Reports excluded after full-text assessment in the historical review register.

Report and source	Title	Recorded exclusion reason
OMD-2002-Tsuboi [105]	Identification and viability assessment of dermatophytes infecting nail based on quantitative PCR of dermatophyte actin (ACT) mRNA.	Secondary/symposium summary with no extractable nail-specific paired primary dataset
OMD-2022-Pearce [106]	Fungal Nails? DNA Facts Challenge Dystrophic Etiology.	No eligible conventional comparator or explicitly linked diagnostic reference standard
OMD-2015-Kobylak [107]	Real-time PCR approach in dermatophyte detection and Trichophyton rubrum identification.	Analytical/laboratory validation without eligible clinical cohort
OMD-2013-Pankewitz [108]	Development of a novel polymerase chain reaction-enzyme-linked immunosorbent assay for the diagnosis of Trichophyton rubrum onychomycosis.	Wrong population / no separately extractable nail data

**Table 15:** Documented PubMed update during peer-review revision. ITS: Internal Transcribed Spacer; PMID: PubMed Identifier.

Date and operation	Yield and deduplication	New record and decision
September 20, 2026; identical complete P1 query (Table 1), with no language or publication-date filters	309 records; all 308 original PMIDs retained; one additional PMID	PMID 42733718, Rueda-Jaime L et al. [109]. Excluded at the title/abstract level: single case involving ITS sequencing of a cultured isolate rather than an eligible direct nail-material diagnostic comparison.

This update did not repeat the Google Scholar or citation searches; their source-specific dates remain as stated in Table 1. The PubMed retrieval response and new-record abstract are preserved with the analysis materials. Following the separately documented full-text reassessment, the totals are 309 PubMed records screened, 229 excluded before central full-text assessment, and 80 assessed in full text. Across all streams, 102 reports were sought, one was not retrieved, 101 were assessed, and 97 were included. The Felek R et al. reassessment changes eligibility accounting, not the number of records found by the search update.
